# Unveiling reductant chemistry in fabricating noble metal aerogels for superior oxygen evolution and ethanol oxidation

**DOI:** 10.1038/s41467-020-15391-w

**Published:** 2020-03-27

**Authors:** Ran Du, Jinying Wang, Ying Wang, René Hübner, Xuelin Fan, Irena Senkovska, Yue Hu, Stefan Kaskel, Alexander Eychmüller

**Affiliations:** 1 Physical Chemistry, Technische Universität Dresden, Bergstr. 66b, 01069 Dresden, Germany; 2 Network for Computational Nanotechnology, Purdue University, West Lafayette, IN 47907 USA; 3 College of Chemistry and Materials Engineering, Wenzhou University, 325000 Wenzhou, China; 4 Helmholtz-Zentrum Dresden-Rossendorf, Institute of Ion Beam Physics and Materials Research, Bautzner Landstrasse 400, 01328 Dresden, Germany; 5 Department of Inorganic Chemistry, Technische Universität Dresden, Bergstr. 66b, 01062 Dresden, Germany

**Keywords:** Physical chemistry, Porous materials, Gels and hydrogels, Self-assembly

## Abstract

Amongst various porous materials, noble metal aerogels attract wide attention due to their concurrently featured catalytic properties and large surface areas. However, insufficient understanding and investigation of key factors (e.g. reductants and ligands) in the fabrication process limits on-target design, impeding material diversity and available applications. Herein, unveiling multiple roles of reductants, we develop an efficient method, i.e. the excessive-reductant-directed gelation strategy. It enables to integrate ligand chemistry for creating gold aerogels with a record-high specific surface area (59.8 m^2^ g^−1^), and to expand the composition to all common noble metals. Moreover, we demonstrate impressive electrocatalytic performance of these aerogels for the ethanol oxidation and oxygen evolution reaction, and discover an unconventional organic-ligand-enhancing effect. The present work not only enriches the composition and structural diversity of noble metal aerogels, but also opens up new dimensions for devising efficient electrocatalysts for broad material systems.

## Abstract (web summary)

Non-efficient gelation methods for noble metal particles limit the development of the corresponding gel materials. Here the authors describe the role of reductants, unlocking ligand chemistry, and largely expanding the composition space of noble metal aerogels for high-performance electrocatalysis.

## Introduction

Along with the interest in nanoscience shifting from individual nanoparticles (NPs) to their meso-/macroscopic ensembles, a library of NP-based assemblies appeared in the last few years^1^. Among them, aerogels draw substantial interest because of their unique structural features including large specific surface areas (SSAs), self-supported architectures, and three-dimensional (3D) porous structures^2–4^. As a newcomer, noble metal aerogels (NMAs) debuted in 2009^5–7^. Imparting the unique physicochemical properties of nanostructured noble metals to aerogels, NMAs manifest abundant catalytically/optically active sites, rapid mass/electron transfer channels, and excellent structural stability, confirming their broad application prospects such as catalysis and surface-enhanced Raman scattering^8–11^. Particularly in electrocatalysis, NMAs exhibit extraordinary activities surpassing commercial noble metal catalysts^11–16^. However, the underlying structure-performance correlations are not well understood, presumably because an insufficient understanding of the sol-gel process retards manipulating versatile parameters (e.g., ligament sizes, compositions, and spatial element distributions)^6,7^. Therefore, the development of efficient fabrication strategies and in-depth investigations of the underlying gelation mechanisms are of utmost importance.

Generally, the gelation is a process where the initial colloidal solution is destabilized and transformed into a 3D self-supported gel. Noble metal systems display distinct gelation behaviors compared with other materials (e.g., silica, polymers, and nanocarbons), thus requiring new fabrication approaches^3,17,18^. Typically, two type methods are applied. The first class is the two-step methods, comprising of separate NP synthesis and assembly steps. The decoupling of the two steps facilitates subtle design of the nano-building blocks and the application of diverse destabilization methods^5,16,19–22^, thus affording widespread compositions (Au, Ag, Pd, Pt, and their multi-metallic variants)^5,16^, tunable structures^5,11,14,16,22^, and the ease for studying gelation mechanisms^16^. However, considering complicated procedures and unavoidable impurities, the second-class methods, i.e., the one-step methods have been developed by Liu et al.^23^, in which gels were fabricated directly from metal salt solutions by introducing a specific amount of reductants. This approach combines the metal salt reduction (i.e., NP synthesis) and gel formation (i.e., NPs assembly) in one step^12,24–27^. Despite simplicity, the gel parameters are less adjustable and the gelation process is sluggish (a few weeks at room temperature). Moreover, coupled NP synthesis and assembly poses a major hurdle in devising building blocks and deciphering underlying gelation mechanisms. On the other hand, the ligand-directed synthesis, which was extensively explored in nanocrystal field^28,29^, cannot be implemented in preparing gels. Because the insufficient power of the current destabilizing methods fails to work with many ligand-involved systems^12^, thus reducing a potential dimension to tailor NMAs. To address these issues, it is essential to develop new one-step methods with strong destabilization and modulation capacity, as well as to gain a deep understanding of the reductant and ligand chemistry in the gelation process.

Here we show, an excessive-reductant-directed gelation strategy fitting both the one-step and two-step methods. We unveil the anion effects and multi-functions of reductants (i.e., as reducing agents, stabilizers, and initiators), and disclose an overall gelation picture initiated by NaBH_4_ via combined experimental and theoretical approaches. Taking advantage of the unprecedented destabilization capacity of this method, we acquire gold aerogels with a record-high specific surface area (59.8 m^2^ g^−1^) by activating the ligand chemistry, and expand the composition space to all 8 noble metals (Au, Ag, Pd, Pt, Ru, Rh, Os, and Ir), thus discovering new phenomena (i.e., spontaneous combustion) and obtaining various high-performance electrocatalysts for the ethanol oxidation reaction (EOR) and oxygen evolution reaction (OER). Moreover, we demonstrate an unconventional organic-ligand-enhancing effect to substantially improve the electrocatalytic performance. Therefore, the present work gains an insightful understanding of the gelation mechanisms, enriches the compositions of NMAs, and opens up new dimensions for devising high-performance electrocatalysts. These findings should adapt to diverse material systems such as hydrogels, aerogels, unsupported porous materials, and nanocrystals.

## Results

### Synthesis and characterization of gold aerogels

**Fig. 1:**
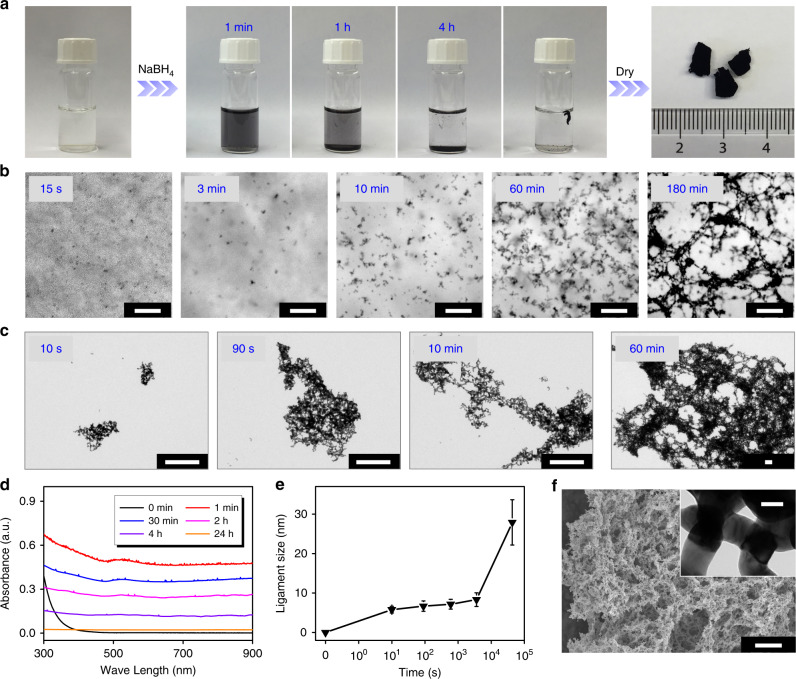
Gelation process of gold NPs triggered by NaBH_4_. **a** Photographs of the fabrication process of the gold aerogel, where the wet gel was directly obtained from the HAuCl_4_ aqueous solution (5 mL) and further freeze-dried to yield the corresponding aerogel. Note for a clear demonstration, the aerogels shown in Fig. 1a were prepared from ~800 mL HAuCl_4_ solution. Time-lapse **b** in situ optical microscopy, **c** ex situ TEM, and **d** UV-vis absorption spectra monitoring the gelation process. **e** Ligament size evolution of the gold aggregates during the gelation process (error bars depict the standard deviation). **f** SEM image and TEM image (inset) of the resulting gold aerogel. The scale bars in **b**, **c**, **f**, and the inset in **f** are 20 μm, 200 nm, 10 μm, and 20 nm, respectively.

Detailed procedures can be found in Supplementary Methods. As an example, gold aerogels were prepared from HAuCl_4_ aqueous solutions via a NaBH_4_-induced sol-gel process followed by freeze-drying (Fig. 1a). The as-obtained hydrogel floated in the solution, as driven by the generated H_2_ bubbles from NaBH_4_ inside the gel networks during the gelation process. In contrast to previous reports where the NaBH_4_-to-metal-salt ratio (R/M) was set to 1.5–5.0^12,23,25,26^, an excessive amount of NaBH_4_ (R/M = 100) was adopted here. The gel formed within 4–6 h at room temperature, substantially faster than that of previous NaBH_4_-triggered gelation systems (a few weeks at room temperature, and ~6 h at 333 K)^12,23^. The formation process was characterized by several time-lapse techniques as shown in Fig. 1b–e. Combined ex situ transmission electron microscopy (TEM) and in situ optical microscopy observations intuitively revealed the fast development of branched-nanowire (NW)-like gel networks at different length scales. The size of aggregates reached a few hundreds of nanometers within seconds and evolved to tens of micrometers in a few minutes. Meanwhile, the radial growth increased the average ligament size from a few nanometers to 27.9 nm (Fig. 1e), resembling the case of the salt-induced gelation^16^. The fast network evolution was additionally verified by in situ ultraviolet-visible (UV-vis) spectroscopy (Fig. 1d), where an immediately appearing broad-band absorption suggests the rapid formation of large nanostructured gold aggregates. Burrs in the spectra are attributed to the generated H_2_ bubbles from the reaction of NaBH_4_, and the time-dependent intensity decay can be explained by gravity-driven sedimentation of high-density gold aggregates^11,16^. Purified and dried, the resulting gold aerogels were characterized as highly porous 3D networks structured from fused NWs (Fig. 1f) with a yield of ~95.9%.

Compared with a number of strategies where the gelation resembles a precipitation process^13,14,16,21,23^, here, visible gel pieces occurred throughout the bulk solution upon initiation (Supplementary Fig. 1). This is ascribed to the disturbance of the reaction system by in situ generated H_2_ bubbles from excessive amount of NaBH_4_, due to either self-decomposition or gold-catalyzed decomposition from in situ generated Au aggregates (Supplementary Fig. 2). To this end, large gel pieces were observed in the bulk solution and the gelation time was considerably shortened (ca. 2 h for obtaining a gel with *c*_M_ = 0.5 mM, Supplementary Figs. 2–3). Besides the one-step process, the as-developed method also fits the two-step gelation process, as NaBH_4_ can be applied to promote the gelation of gold NP solutions or to assist the salt-induced gelation (Supplementary Fig. 4). In the latter case, trace amounts of NaBH_4_ (R/M < 0.5) can trigger the gelation in the presence of Na_2_SO_4_ and modulate the morphology of the products.

### Multiple roles of the reductants

**Fig. 2:**
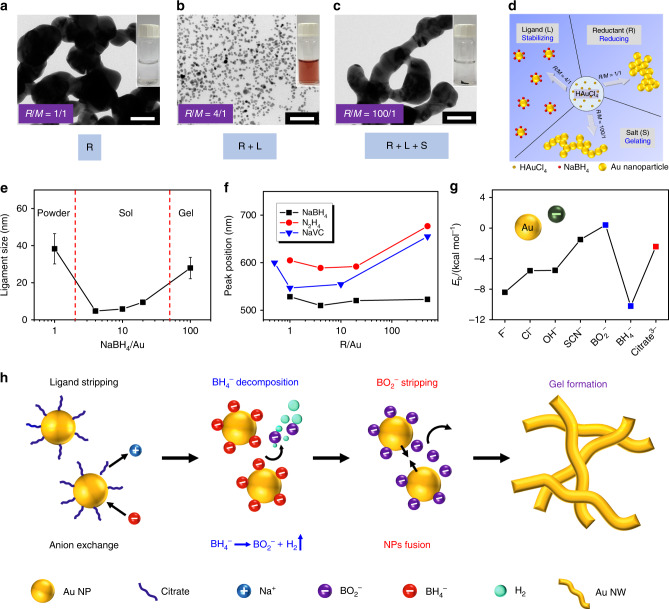
Analysis of the multiple-roles played by the reductant. **a**–**c** TEM images and photographs (insets) of the resulting gold materials for NaBH_4_/gold ratios of 1/1, 4/1, and 100/1, and **d** the function(s) of NaBH_4_ exhibited in the respective systems. **e** The ligament size of the gold gels vs. the NaBH_4_/gold ratio (error bars depict the standard deviation). **f** The peak position at maximum absorption (from UV-vis absorption spectra) vs. the reductant/gold ratio for different reductants. **g** Theoretically calculated binding energies between the gold atom and various anions. **h** The proposed mechanisms for the excessive NaBH_4_-directed gelation process. The scale bars are 50 nm in **a**–**c**.

The uniqueness of the current method drives us to in-depth study the possible roles played by NaBH_4_ and the underlying mechanisms of the reductant-directed gelation process. Here, gold systems (*c*_M_ = 0.2 mM) were taken to probe the reductant chemistry. Combining previous experience^12,23^ and the current results, evidently R/M ratio greatly impact the stability of metal-based colloidal solutions. As shown in Fig. 2a–e and Supplementary Fig. 5, for ligand-free HAuCl_4_/NaBH_4_ systems, either low or high R/M ratios (<2 or ≥50) destabilized solutions, while a medium R/M offers stable gold NP solutions. Compared to the high R/M case, a larger ligament size (38.7 vs. 27.9 nm) and a smaller amount of products were observed in the low R/M region. In addition, in the presence of ligands (i.e., trisodium citrate (NaCA)), the destabilization was inhibited for low R/M systems. Based on these results, multiple roles of the reductant are proposed in Fig. 2d. (1) In the low R/M region (R/M < 2), NaBH_4_ only functions as a reducing agent, where the generated less-protected gold NPs will fuse together to form large-sized aggregates. (2) In the medium R/M region (2 < R/M < 50), the residual NaBH_4_ after reducing entire gold salts can provide sufficient BH_4_^−^ (or BO_2_^−^ from the decomposition of NaBH_4_) to stabilize gold NPs, thus activating its second function, i.e., as a stabilizer^30^. (3) In the high R/M region (R/M ≥ 50), because of its electrolyte nature, a large excessive amount of NaBH_4_ will unlock its third function, i.e., the salting-out capability, thus initiating NP aggregation and fusion in the presence of in situ generated ligands (i.e., BH_4_^−^/BO_2_^−^). In this way, the roles of NaBH_4_ are determined by the R/M ratio, eventually governing the colloidal stability and gelation behavior. Since the salting-out function is activated to promote destabilization, it is not surprising to find a accelerated gelation process at room temperature in our systems compared to the commonly investigated low R/M systems (several hours vs. a few weeks)^23^.

To verify the aforementioned hypothesis, various reductants were tested (Supplementary Figs. 5, 6). Sodium ascorbate (NaVC), sodium hypophosphite monohydrate (NaH_2_PO_2_), and hydrazine monohydrate (N_2_H_4_), all of which can reduce gold salts and provide excessive free ions in water, gave similar results. In sharp contrast, non-dissociating reductants (e.g., ascorbic acid) which lack the salting-out function, can only reduce gold salts, but not destabilize the solution even at a R/M up to 200 (Supplementary Fig. 6). Moreover, recording the wavelength of the absorption maximum in the UV-vis spectra, a minimal wavelength was observed in the medium R/M region (Fig. 2f and Supplementary Fig. 7). From the widely accepted correlation between the size and geometry of gold NPs with the peak position^31^, it suggests that small NPs present in the medium R/M region while larger or geometrically anisotropic structures evolve at higher or lower R/M, in line with the proposed hypothesis. Thus far, the triple roles of reductants, i.e., (1) reducing agent, (2) stabilizer, and (3) initiator, are revealed. In this way, two latent functions of the reductants (ligand and initiator) were unlocked in the current system (R/M ≥ 100) compared with previously reported systems (R/M ≤ 5), affording faster fabrication of gold gels with smaller ligament size.

In the current system, the salting-out function provided by NaBH_4_ is remarkably more powerful in comparison with previously reported salt-induced gelation approaches^16^. Previously we found that cations from disassociated salts have strong influence on the gelation process, because they can electrostatically strip away negatively-charged ligands from the NP surfaces at different extent, thus tuning the mode of NP fusion. Here, considering the distinct gelation behavior induced by NaBH_4_ compared to other common sodium-based salts (e.g., Na_2_SO_4_), an anion effect needs to be considered, too. We propose that anions from the salts can function as inorganic ligands as reported in the field of semiconductor nanocrystals^32^.

First, common salts are considered. Taking the gelation of NaCA-stabilized gold NPs as an example, upon addition of excessive salts, the NPs will approach each other due to the salting-out effect^16^, meanwhile anions can replace the negatively-charged citrates on the NPs via a ligand-exchange step. In comparison with citrates, the smaller-sized salt anion ligands can render a reduced distance between the NPs, thus promoting destabilization via boosting van der Waals attractive interactions and effective collisions between NPs^33^. As a support from density functional theory (DFT) calculations shown in Fig. 2g and Supplementary Fig. 8, the binding energies (*E*_b_) between gold atoms and anions (F^−^, Cl^−^, OH^−^, SCN^−^, 1.51–8.40 kcal mol^−1^) generally follow the Hofmeister series^34^ and are larger or similar to that of citrate^3−^ (2.42 kcal mol^−1^), which facilitates ligand exchange especially if an excessive amount of anions are applied. Driven by a large energy decrease from the fusion between gold atoms, these anion-stabilized NPs can easily discard “small ligands” through a dynamic attaching-detaching process, fusing to 3D networks (calculated reaction energies *E*_r_ of the fusion: 2 Au-anion → Au-Au + 2 anions, are located in −29.3 to −53.2 kcal mol^−1^, confirming a thermodynamically favorable process). Considering that the employed ligand (i.e., NaCA) is also an electrolyte being weakly bonded on the Au NPs and its size is not large, it should possess potential salting-out function according to the proposed hypothesis (Supplementary Figs. 8, 9). As expected, self-supported gels were obtained by applying an excessive amount of NaCA (NaCA/Au = 500/1). Hence, aside from the known role as ligand, reductant, and size controller^35^, a concealed role (i.e., as salting-out agent) of NaCA was discovered, which may provide new insight for the ligand chemistry and offer new pathways for nanomaterial engineering.

Secondly, when NaBH_4_ was selected as salting-out agent, the situation is more complicated yet more interesting, which can be due to the in situ transformation of NaBH_4_ to NaBO_2_ and H_2_ from both the self-decomposition and the gold-assisted decomposition of NaBH_4_ during the gelation process (see ref. ^36^). and Supplementary Fig. 2). Again, for destabilizing NaCA-capped NPs (Fig. 2h), cations (Na^+^) will strip^16^ while anions (BH_4_^−^) will exchange with citrate^3−^. Notably, due to its high affinity to gold (*E*_b_ = 10.3 kcal mol^−1^ for BH_4_^−^), the ligand exchange process with BH_4_^−^ should be much more effective than for common anions (1.51–8.40 kcal mol^−1^). This is also evidenced from zeta potential measurements, where a more negative zeta potential was obtained with increasing NaBH_4_/Au ratio (Supplementary Fig. 10). Ascribed to the decomposition, the exchanged BH_4_^−^ on the NP surfaces will in situ transform to BO_2_^−^ and concurrently generate H_2_ bubbles surrounding NPs. Intriguingly, BO_2_^−^ ions feature a very low bonding strength to gold (*E*_b_ = 0.397 kcal mol^−1^), thus will easily detaching from NPs and promoting destabilization; meanwhile, H_2_ bubbles, in situ generated exactly on the NP surfaces, can strongly promote collisions between NPs. In this way, excessive NaBH_4_ can serve as a unique and powerful gelation initiator, substantially accelerating the gelation process compared with previously reported methods (e.g., by using common salts, a small amount of NaBH_4_, or other additives)^5,12,13,16,20,21,37^. Regarding the one-step gelation of the HAuCl_4_ solution with an excessive amount of NaBH_4_, gold NPs will be produced before and during the aforementioned process, while the general story would be similar. Hereto, multiple funcitons of NaBH_4_ were unveiled, i.e., in situ introducing weakly-bonded anions (i.e., BO_2_^−^) and H_2_ bubbles on the NP surfaces, the synergy of which greatly stimulates the destabilization process.

To this end, the multi-roles of the reductants were comprehensively investigated. Via the exploration of anion effects, a deep understanding for the uniqueness and the exceptional destabilization capacity of NaBH_4_ was gained, and an overall picture depicting the excessive NaBH_4_-directed gelation process was presented. Moreover, in light of the intepretation from both cations and anions aspects based on this and previous work^16^, a complete mechanism of the salt-directed gelation process was unveiled. 

### Engineering of noble metal gels

On the basis of a comprehensive investigation of the reductant chemistry, as well as the overall gelation process, on-target manipulation of noble metal aerogels is presented.

First, ligand chemistry was explored to modulate gold aerogels. Ligand-assisted material fabrication is widely adopted in nanocrystal synthesis, where the preferential adsorption of specific ligands can tune the facets and morphology of nanocrystals^28,29,38^. Therefore, if incorporated, it will offer profound opportunities to direct anisotropic NP fusion and thus modulating the ligament sizes. As seen from Supplementary Figs. 11–13, the NaBH_4_-directed gelation method (R/M = 100) adapts to a wide range of ligand-stabilized gold NPs (e.g., NaCA, β-alanine, sodium deoxycholate (NaDC), 2-mercaptopropionic acid (MPA), polyvinylpyrrolidone (PVP), and cetyltrimethyl ammonium bromide (CTAB)). Notably, despite a relatively weak bonding to gold (Supplementary Fig. 8), PVP can strongly stabilize NPs by steric effects due to its large size^28,38^. Therefore, PVP-stabilized gold NPs were unaffected by almost all previously reported gelation methods (e.g., by using elevated temperature, H_2_O_2_, NH_4_F, CaCl_2_, dopamine, or ethanol^5,13,16,20,21,37^). The remarkable destabilization power delivered by the current method can be interpreted from the as-discussed mechanism, which allows effectively wiping out PVP via the combined BH_4_^−^ exchange/transformation and H_2_ bubbling. The ligand exchange process is indirectly supported by Zeta potential tests, where the potential became increasingly negative with prolonging reaction time (Supplementary Fig. 10), suggesting successful replacement with the negatively-charged anions. In addition, a comparative study shows that gas bubbles (either N_2_ or H_2_) introduced after the particle synthesis cannot destabilize PVP-protected NP solutions in the presence of salts (Supplementary Fig. 14), indicating the necessity of synchronized in situ bubble generation and special ligand exchange/transformation processes.

**Fig. 3:**
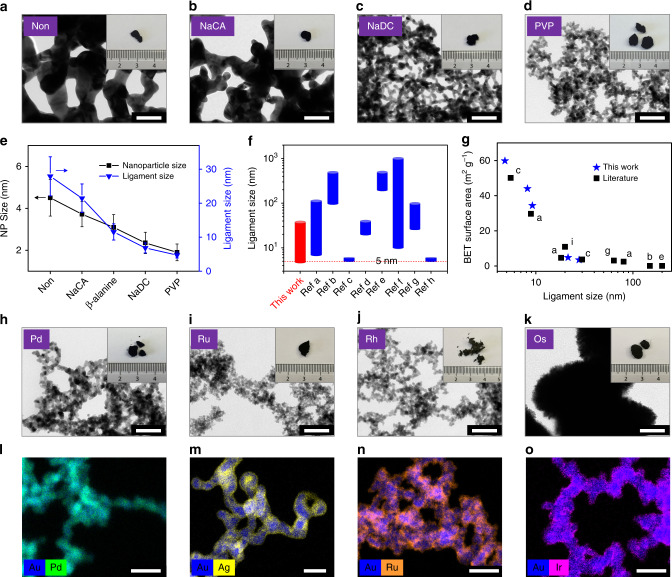
Versatile manipulation of NMAs. **a**–**d** Gold gels fabricated by destabilizing different-ligands-stabilized gold NPs with NaBH_4_, TEM images and photographs (insets). **e** The size of different-ligands-stabilized gold NPs and the ligament size of the corresponding gold aerogels (as derived from TEM analysis, error bars depict the standard deviation). A comparison of **f** the ligament size and **g** the ligament size vs. BET surface area of gold aerogels reported in literature, where panels **a**–**i** correspond to the references^5,16,21,24,39–43^, respectively. TEM images and photographs (insets) of **h** Pd, **i** Ru, **j** Rh, and **k** Os aerogels. STEM-EDX maps of **l** Au-Pd, **m** Au-Ag, **n** Au-Ru, and **o** Au-Ir aerogels. The scale bars are 50 nm in **a**–**d**, **h**–**k**, and are 20 nm in **l**–**o**.

The original size of the NPs and the ligament size of gels were probed by TEM, scanning electron microscopy (SEM), and X-ray diffraction (XRD) (Fig. 3a–e, Supplementary Figs. 15–17). Both sizes are strongly influenced by the specific ligands and follow a similar trend. The distinct differences in ligament size can be ascribed to the stabilization ability of various ligands^29^, where the stronger ones (e.g., NaDC, PVP, MPA, and CTAB) can cut down the size below 10 nm, while the others render the size to 10−30 nm (Supplementary Fig. 17). In addition, the ligament size is positively related to *c*_M_. Impressively, the presence of PVP can restrict the average ligament size to <11 nm within a large range of *c*_M_ (0.2−5 mM) (Supplementary Fig. 18), attributing to its strong stabilization ability and the shape-directing function^28^. This allows for a water-based mass production of nanostructured gold gels from a much higher HAuCl_4_ concentration (up to 5 mM) compared to previous reports (typically 0.2–0.6 mM)^5,11–13,16,20,21^. Regulating the ligament sizes can further render the specific surface areas, pore size distributions, and pore volumes (Supplementary Fig. 19, Supplementary Table 1). Strikingly, a substantially small ligament size of down to 4.8 ± 0.9 nm was achieved by destabilizing PVP-protected gold NPs, affording a gold aerogel with a record-high specific surface area of 59.8 m^2^ g^−1^. As shown in Fig. 3f-g, Supplementary Table 2, this value is higher than that of gold/β-cyclodextrin composite aerogels (50.1 m^2^ g^−1^)^21^ and NH_4_SCN-prepared gold aerogels (29.7 m^2^ g^−1^)^16^, and remarkably surpasses all other gold aerogels/foams reported to date (<10 m^2^ g^−1^ on average)^5,24,39–43^. Notably, the gelation process can also proceed in an one-step manner, i.e., directly performing in HAuCl_4_/ligand aqueous solutions with NaBH_4_. Notably, following washing steps can remove almost all ligands present in the final aerogels (Supplementary Fig. 20), facilitating investigation of intrinsic properties of NMAs.

Second, the composition space of NMAs is largely expanded in virtue of the strong gelation power of the as-developed method. Only four single-metallic NMAs (Au, Ag, Pd, Pt) have been reported to date^6,7^, while the other common noble metal elements (Ru, Rh, Os, Ir) only appeared in transition-metal-containing multi-metallic aerogels in a few cases (e.g., Ir-Cu)^26^. As seen from Supplementary Fig. 21, all 8 single noble metal NP solutions can be made and subsequently destabilized with excessive NaBH_4_, and 7 of them formed self-supported gels (only the Ir system resulted in unsupported powders). In sharp contrast, most previous approaches (heating, H_2_O_2_, CaCl_2_, dopamine, and NH_4_F^5,16,20,21,37^) failed for the less-investigated noble metal systems (Ru, Rh, Os, Ir), only dopamine-based method can create the Rh and Os gels though the products from this may suffer from serious contamination^21^. These results further confirm the unparalleled advantages of the excessive-NaBH_4_-based method. From TEM, SEM, XRD, and nitrogen adsorption characterizations (Fig. 3h-k, Supplementary Figs. 22–25, Supplementary Table 1), all single-metallic aerogels displayed highly porous structures with various ligament sizes (3.9–226.7 nm), SSAs (2.1–69.1 m^2^ g^−1^), pore volumes (0.058–0.357 cm^3^ g^−1^), and densities (49.5–250.6 g cm^−3^). Ag, Pd, Ru, and Rh aerogels showcased nanowire-like structures similar to those of the Au counterpart; Pt and Os aerogels displayed hierarchical structures, with the large-sized backbones (>80 nm) composed of nano-sized particles (<10 nm). Despite small surface areas for certain samples, they are regarded as aerogels because they feature 3D interconnected networks that offer them monolithic architectures extending to the macroscale (e.g., >1 mm). Intriguingly, a spontaneous combustion phenomenon was observed for the Ru and Rh single-metallic aerogels. Once dried, Ru and Rh gels were very likely to spontaneously burn out in air, leaving behind powder-like pieces characterized as partially oxidized species with considerably increased ligament sizes and very low SSA (Supplementary Fig. 26). This might be attributed to a combination of the high activity of Ru and Rh toward oxidation and the high SSAs of the freshly prepared aerogels, which might found applications as energetic materials (e.g., propellants) once the combustion can be appropriately controlled.

To impart more functions and to utilize the high conductivity of gold, a wide range of gold-based bimetallic NMAs, including Au-Ag, Au-Pd, Au-Pt, Au-Ru, Au-Rh, and Au-Ir were prepared by one-pot destabilizing mixed metal salts with excessive NaBH_4_. Besides gold-based bimetallic NMAs, various other NMAs such as Ag-Pd, Ag-Rh, Pd-Pt, and Pd-Rh aerogels can also be easily prepared. These aerogels were characterized by various techniques as shown in the Supplementary Figs. 27–31, Table 1. All freshly prepared aerogels display small ligament sizes (4.3–7.6 nm), large SSAs (42.6–72.9 m^2^ g^−1^), high pore volumes (0.206–0.610 cm^3^ g^−1^), and predominant mesopores (2–30 nm). By imparting gold, the resulting bimetallic Au-Rh aerogel was stable in air, while the Au-Ru aerogel still tended to burn spontaneously and thus exhibiting a very low SSA (1.2 m^2^ g^−1^). Notably, despite the strong destabilization power of NaBH_4_, the Ir salt was extremely difficult to be reduced and incorporated into a gel. As a result, the yield for the Au-Ir aerogel was less than 60% (with 10–30 wt.% Ir as characterized by inductively coupled plasma optical emission spectroscopy (ICP-OES)), much lower than the yield for other systems (>85%). For the bimetallic systems, the spatial element distribution is another important parameter governing material properties. As explored by scanning transmission electron microscopy energy-dispersive X-ray spectroscopy (STEM-EDX) shown in Fig. 3l–o and Supplementary Figs. 32 and 33, different bimetallic systems show distinct elemental distributions. Despite a one-pot synthesis without special design, the Au-Pd gel featured quasi-homogeneous distribution, while the Au-Ag, Au-Ru, Au-Rh, and Au-Ir gels featured pronounced core-shell structures with shell thicknesses of 1–3 nm. From the elemental analysis, the slightly higher proportion of the “shell metal” as determined by X-ray photoelectron spectroscopy (XPS) than by ICP-OES can partially reflect the core-shell structure of the corresponding aerogels due to the surface sensitivity of XPS^16^ (Supplementary Table 3). In addition, presumably due to the electronegativity difference of Au (2.54) and Pd (2.20), the peak of Au 4f 7/2 shifts negatively from 84.0 eV to 83.9 eV, while the peak of Pd 3d 5/2 shifts positively from 335.1 to 335.4 eV, suggesting an electron transfer from Pd to Au as a consequence of alloying. In contrast, no considerable peak shift was observed for Au-Ir and Au-Rh aerogels, indicating weak interactions between the two metals. It is known that silver tends to segregate to the surface of gold–silver alloys due to its lower surface energy^42^, while the segregation behavior of the other systems has been less investigated so far. One possible reason may be the slower reaction kinetics of Ru, Rh, and Ir salts compared to that of HAuCl_4_ (Supplementary Fig. 34), which resulted in the fast formation of gold cores on which the other metal will nucleate and grow^16^. However, further investigations are needed to unambiguously reveal the underlying mechanisms.

### Electrocatalytic performance of NMAs

Featuring abundant highly active sites, 3D mass/electron transfer channels, and stable interconnected networks, NMAs have been characterized as ideal candidates for electrocatalysis surpassing various non-metal-based, transition-metal-based, and commercial noble-metal-based electrocatalysts. In light of the flexible manipulation of NMAs as realized in this work, here two electrocatalytic reactions, i.e., ethanol oxidation reaction (EOR) and oxygen evolution reaction (OER) were investigated.

**Fig. 4:**
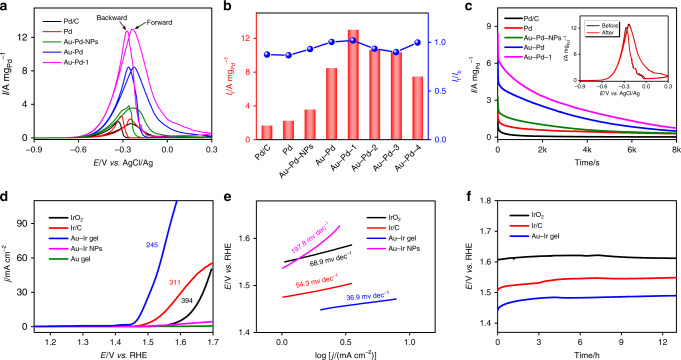
Electrocatalytic performance of EOR and OER. EOR performance: **a** CV curves (scan rate 50 mV s^−1^), **b** summarized *I*_f_ and *I*_f_/*I*_b_, and **c** chronoamperometry tests of commercial Pd/C, Au-Pd NPs, and various aerogel catalysts in 1.0 M KOH + 1.0 M ethanol solution. The inset of **c** shows the CV curves of Au-Pd-1 before and after a 3 h chronoamperometry test. OER performance: **d** LSV curves (scan rate 5 mV s^−1^), **e** Tafel plots, and **f** chronopotentiometric tests of commercial Ir/C, IrO_2_, Au aerogel, Au-Ir NPs, and Au-Ir aerogel in 1.0 M KOH aqueous solution.

The electrocatalytic ethanol oxidation, serving as a model reaction that takes place at the anode of direct ethanol fuel cells, has been extensively studied by using a wide range of Pd-based and Pt-based NMAs^6,7^ and nanowire networks^44,45^. EOR tests of commercial Pd/C were performed together with those on the Pd aerogel, Au-Pd aerogel, and Au-Pd NPs (Fig. 4a, b). All catalysts exhibit two peaks in the forward and backward scan, indicating the oxidation of ethanol and the further oxidation of intermediates generated during the forward scan, respectively^15,16,46^. The two most important parameters, i.e., the forward current density (*I*_f_) and the ratio of forward/backward current density (*I*_f_/*I*_b_), were evaluated. Commercial Pd/C, the Pd aerogel, and Au-Pd NPs delivered inferior *I*_f_ (1.68, 2.24, and 3.56 A mg_Pd_^−1^) and *I*_f_/*I*_b_ (0.87, 0.86, and 0.93) compared to the Au-Pd aerogel (*I*_f_ = 8.45 A mg_Pd_^−1^, *I*_f_/*I*_b_ = 1.00). The substantially higher *I*_f_ confirms the high-performance of the Au-Pd aerogel catalyst, presumably coming from the high conductivity of gold, the high catalytic activity of palladium, and the structural attributes provided by the aerogel. The *I*_f_/*I*_b_ of the aerogel-based and NP-based Au-Pd catalysts are similar, which can be attributed to similar reaction pathways resulting from the similar chemical compositions. The relatively low *I*_f_/*I*_b_ suggests that the intermediates generated during operation can deactivate the catalysts seriously, which might be addressed by modulating the compositions^16^. Furthermore, considering certain characteristics of surfactants (e.g., enhanced dispersing ability), PVP, a commonly used surfactant, was deliberately introduced during the catalyst ink preparation of the Au-Pd aerogels (denoted as Au-Pd-*x*, where *x* = 1, 2, 3, 4 represent a PVP concentration of 0.03, 0.1, 0.3, and 1 mM, respectively). Countering to intuition that surfactants will deactivate catalysts, a performance increase, in view of both *I*_f_ and *I*_f_/*I*_b_, was achieved for Au-Pd-1, realizing a striking *I*_f_ of 12.99 A mg_Pd_^−1^ and an *I*_f_/*I*_b_ of 1.02. The *I*_f_ is 7.7 and 1.5 times higher than that of the Pd/C and Au-Pd aerogel, respectively, surpassing most EOR catalysts such as Pd-Ni and Au-Pd-Pt aerogels (2.0–6.1 times compared to Pd/C)^7,16,36^, and only inferior to Pd-ensembles-anchored Au-Cu aerogels (*I*_f_ of 11.6 times higher than that of Pd black) and Pd-Au alloy nanowire networks^15,45^. Even if the mass activity is normalized to all metals, the Au-Pd-1 still features a considerably higher performance than that of Pd/C (3.79 vs. 1.68 A mg^−1^). The enhanced catalytic performance might be ascribed to a better dispersion of the aerogel and possible charge transfer between PVP and metals^38^. It is not surprising to see a performance decay with further increasing PVP, which can be rationalized by the blocking of active sites (Fig. 4b and Supplementary Fig. 35).

Similar to commercial Pd/C, the aerogel catalysts exhibit a serious current decay with prolonging time (Fig. 4c). The performance decay may result from the morphology change, the depletion of the reactants, or the reversible blocking of active sites by intermediates^46^. Because the performance fade of Au-Pd-1 almost fully recovered after CV cleaning (see inset diagram of Fig. 4c), an irreversible morphology change can be ruled out. This was also verified by less-changed morphologies after cycling, though the current decayed considerably (Supplementary Fig. 36). To evaluate the effect of mass transfer, the Au-Pd catalyst was prepared on a rotating disk electrode (RDE). As seen from Supplementary Fig. 35, *I*_f_ increased with the rotation speed and quickly reached a saturation at 400–900 rpm, while a higher rotation speed led to a gradual current decrease. In addition, chronoamperometry tests infer a more pronounced current decay at rotation condition (1600 rpm) compared with the static condition. These results suggest that the active sites are blocked by intermediates, which aggravates the performance at enhanced mass transfer conditions and accounts for the rapid current decay. While a considerable performance fade for all investigated materials, the stability of Au-Pd-1 greatly surpasses that of commercial Pd/C (51.7% vs. 10.6% current retention after chronoamperometry evaluation for 1000 s, see Supplementary Fig. 37). However, for practical applications, further efforts to improve the long-term stability are needed.

Aside from EOR, the electrocatalytic oxygen evolution performance of NMAs was also investigated. The OER is an important anode reaction involved in electrochemical water splitting and metal-air batteries. Because of its sluggish kinetics, the OER usually features a high overpotential to reach a reasonable current, thus causing a large energy loss for practical operation. Ir-based nanomaterials are regarded as state-of-the-art OER catalysts^26^, while Ir-based 3D networks, especially composed by solely noble metals, were less investigated. Here, the core-shell structured Au-Ir aerogel was applied for OER, expecting that synergy of the highly conductive gold core-network and the exposed active Ir shell could enhance the OER performance. As shown in Fig. 4d–f, Au-Ir core-shell aerogels manifested a considerably small overpotential and Tafel slope (245 mV and 36.9 mV dec^−1^), outperforming commercial Ir-based catalysts (e.g., Ir/C, IrO_2_ ≥ 311 mV and ≥54.3 mV dec^−1^), gold aerogels, and the Au-Ir NPs in alkaline environment. In addition, a long-term chronopotentiometric test elucidated an excellent stability of the as-prepared Au-Ir aerogel with an overpotential of less than 250 mV after more than 12 h of operation. In contrast, the performance of unsupported Au-Ir NPs rapidly decays within 1 h (Supplementary Fig. 38), which can be ascribed to the detachment of NPs during continuous operation. Moreover, the OER performance was evaluated in acid environment, where Au-Ir aerogels surpassed commercial Ir-based catalysts regarding both the overpotential and stability (Supplementary Fig. 38).

## Discussion

The present work discovers and deeply investigates the unprecedented power and the various roles of the reductants in the gelation of noble metals (as reducing agent, stabilizer, and initiator), unveiling a clear physical picture of the gelation process initiated by excessive NaBH_4_. In light of the impressive power of this method, versatile manipulation of NMAs was unlocked. Gold aerogels with small ligament sizes (as low as 4.8 ± 0.9 nm) and record-high SSAs (as high as 59.8 m^2^ g^−1^) were acquired by resorting to ligand-mediated gelation. The composition space of NMAs was expanded to all common noble metals (Au, Ag, Pd, Pt, Ru, Rh, Ir, Os), where interesting spontaneous combustion phenomena were observed. Moreover, an unconventional ligand-enhancing effect was found to boost the electrocatalytic EOR performance of Au-Pd aerogel by 50%, via loading specific amount of PVP, displaying a forward current density of up to 7.7 times higher than that of commercial Pd/C. In addition, Au-Ir core-shell-structured aerogels were characterized as excellent OER electrocatalysts outperforming commercial Ir/C and IrO_2_ catalysts in both alkaline and acid environment. We believe that the current work not only strides a big step towards understanding the gelation mechanisms, manipulating the microstructures, and enriching the compositions of NMAs, but also opens a new dimension for devising high-performance electrocatalysts by taking advantage of the ligand effects. These findings should adapt to diverse material systems such as hydrogels, aerogels, unsupported porous materials, and nanocrystals.

## Methods

### Fabrication of noble metal aerogels

The synthesis of single metallic gels is described here by taking ligand-free gold gel as an example (*c*_M_ of 0.2 mM is adopted). An aqueous solution of HAuCl_4_·3H_2_O (32.5 mM, 30.8 μL) was added to water (4.87 mL) and stirred for ~1 min, after which freshly prepared NaBH_4_ aqueous solution (1.0 M, 100.0 μL) was rapidly injected and stirred for ~15 s. A monolithic gel formed after grounding for 4–6 h. For the ligand-involved gelation process, all procedures are the same except that the corresponding ligands were added before introducing the metal precursors. For the bimetallic gels, a second metal precursor was introduced, and the molar ratio of the two metal precursors was set to 1/1 while keeping the total metal concentration constant, while no changes were made to the other steps. After acquiring the hydrogels, these products were purified several times with water (total duration of 2–3 days) and then solvent-exchanged with tert-butanol. Finally, the wet gels were flash-frozen by liquid nitrogen, followed by subjecting to freeze drying for 12–24 h to yield the corresponding aerogels.

### Electrochemical tests

A glassy carbon electrode (GCE, 3 mm in diameter) or a rotating disk electrode (RDE, 5 mm in diameter) were used as working electrode, while an Ag/AgCl (saturated KCl aqueous solution) electrode and platinum foil were used as reference and counter electrodes, respectively. EOR tests were performed under N_2_ atmosphere in 1 M KOH aqueous solution containing 1 M ethanol. CV curves were recorded between −0.9 and 0.3 V (vs. AgCl/Ag) with a scanning rate of 50 mV s^-1^, and the stability test was conducted at a potential of −0.23 V (vs. AgCl/Ag). OER tests were performed under N_2_ atmosphere in 1 M KOH or 0.1 M HClO_4_ aqueous solution at 1600 rpm. Linear sweep voltammetry (LSV) curves were measured at room temperature between 1.1 and 1.7 V (vs. RHE) at 10 mV s^−1^, and the stability test was conducted by chronopotentiometry at a current density of ~10 mA cm^−2^.

### Calculation methods

Density functional theory (DFT) calculations were performed with the Gaussian16 package using B3LYP hybrid functional, where the 6−31 + G** basis was adopted to describe anions, while the LANL2DZ and SDD basis sets were applied for gold atoms. Solvent effect imposed by water was described by the polarizable continuum model. The binding energies (*E*_b_) between gold atoms and various anions were defined as *E*_b _= *E*_Au _+ *E*_anion _− *E*_Au-anion_.

## Supplementary information

Supplementary Information

Peer Review

## Data Availability

All data needed to evaluate the conclusions in the paper are present in the paper and/or the Supplementary Materials. Additional data available from authors upon request.
