# Supplementary material for: Unveiling reductant chemistry in fabricating noble metal aerogels for superior oxygen evolution and ethanol oxidation
Source: Nat Commun. 2020 Mar 27;11:1590. doi: 10.1038/s41467-020-15391-w (PMC7101436; doi:10.1038/s41467-020-15391-w)
Supplement: Supplementary file 1 — Supplementary Information [file 41467_2020_15391_MOESM1_ESM.pdf]

Supplementary Information

Unveiling Reductant Chemistry in Fabricating Noble Metal Aerogels for Superior Oxygen Evolution and Ethanol Oxidation

Du et al.

Supplementary Methods

Reagents and Materials

Chemicals including hydrogen tetrachloroaurate (III) ($HAuCl_4 \cdot 3H_2O$), silver nitrate ($AgNO_3$), potassium tetrachloropalladate (II) (K_2PdCl_4), potassium tetrachloroplatinate (II) (K_2PtCl_4), ammonium hexachlororuthenate (IV) ($(NH_4)_2RuCl_6$), ammonium hexachlororhodate (III) ($(NH_4)_3RhCl_6$), ammonium hexachloroosmate (IV) ($(NH_4)_2OsCl_6$), hydrogen hexachloroiridate (IV) hydrate (H_2IrCl_6), trisodium citrate dihydrate (NaCA), sodium borohydride ($NaBH_4$), hydrazine monohydrate (N_2H_4), ascorbate acid (VC), sodium hypophosphite monohydrate (NaH_2PO_2), sodium metaborate tetrahydrate ($NaBO_2$), β-alanine, sodium ascorbate (NaVC), sodium deoxycholate (NaDC), 2-mercaptopropionic acid (MPA), polyvinylpyrrolidone (PVP, M_w = 58000), poly(sodium-4-styrenesulfonate) (PSS, M_w = 70000), cetyltrimethyl ammonium bromide (CTAB), 20% palladium on carbon, and others were purchased from Sigma-Aldrich or Alfa-Aesar. IrO_2 powder and 20% Ir on Vulcan XC-72 (Ir/C) were purchased from Premetek Co. All reagents were used without further purification.

Fabrication of Single-Metallic Hydrogels

All hydrogels were synthesized by either a one-step method or two-step method at ambient temperature (~293 K). For the synthesis of single-metallic gels, the gold system is taken as an example, while all other single metallic gels (*e.g.* Ag, Pd, Pt, Ru, Rh, Os, Ir) were produced by using the respective metal salt precursors following exactly the same one-step method as described below.

One-step method

The fabrication process of a $NaBH_4$-triggered one-step gelation of a gold salt solution is taken as an example. Aqueous solution of $HAuCl_4 \cdot 3H_2O$ (32.5 mM, 30.8 μL) was added in water (4.87 mL) under stirring. Then, freshly prepared $NaBH_4$ aqueous solution (1.0 M, 100.0 μL) was rapidly injected, followed by stirring for ~15 s and grounding for 4−6 h to allow complete reaction. The concentration of the metal precursor solution (c_M) in this case is 0.2 mM, and the molar ratio of the reductant (R) to metal salt (M) was fixed at 100. For the experiments where ligands were included, the aqueous solution of the corresponding ligand (400.0 mM, 25.0 μL) was added before introducing the gold salt.

For scale-up production, an aqueous solution of $HAuCl_4 \cdot 3H_2O$ (32.5 mM, 12.3 mL) was added in 748 mL water and stirred for ~5 min. Then, freshly prepared $NaBH_4$ aqueous solution (1.0 M, 40.0 mL) was rapidly injected in the above mixture, followed by stirring for ~20 s and grounding >12 h.

Two-step method

The two-step method is divided into two processes, *i.e.* nanoparticle (NP) preparation and hydrogel formation. The process of $NaBH_4$-triggered gelation of PVP-stabilized gold NP solutions is taken as an example.

Preparation of Gold NP Solutions

Aqueous solutions of PVP (400.0 mM, 25.0 μL) and $HAuCl_4 \cdot 3H_2O$ (32.5 mM, 30.8 μL) were added successively in water (4.82 mL) and stirred for ~10 min. Then, freshly prepared $NaBH_4$ aqueous solution (200.0 mM, 20.0 μL) was rapidly injected, followed by stirring for *ca.* 2 min. The molar ratio of the metal salt, ligand (L), and reductant was fixed at 1/10/4. The as-prepared NP solution was subjected to further gelation experiments within 15 min.

Preparation of Gold Hydrogels

Aqueous solution of $NaBH_4$ (1 M, 100.0 μL) was added to the as-prepared gold NP solution, followed by stirring for 10–20 s and grounding for 6–12 h to acquire a gold hydrogel.

Fabrication of Bimetallic Hydrogels

Bimetallic hydrogels were synthesized by a one-step approach. The procedure is the same as that of the single-metallic hydrogels, except that two metal precursor salts were introduced. The molar ratio of the different metal precursor salts was set to 1:1 for the bi-metallic systems, and the total concentration of the metal salts in the final solution, if not specified, was fixed at 0.5 mM.

Fabrication of Metal Aerogels

The as-prepared hydrogels were washed by a large amount of water for 4–5 times with a total duration of 2–3 days, then subjected to tert-butanol for solvent-exchange and further purification for 2 times at 303 K. For PVP-involved synthesis, an additional washing step by using ethanol was applied before the solvent-exchange process. Afterwards, the wet gels were flash-freezed by liquid nitrogen and remained at −196 ºC for ~10 min to enable complete freezing. The frozen samples were put into the chamber of a freeze drier (TOPTI-12S-80) and dried for 12–24 h at ~1 Pa. The temperature of the cold trap was set to −80 ºC. The yield of NMAs was calculated based on the mass of the final aerogels with reference to the mass of the corresponding precursor metal salts.

Fabrication of Bimetallic NPs

Concentrated Au-Pd and Au-Ir bimetallic NP solutions were produced for comparing the electrocatalytic performance with that of the corresponding aerogels. Au-Pd and Au-Ir NP solutions were prepared by a similar procedure. Taking the preparation of the Au-Pd NP solution as an example, aqueous solutions of $HAuCl_4 \cdot 3H_2O$ (32.5 mM, 3.075 mL) and K_2PdCl_4 (32.5 mM, 3.075 mL) were added in water (374 mL) under stirring. Then, freshly prepared $NaBH_4$ aqueous solution (1.0 M, 20 mL) was rapidly injected, followed by stirring for 30–60 min. Afterwards, the solution was concentrated by ultracentrifugation, where the volume was reduced from ~400 mL to ~5 mL. The resulting solutions were further purified by dilution with deionized water and concentrated again for several times.

Computational Procedures

Density-functional theory Calculations

Density-functional theory (DFT) calculations were performed with the Gaussian16 package[1] using the B3LYP hybrid functional. The 6-31+G** basis was adopted to describe anions, such as F^-, BH_4^-, citrate^{3-}, *etc*. For the gold atoms, the commonly used LANL2DZ and SDD basis sets were applied.[2] Both geometric optimization and self-consistent energy calculations were carried out at the same computation level. The solvent effect imposed by water was described by the polarizable continuum model.[3] The binding energies (E_b) between one gold atom and various anions were defined as $E_b = E_{Au} + E_{anion} - E_{Au\text{-}anion}$ on the basis of the following process:

$$\text{Au–anion} \rightarrow \text{Au} + \text{anion} \qquad \text{(Supplementary Equation 1)}$$

The E_b between two gold atoms and various anions is $E_b = E_{2Au} + E_{anion} - E_{2Au\text{-}anion}$ following the reaction:

$$\text{Au–Au–anion} \rightarrow \text{Au–Au} + \text{anion} \qquad \text{(Supplementary Equation 2)}$$

The reaction energies accounting for the fusion anion-stabilized gold NPs were calculated on the basis of the following process:

$$\text{2 Au-anion} \rightarrow \text{Au-Au} + \text{2 anions} \qquad \text{(Supplementary Equation 3)}$$

Characterizations

Microscopy Characterization

Scanning electron microscopy (SEM) analysis was performed on a Zeiss Gemini 500 scanning electron microscope. Samples were prepared by directly sticking on conductive tape.

Transmission electron microscopy (TEM) analysis was carried out by using a FEI Tecnai G2 20 microscope operated at 200 kV. Samples were prepared by dispersing in acetone under ultrasonication (15 s to 120 s, depending on their dispersing ability), followed by dropping onto carbon-coated copper grids and drying at ambient temperature.

High-angle annular dark-field scanning transmission electron microscopy (HAADF-STEM) imaging and spectrum imaging based on energy-dispersive X-ray spectroscopy (EDX) were performed at 200 kV with a Talos F200X microscope equipped with an X-FEG electron source and a Super-X EDX detector system (FEI). Prior to STEM analysis, the specimen mounted in a high-visibility low-background holder was placed for 2 s into a Model 1020 Plasma Cleaner (Fischione) to remove possible contamination.

Optical imaging was performed on a Carl Zeiss Microscopy, with the magnification of 63×10.

Diffraction Characterization

X-ray powder diffraction (XRD) was carried out in reflection mode on a Siemens D5000 X-ray diffractometer operated at a voltage of 30 kV and a current of 10 mA with Cu Kα radiation ($\lambda = 1.5406$ Å). The data were collected in the range of 20°–90° (2θ) with a step size of $\Delta 2\theta = 0.02°$. The sample was fixed on the holder by Scotch tape. For the single-metallic systems, the crystallite size was estimated by the Scherrer equation applying a crystallite-shape factor $K = 0.9$.

Spectroscopy Characterization

Fourier-transform infrared spectroscopy (FT-IR) spectra were recorded on a Thermo Scientific Nicolet 8700 FT-IR Spectrometer configured with a Smart iTR diamond accessory.

Ultraviolet–visible spectroscopy (UV-vis) absorption spectra were recorded on a Cary 60 UV-Vis Spectrophotometer.

Thermal Properties Characterization

Thermogravimetric analysis (TGA) was conducted by a Diamond TG-DTA/Spectrum GX system with a heating rate of 10 K min^{-1} under nitrogen atmosphere.

Element Analysis

X-ray photoelectron spectroscopy (XPS) was performed on an Axis Ultra spectrometer (Kratos, UK) with a high-performance Al monochromatic source operated at 15 kV. The XPS spectra were taken after all binding energies were referenced to the C 1s neutral carbon peak at 284.8 eV, and the elemental compositions were determined from the peak area ratios after correction for the sensitivity factor by CasaXPS.

Inductively coupled plasma optical emission spectroscopy (ICP-OES) was performed on a Perkin-Elmer Optima 7000DV optical emission spectrometer.

Gas Adsorption Measurements

Nitrogen adsorption experiments were performed with a Quantachrome NOVA 3000e system at 77 K. The sample was outgassed at 323 K for ~24 h under vacuum before measurement. The filling rod was used to reduce the dead volume, thus improving the measurement accuracy. The specific surface area was calculated by using the multi-point BET equation ($0.1 < p/p_0 < 0.3$). The pore size distribution was derived by using the non-local density functional theory (NLDFT) method (N_2 at 77 K on carbon) based on a slit pore geometry. The total pore volume was calculated at $p/p_0 = 0.99$, similar to the value derived by the BJH (Barret–Joyner–Halender) method. For low-surface-area samples, instead of nitrogen, krypton (Kr) was used as the probe molecule to quantify the specific surface area, due to their lower saturation pressure (≈2.63 Torr for supercooled liquid krypton at 77 K) compared to that of nitrogen molecules (≈760 Torr at 77 K), thus allowing to acquire a much more accurate result ascribed to a large relative pressure change with a small amount of gas adsorption/desorption.

Other Characterizations

Zeta potential tests were performed on ZETASIZER NANO (ZEN5600, Malvern Company). The original solutions of gold nanoparticles and salts were purified separately with a 0.45 μm filter membrane before the tests. To reflect the real reaction conditions and to avoid the possible deviations incurred by dilution, the concentrations of all reactants (metal precursors, ligands, and salts) used here were exactly the same as described in the experimental part.

Electrochemical Measurements

All electrochemical tests were performed with a three-electrode system on an Autolab/PGSTAT 30 (Eco Chemie B. V. Utrecht, the Netherlands) or CHI potentiostat (CHI 760D). A glassy carbon electrode (GCE, 3 mm in diameter) or a rotation disk electrode (RDE, 5 mm in diameter), an Ag/AgCl (saturated KCl aqueous solution) electrode, and a platinum foil were used as working electrode, reference electrode, and counter electrode, respectively.

For modification of the working electrode for ethanol oxidation reaction (EOR) tests, ~1 mg catalyst was dispersed in 2-propanol (IPA, 425 μL) and Nafion solution (1 wt.% in IPA, 75 μL) by sonicating for ca. 30 min to acquire the catalyst ink. For certain experiments, a specific amount of PVP (in IPA solution) was added before sonication. For oxygen evolution reaction (OER) tests, catalyst inks were prepared by dispersing ~2 mg catalysts in IPA (1650 μL) and Nafion (350 μL, 1 wt.% in IPA) solutions. Then, a specific amount of ink was transferred onto the working electrode and left evaporating at ambient temperature. Then, additional Nafion solution (0.2 wt.% in IPA, 4 μL) was coated on the working electrode. The final loading of Pd, Ir, or Ru in ink was set to ~20 μg cm^{-2}.

EOR tests were performed under N_2 atmosphere in 1 M KOH aqueous solution containing 1 M ethanol. CV curves were recorded between -0.9 and 0.3 V (*vs.* AgCl/Ag) with a scanning rate of 50 mV s^{-1}. The stability test was conducted at a potential of -0.23 V (*vs.* AgCl/Ag). To compare the morphology before and after cycling, the gold foil was used as the working electrode to facilitate characterization. OER tests were performed on RDE under N_2 atmosphere in 1 M KOH or 0.1 M $HClO_4$ aqueous solution at a rotating speed of 1600 rpm. All potentials were converted to RHE. The linear sweep voltammetry (LSV) curves were measured at room temperature between 1.1−1.7 V (*vs.* RHE) at 10 mV s^{-1} with iR compensation as reported elsewhere.[4] The stability test was performed by chronopotentiometry at a current density of ~10 mA cm^{-2} if not specified.

Supplementary Figures

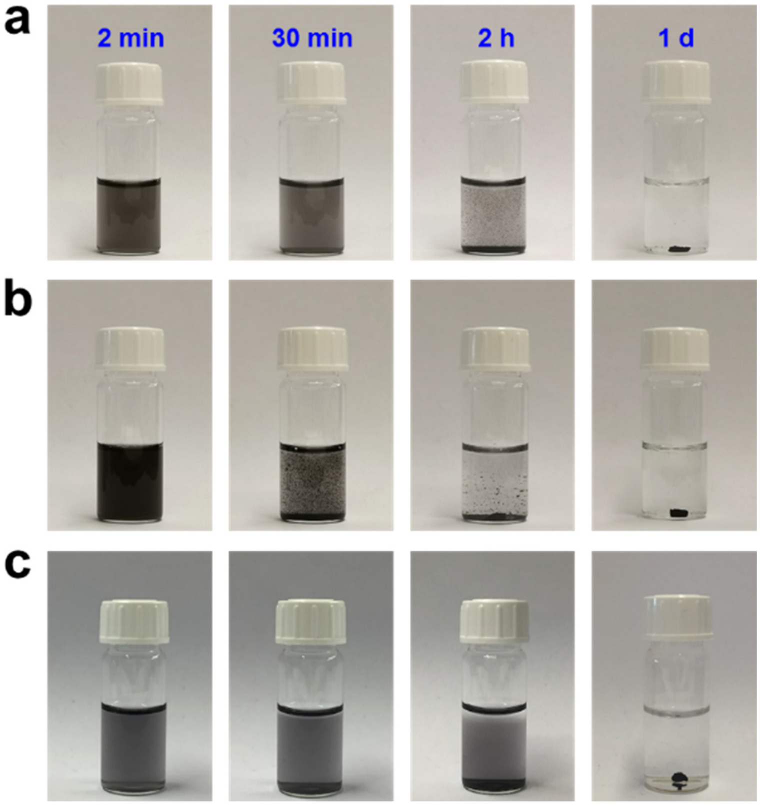

Supplementary Figure 1 | Gold gels fabricated by different methods. Time-lapse photographs of the fabrication of gold gels initiated by (a) $NaBH_4$ (c_M = 0.2 mM, R/M = 500), (b) $NaBH_4$ (c_M = 0.5 mM, R/M = 500), and (c) NH_4F (c_M = 0.2 mM).

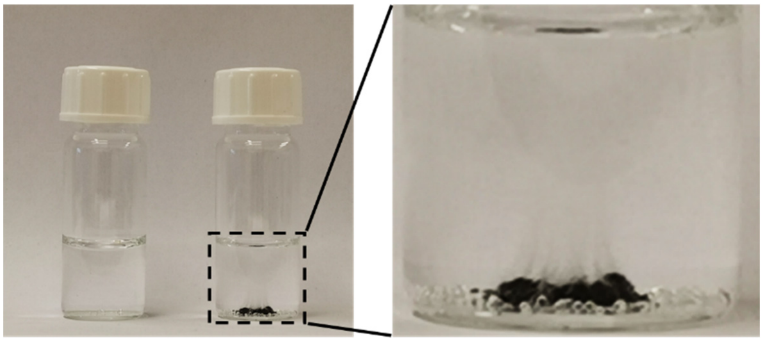

Supplementary Figure 2 | Catalytic decomposition of $NaBH_4$ by gold aerogels. The decomposition of $NaBH_4$ aqueous solution (0.2 M) without and with gold aerogels, and the corresponding magnified photograph.

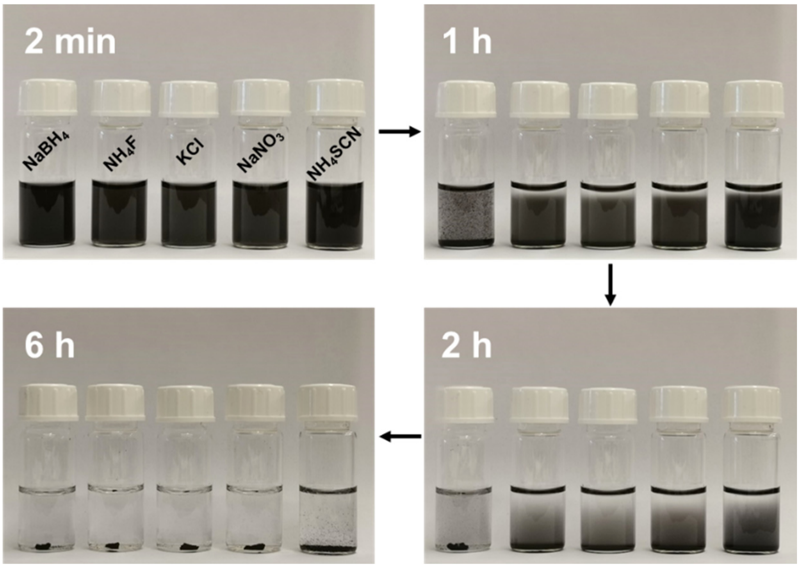

Supplementary Figure 3 | Comparison of various gelation methods. Comparison of the gelation speed of gold systems (c_M = 0.5 mM) initiated by different methods.

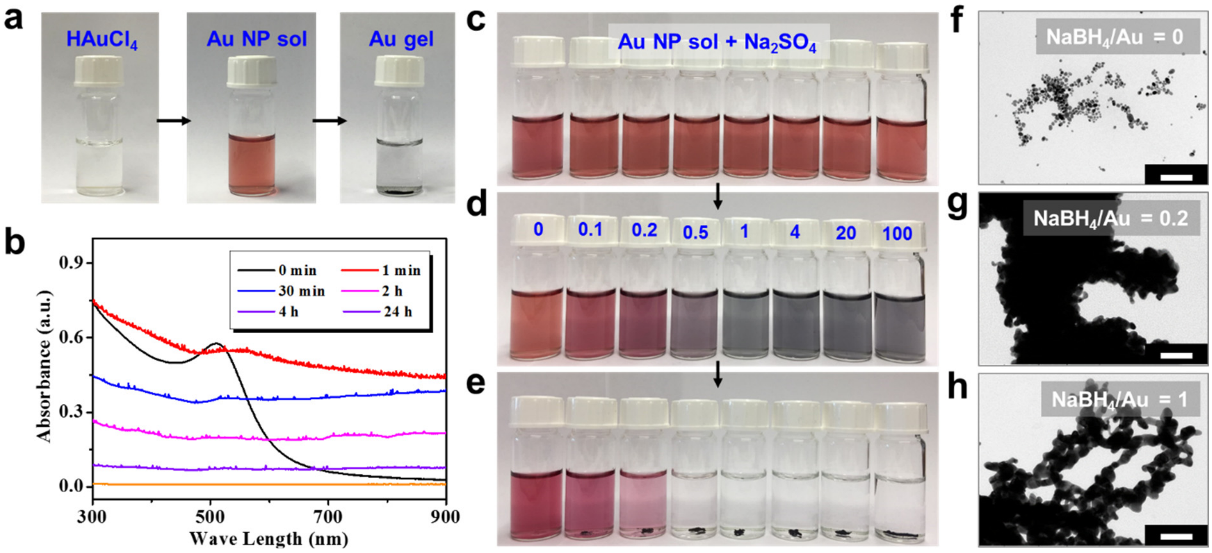

Supplementary Figure 4 | $NaBH_4$-triggered fabrication of gold gels by the two-step method. (a) Transformation of $HAuCl_4$ aqueous solution to gold NP solution (c_M = 0.2 mM, $NaBH_4$/Au = 4/1) and further to a gold gel ($NaBH_4$/Au = 100/1), and (b) the corresponding time-lapse UV-vis absorption spectra (0 min refers to the gold NP solution without addition of an excessive amount of $NaBH_4$). (c-e) Photographs of the $NaBH_4$-assisted gelation in the presence of Na_2SO_4. The Au NP solutions (c_M = 0.2 mM aged for 3 days) were firstly processed with Na_2SO_4 (33 mM), then different amounts of $NaBH_4$ ($NaBH_4$/Au range from 0 to 100) were added to assist gelation. (f-h) TEM images of the as-obtained gels with $NaBH_4$/Au ratios of 0, 0.2, and 1. Scale bars in figures f-h are 200 nm.

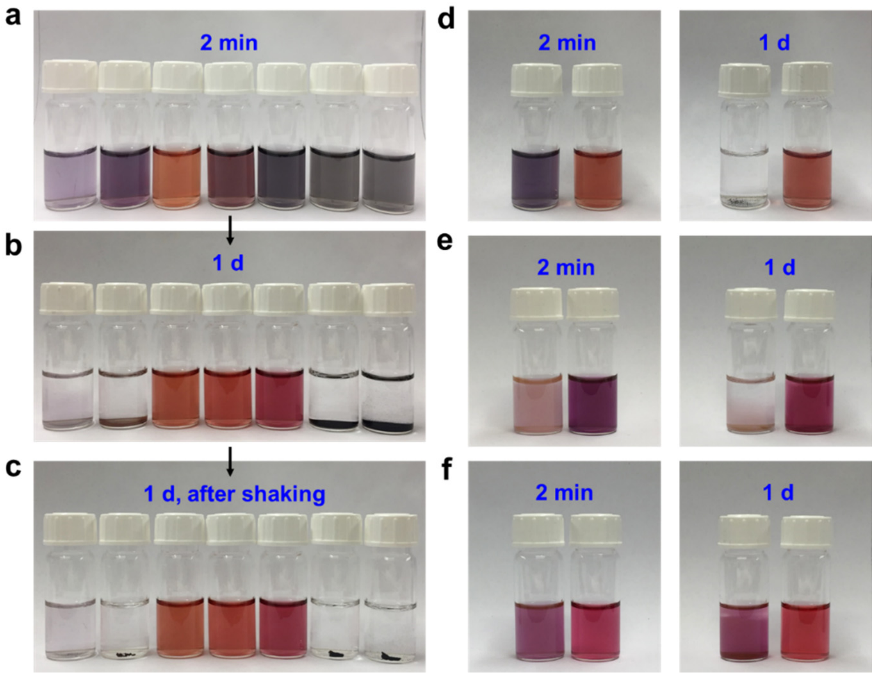

Supplementary Figure 5 | Exploration of the roles played by the reductant. (a-c) From left to right, $NaBH_4$ of 0.5, 1.5, 4, 10, 20, 50, 100 equivalents (compared with gold) were added in $HAuCl_4$ aqueous solutions (c_M = 0.2 mM). (d-f) $HAuCl_4$ aqueous solutions (c_M = 0.2 mM) without (left vials) and with (right vials) the presence of 10 equivalents NaCA after 2 minutes and 1 day upon addition of 1.0 equivalent (d) $NaBH_4$, (e) N_2H_4, and (f) NaVC.

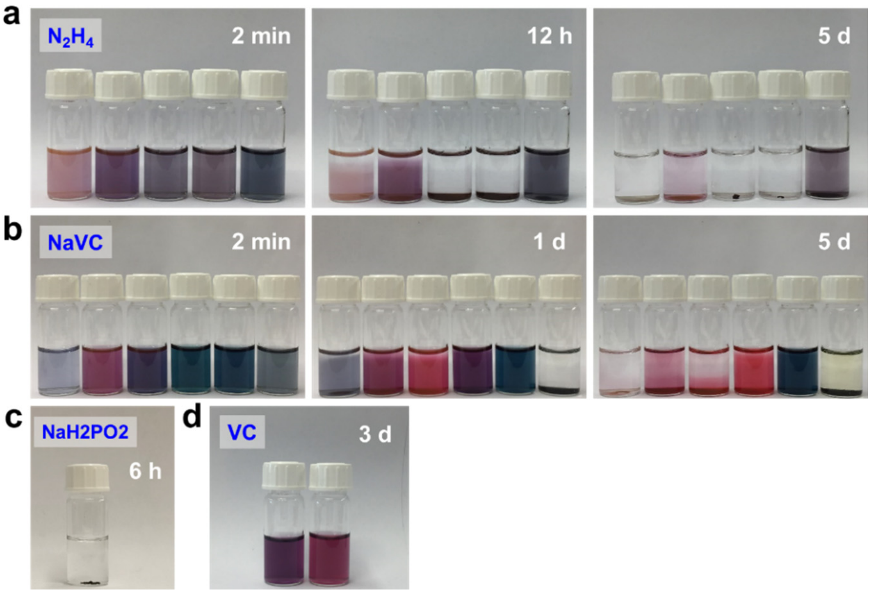

Supplementary Figure 6 | Destabilization of $HAuCl_4$ aqueous solutions by various reductants (c_M = 0.2 mM). Time-lapse photographs of (a) N_2H_4- (1, 4, 20, 100, 500 equivalents) and (b) NaVC- (0.5, 1, 4, 20, 100, 500) triggered destabilization of $HAuCl_4$ aqueous solutions. Destabilization triggered by (c) NaH_2PO_2 at 343 K, R/M = 100 and by (d) VC, R/M = 50 (left) and 200 (right).

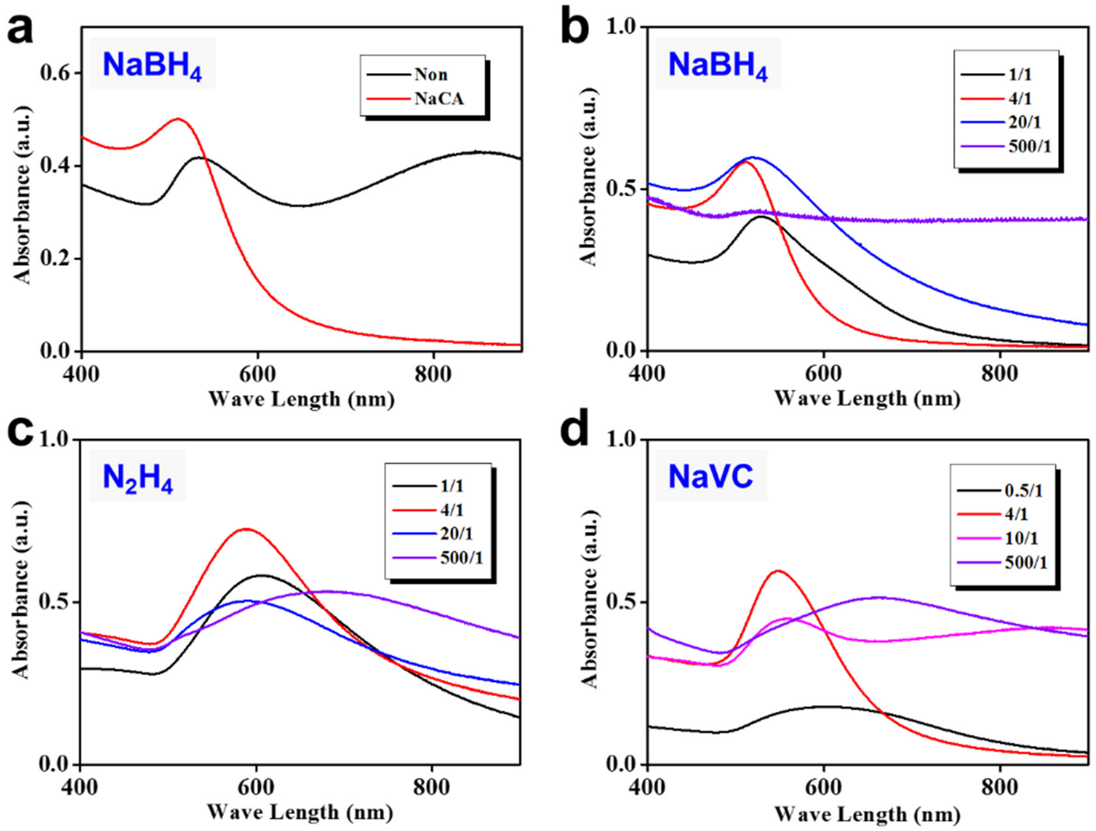

Supplementary Figure 7 | UV-vis absorption spectra of $HAuCl_4$ aqueous solutions upon addition of reductants (c_M = 0.2 mM). (a) Addition of 1 equivalent $NaBH_4$ in $HAuCl_4$ aqueous solutions with or without the presence of NaCA. Addition of (b) $NaBH_4$, (c) N_2H_4, and (d) NaVC in $HAuCl_4$ aqueous solutions at different R/M ratios.

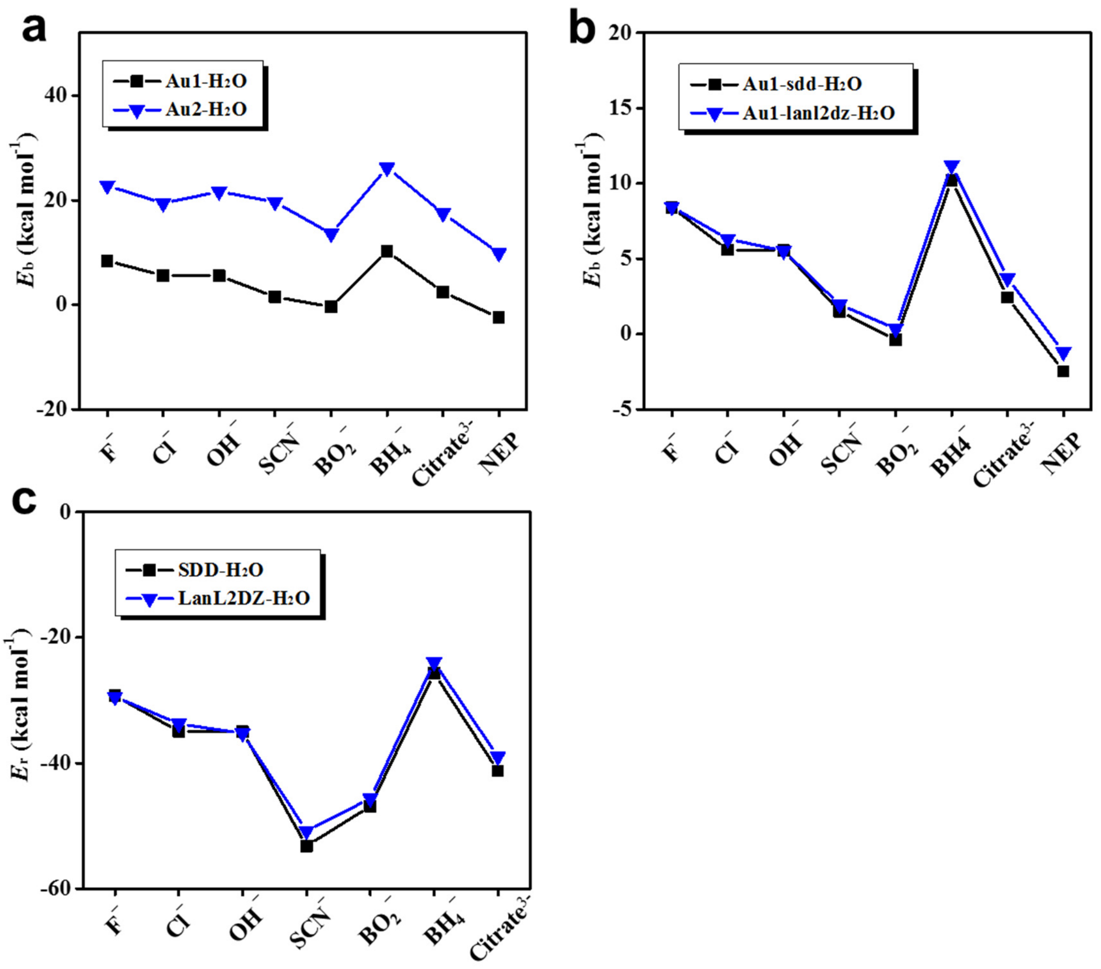

Supplementary Figure 8 | DFT calculations. (a) Binding energies of anions/molecules with one (black line) and two (blue line) gold atoms in water by using the SDD method. An increase in E_b with increasing number of gold atoms is observed, while the trend against various anions/molecules is similar. This suggests that our analysis should be general and could be extended to the interactions between Au nanoparticles and anions. (b) Comparison of the binding energies of anions/molecules with one gold atom in water by using the SDD and LanL2DZ method, where both methods give similar results. (c) The reaction energies (E_r) of the fusion of gold NPs stabilized by various anions. NEP represents N-ethylpyrrolidone, which was used as a representative unit of PVP.

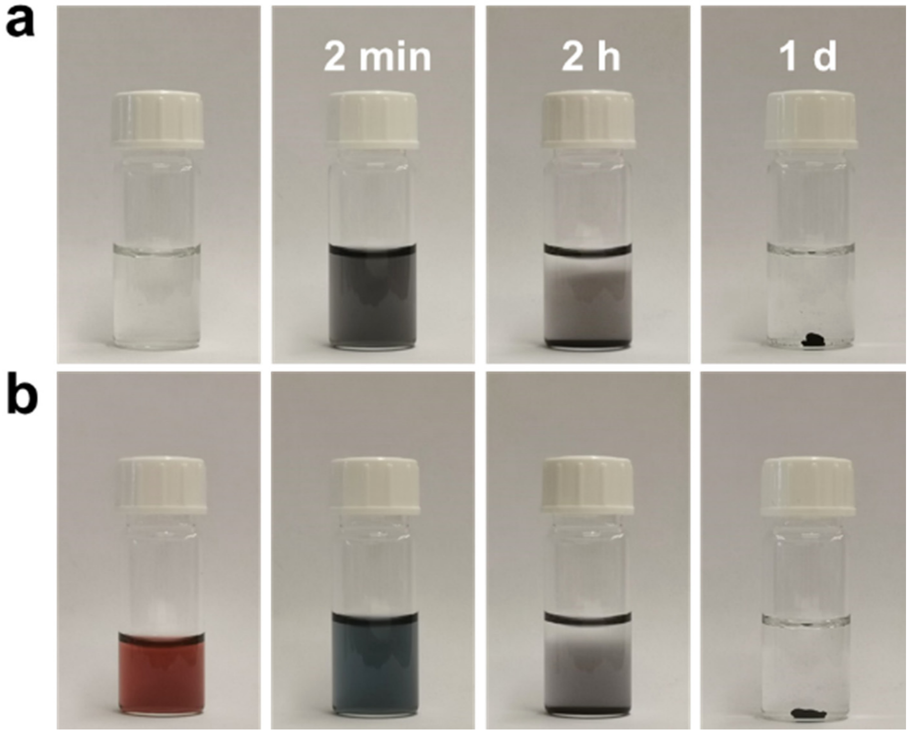

Supplementary Figure 9 | Gelation induced by the ligand. Gelation was initiated by addition of (a) 4 equivalents $NaBH_4$ in $HAuCl_4$ solution with the existence of NaCA, and by addition of (b) NaCA in a gold NP solution. The NaCA/gold ratio was fixed at 100 in both cases.

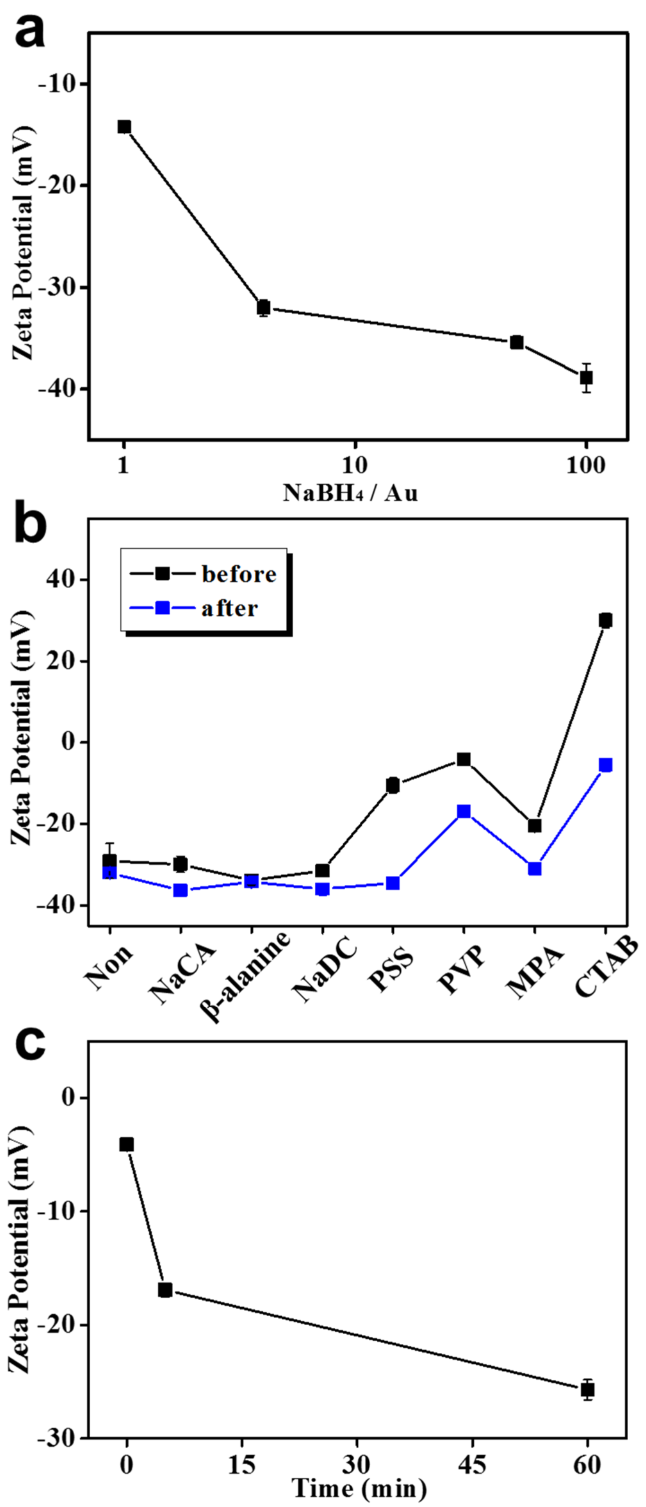

Supplementary Figure 10 | Zeta potential of various gold NP solutions (c_M = 0.2 mM). (a) Zeta potential of freshly prepared gold solutions after reducing $HAuCl_4$ by adding various amounts of $NaBH_4$. (b) Zeta potential of various ligand-stabilized gold NP solutions before (black line) and after (blue line) destabilization by $NaBH_4$ for *ca.* 5 min. (c) Time-lapse zeta potential evolution of PVP-coordinated gold NP solutions as destabilized by $NaBH_4$.

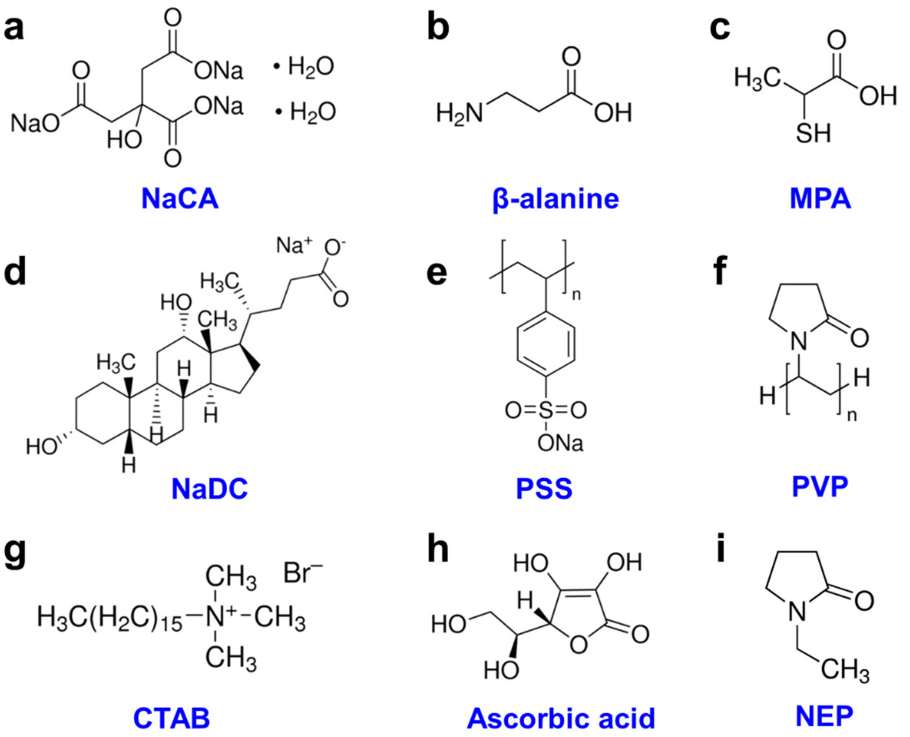

Supplementary Figure 11 | Molecular structures of some chemicals used in this study.

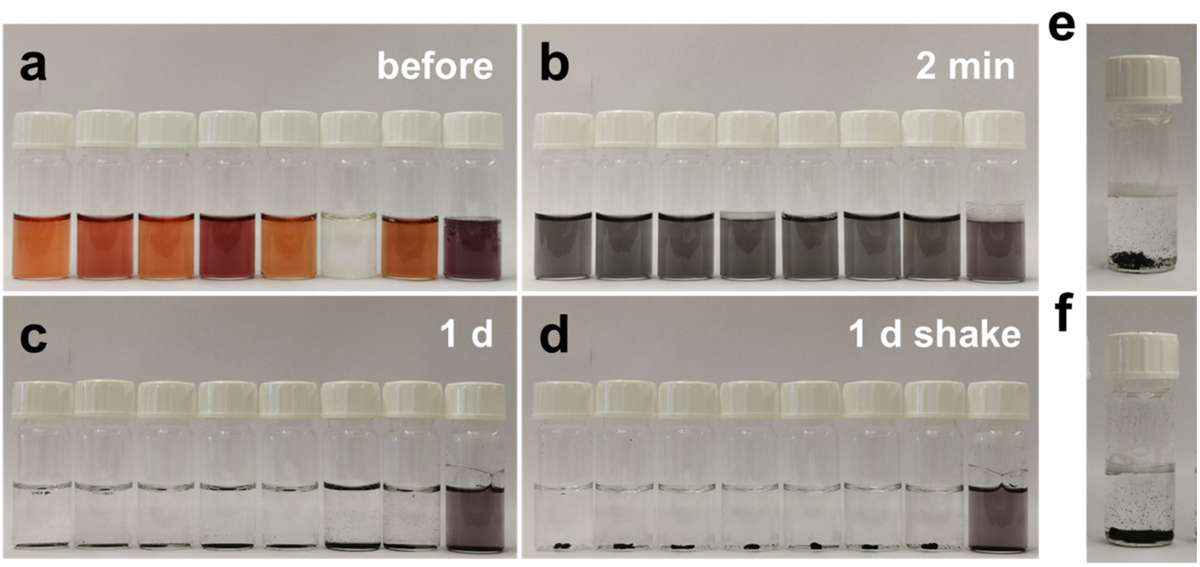

Supplementary Figure 12 | Destabilization of diverse ligand-stabilized gold NP solutions. (a-d) From left to right, destabilization of gold NP solutions (c_M = 0.2 mM) which were stabilized without ligand and with NaCA, β-alanine, NaDC, PSS, MPA, PVP, and CTAB (ligand/gold ratio was fixed at 10/1), respectively. (a) Before and after addition of $NaBH_4$ for (b) 2 min, (c) 1 day, and (d) 1 day after shaking to facilitate observation. (e-f) Destabilization of CTAB-stabilized gold NP solutions: (e) c_M = 0.2 mM, CTAB/gold=2/1, (f) c_M = 0.5 mM, CTAB/gold=2/1.

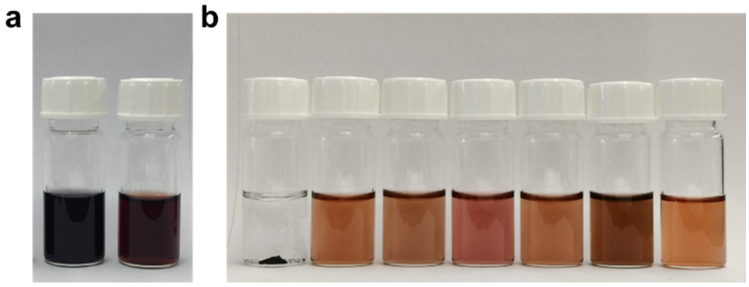

Supplementary Figure 13 | Gelation of gold NP solutions in the presence of ligands. (a) Gold NP solutions (c_M = 1 mM) stabilized by NaDC (left vial) and PVP (right vial) after 6 days. (b) Destabilization of PVP-stabilized gold NP solutions (c_M = 0.2 mM) by different approaches after 1 day, from left to right: $NaBH_4$, incubation at 343 K, H_2O_2 (100 mM), NH_4F (100 mM), $CaCl_2$ (1 mM), dopamine (1 mM), and ethanol (10 vol.%).

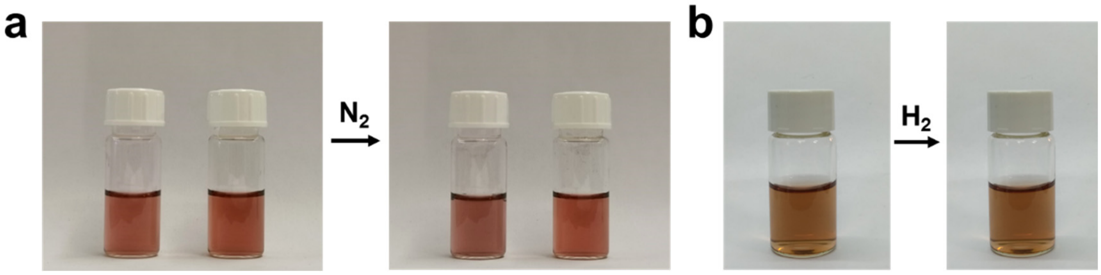

Supplementary Figure 14 | Treatment of PVP-stabilized gold NP solutions with the assistance of bubbling. (a) Before (left photograph) and after (right photograph) bubbling with N_2 for 15 minutes followed by grounding for 1 day, in the presence of 100 mM NH_4F (left vial) and NaCl (right vial). (b) $HAuCl_4$/PVP/$NaBO_2$ (1/10/100) NP solutions before (left) and after (right vial) bubbling with H_2 for 15 minutes followed by grounding for 1 day.

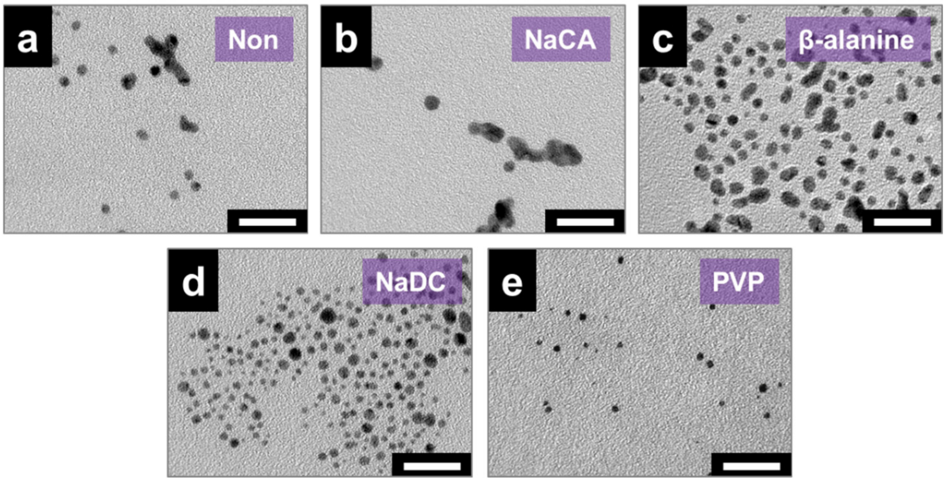

Supplementary Figure 15 | Representative TEM images of gold NPs fabricated in the presence of different ligands. (a) Without ligand and with (b) NaCA, (c) β-alanine, (d) NaDC, and (e) PVP. Scale bars for all figures are 20 nm.

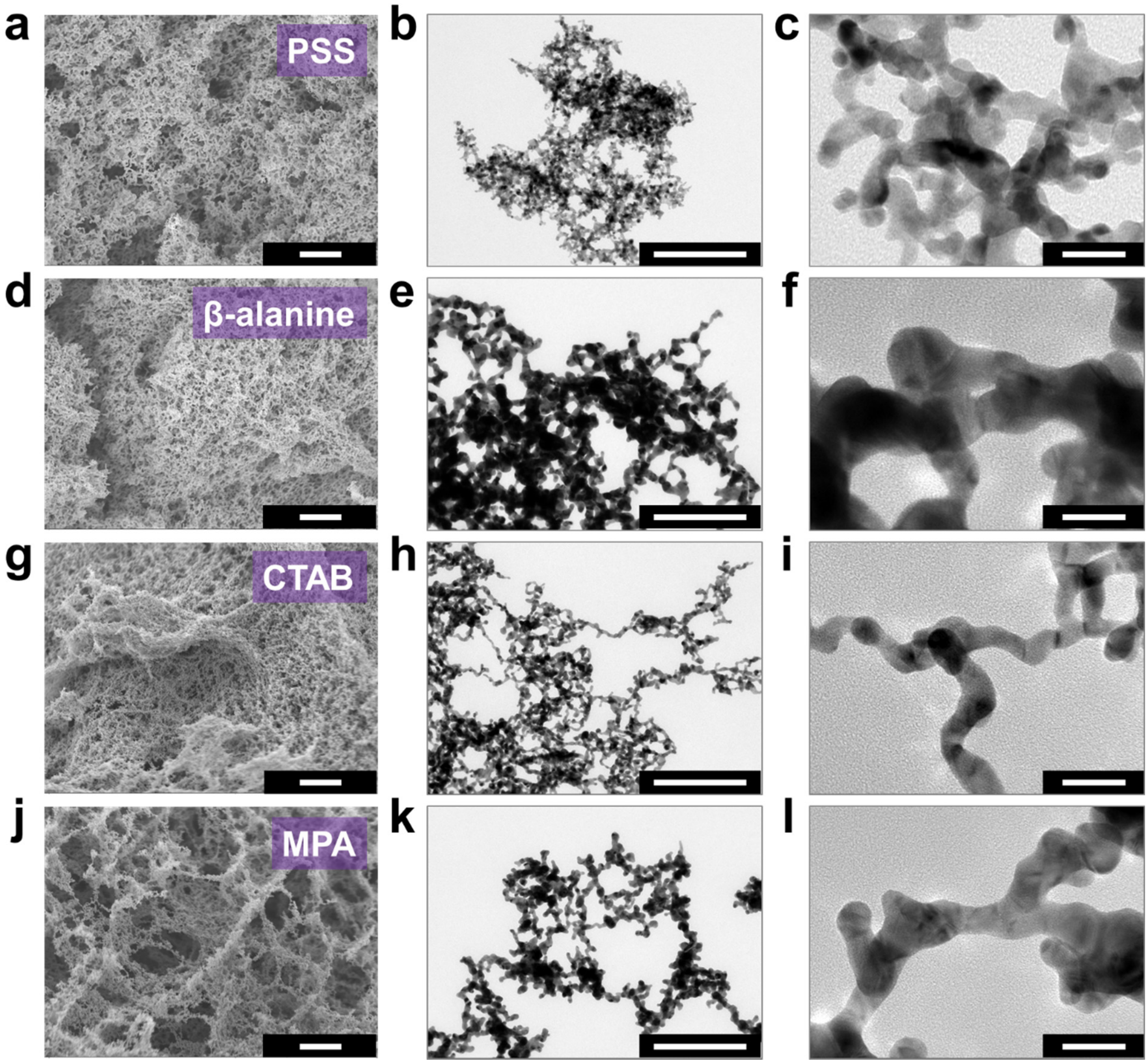

Supplementary Figure 16 | SEM and TEM images of gold gels fabricated from various gold NP solutions. Gold NPs are stabilized by (a-c) PSS, (d-f) β-alanine, (g-i) CTAB, and (j-l) MPA. c_M = 0.2 mM for d-f, and c_M = 0.5 mM for the others. Scale bars in figures a, d, g, j are 1 μm, in b, e, h, k are 200 nm, and in c, f, i, l are 20 nm.

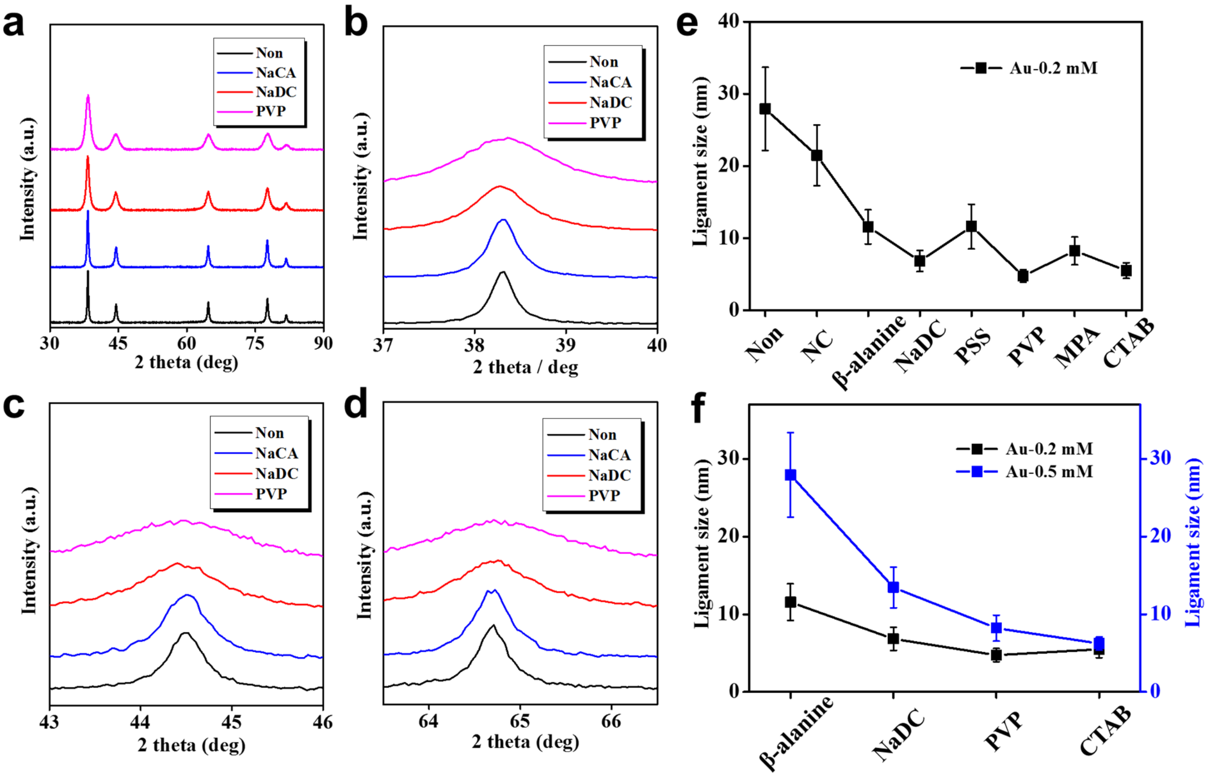

Supplementary Figure 17 | Estimation of the feature sizes of the gold gels prepared from various ligand-stabilized solutions. (a-d) For XRD tests, the full width at half maximum (FWHM) can reflect the crystallite size of the corresponding materials (calculated to be 25.2, 19.3, 9.6, and 4.8 nm for ligand-free, NaCA, NaDC, and PVP samples, respectively). (e-f) TEM observations show the ligament size versus ligand species used in the fabrication process with c_M = 0.2 mM or 0.5 mM (error bars depict the standard deviation).

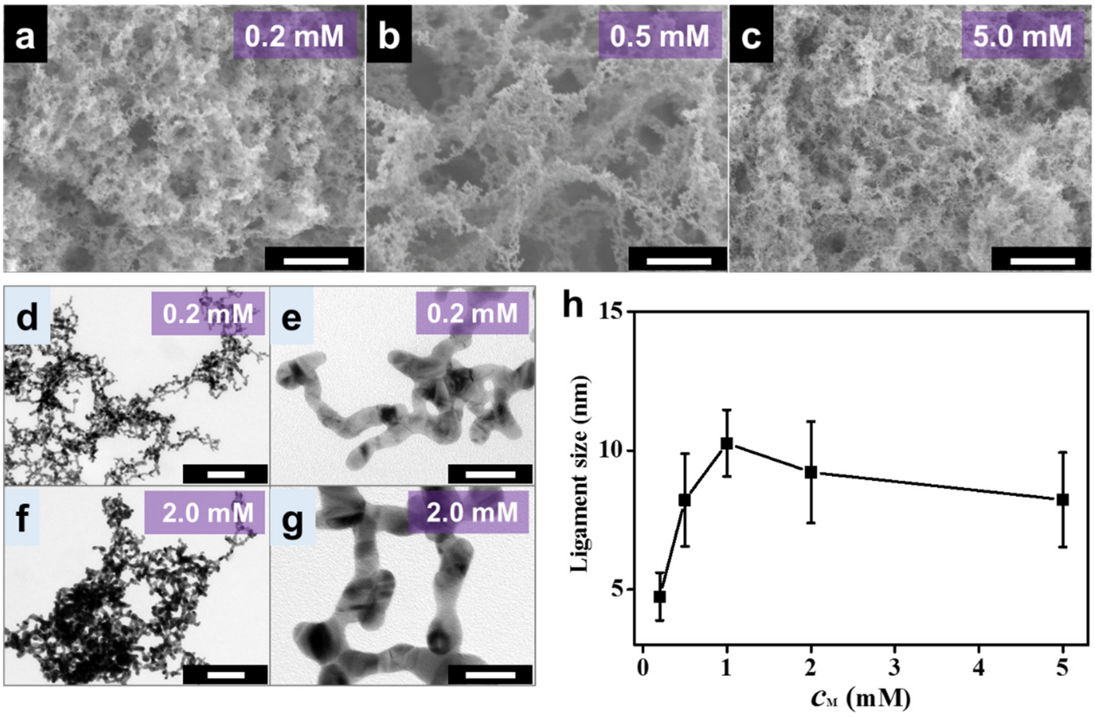

Supplementary Figure 18. Characterization of gels prepared from PVP-stabilized gold NP solutions of indicated c_M. (a-c) SEM images and (d-g) TEM images. (h) The plotting of c_M versus ligament size of these aerogels (error bars depict the standard deviation). Scale bars in figures a-c are 500 nm, in d, f are 100 nm, and in e, g are 20 nm.

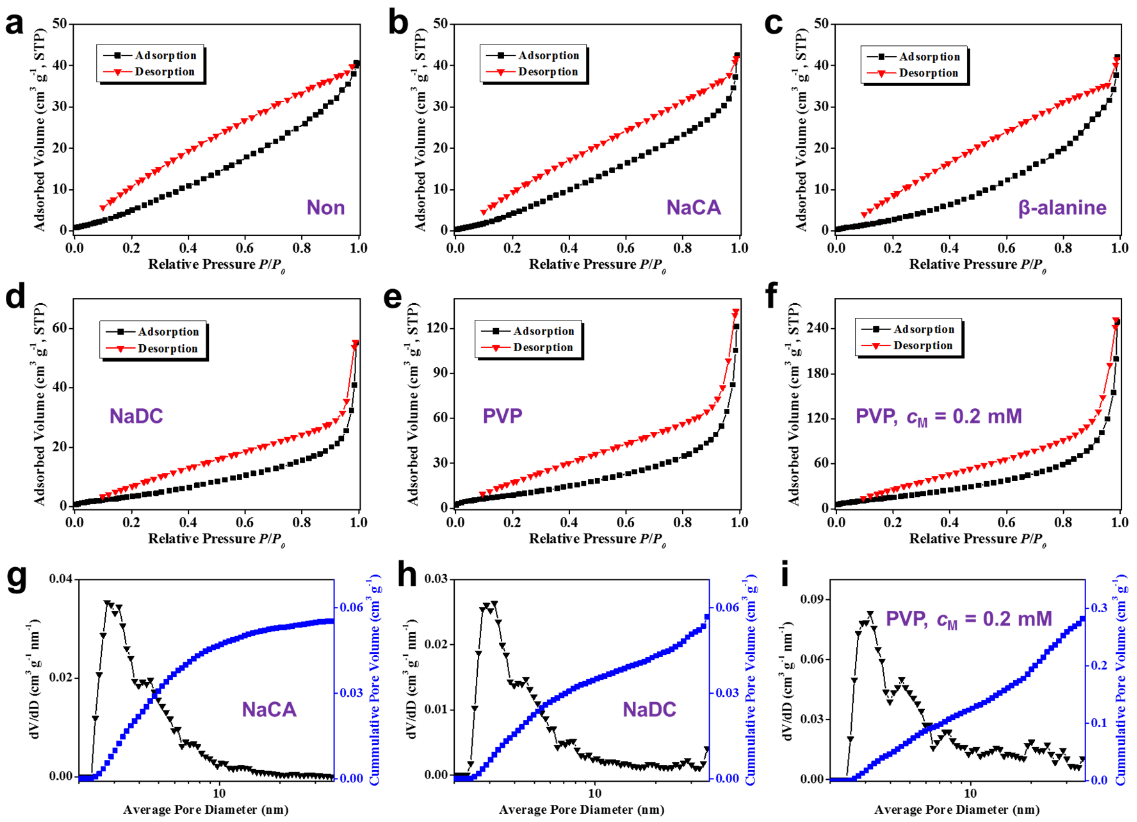

Supplementary Figure 19. Nitrogen adsorption tests of gold aerogels fabricated with different ligands. Adsorption/desorption isotherms of gold aerogels prepared with (a) no ligand, (b) NaCA, (c) β-alanine, (d) NaDC, and (e-f) PVP. Pore size distribution of gold aerogels prepared with (g) NaCA, (h) NaDC, and (i) PVP. c_M = 0.5 mM for a-e, g, and h, while c_M = 0.2 mM for f and i.

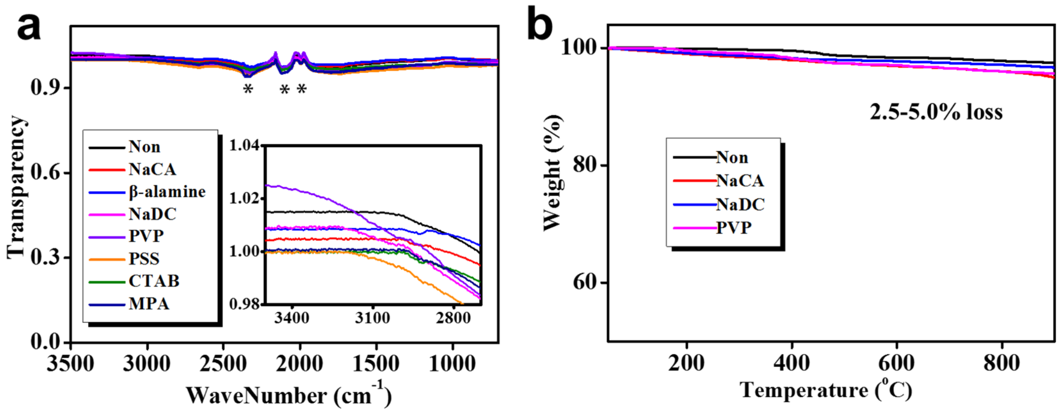

Supplementary Figure 20. Characterization of the impurities in the aerogels. (a) IR spectra and (b) TGA tests of gold aerogels fabricated from gold NP solutions stabilized by various ligands.

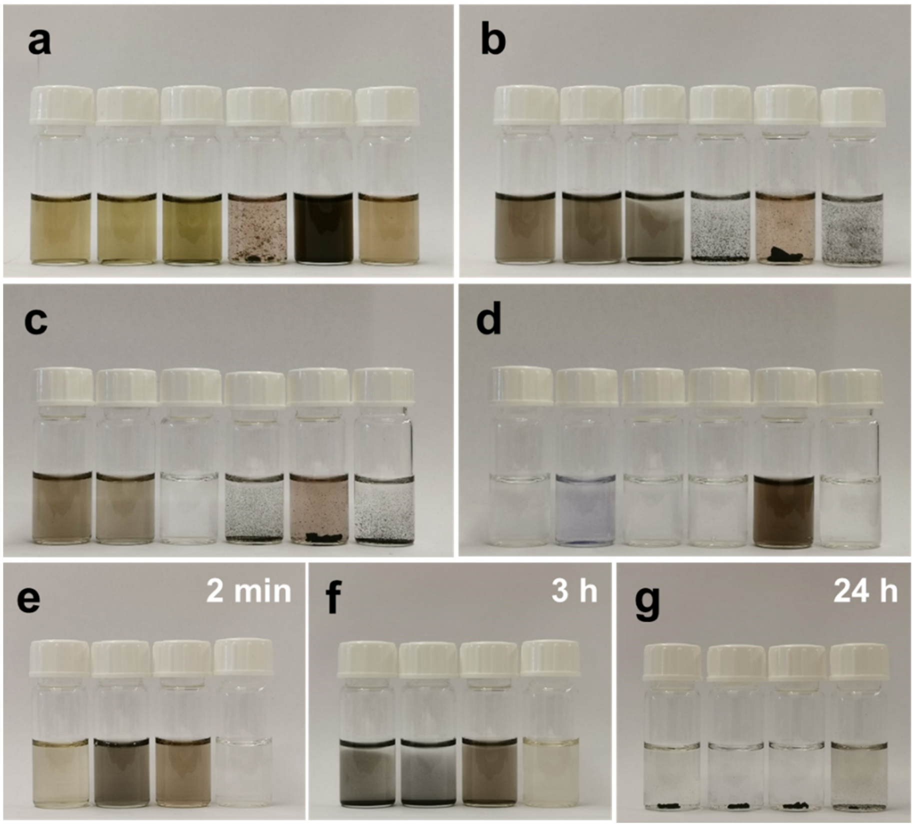

Supplementary Figure 21. Gelation of different noble metal systems (c_M = 0.2 mM, stabilized by NaCA). Destabilization of (a) Ru, (b) Rh, (c) Os, and (d) Ir systems after 1 day, from left to right: original NP solution, 343 K incubation, H_2O_2 (100 mM), $CaCl_2$ (1 mM), dopamine (1 mM), and NH_4F (100 mM). (e-g) Destabilization of Ru, Rh, Os, and Ir NP solutions by using $NaBH_4$ with prolonged time.

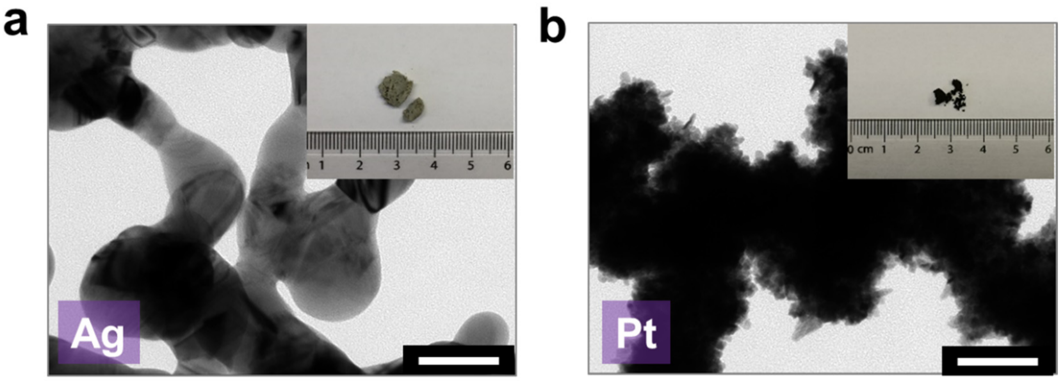

Supplementary Figure 22 | Silver and platinum aerogels. TEM images and photographs (inset) of (a) silver and (b) platinum aerogels fabricated by excessive-$NaBH_4$–directed gelation. Scale bars are 50 nm for all figures.

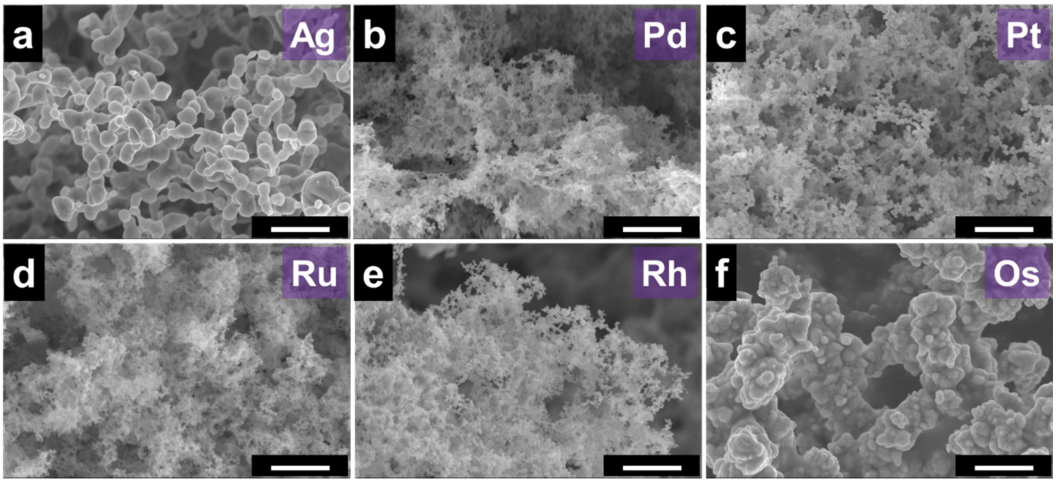

Supplementary Figure 23 | SEM images of single-metallic aerogels. (a) Ag, (b) Pd, (c) Pt, (d) Ru, (e) Rh, and (f) Os. Scale bars are 500 nm for all figures.

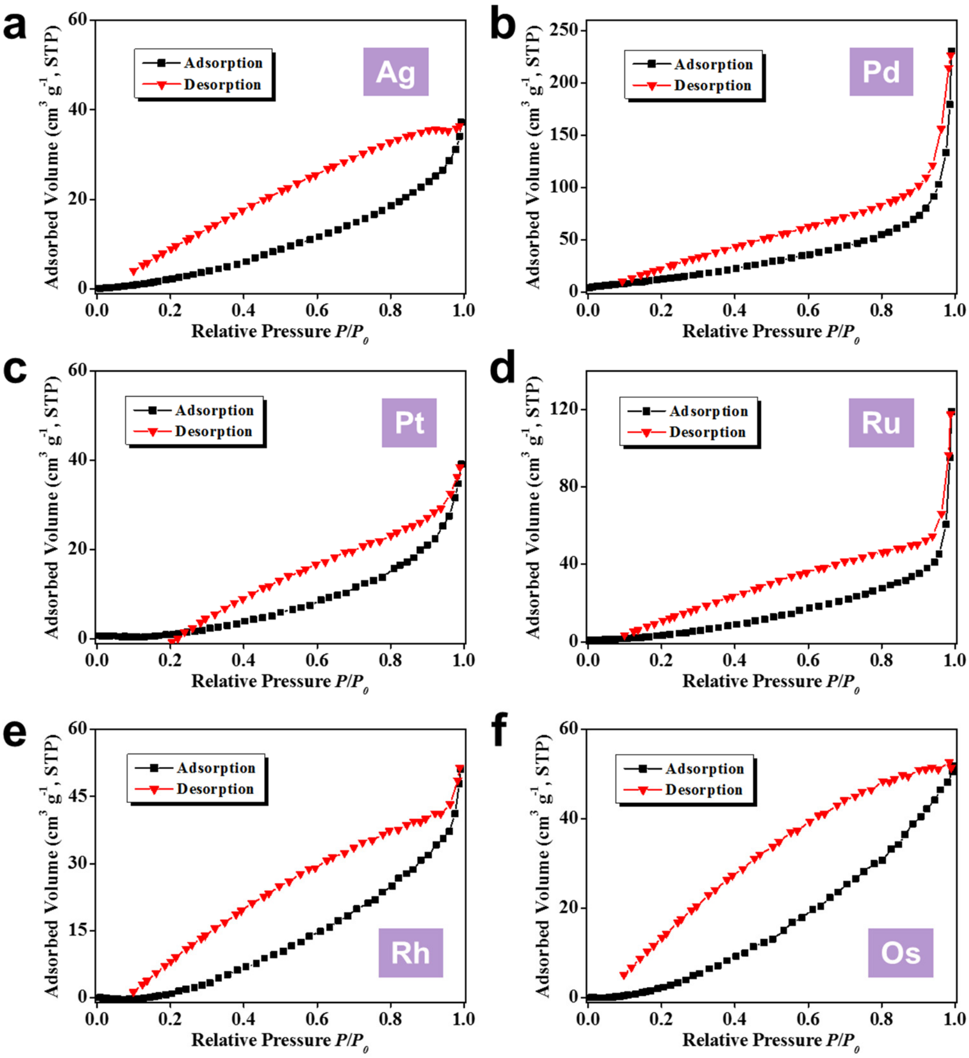

Supplementary Figure 24 | Nitrogen adsorption isotherms of single-metallic aerogels. (a) Ag, (b) Pd, (c) Pt, (d) Ru, (e) Rh, and (f) Os.

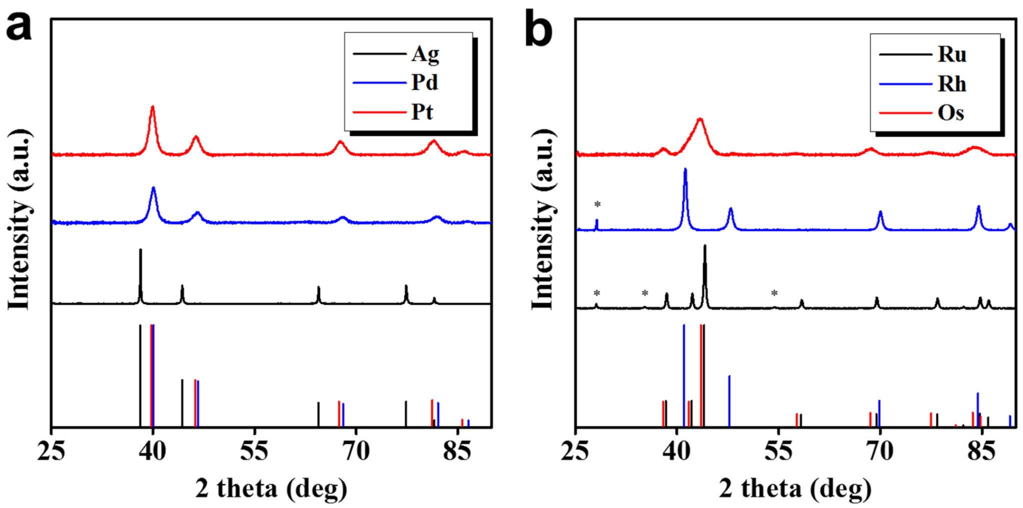

Supplementary Figure 25 | XRD patterns of single-metallic aerogels. The "*" mark the diffraction peaks from the corresponding metal oxides resulting from the spontaneous combustion of the fresh metal aerogels in air.

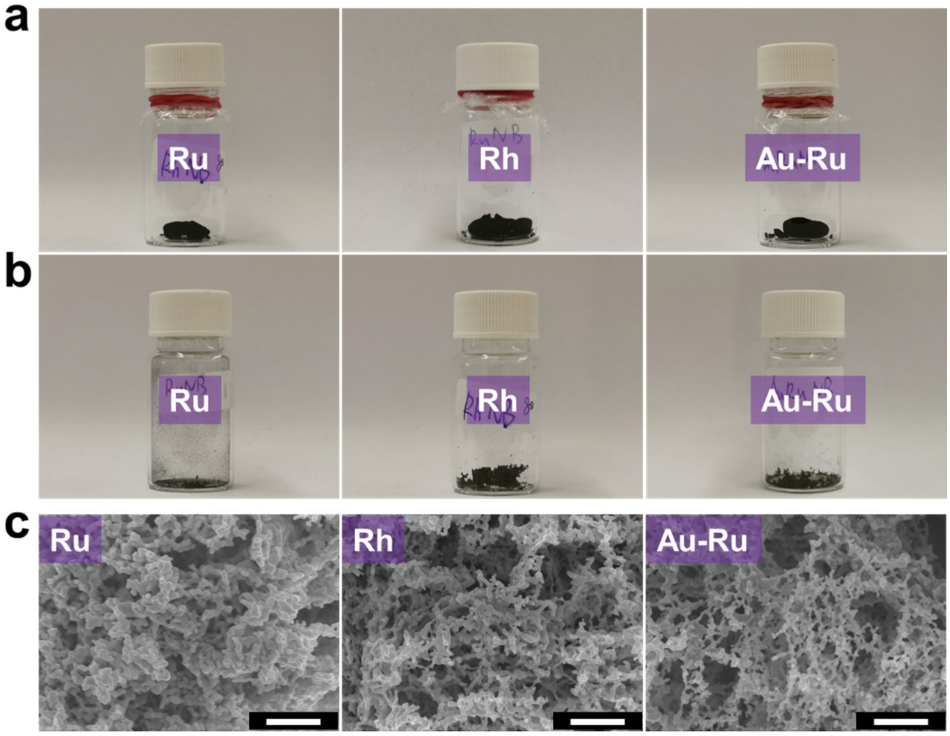

Supplementary Figure 26 | Aerogels with spontaneous combustion property. Photographs and SEM images of Ru, Rh, and Au-Ru aerogels (a) before and (b-c) after spontaneous combustion in air. Scale bars in figure c are 500 nm.

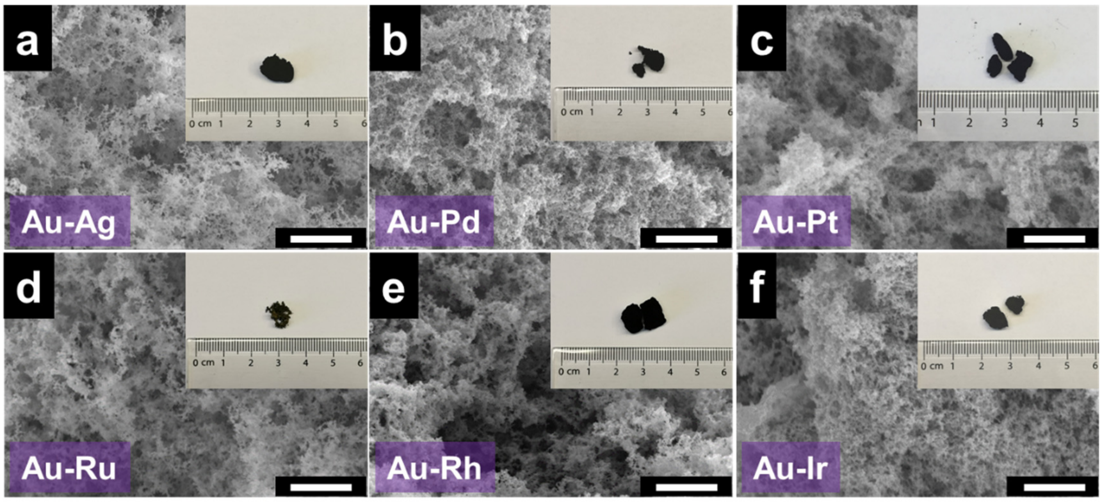

Supplementary Figure 27 | Au-based bimetallic aerogels. SEM images and photographs (inset) of (a) Au-Ag, (b) Au-Pd, (c) Au-Pt, (d) Au-Ru, (e) Au-Rh, and (f) Au-Ir aerogels. Scale bars in all figures are 500 nm.

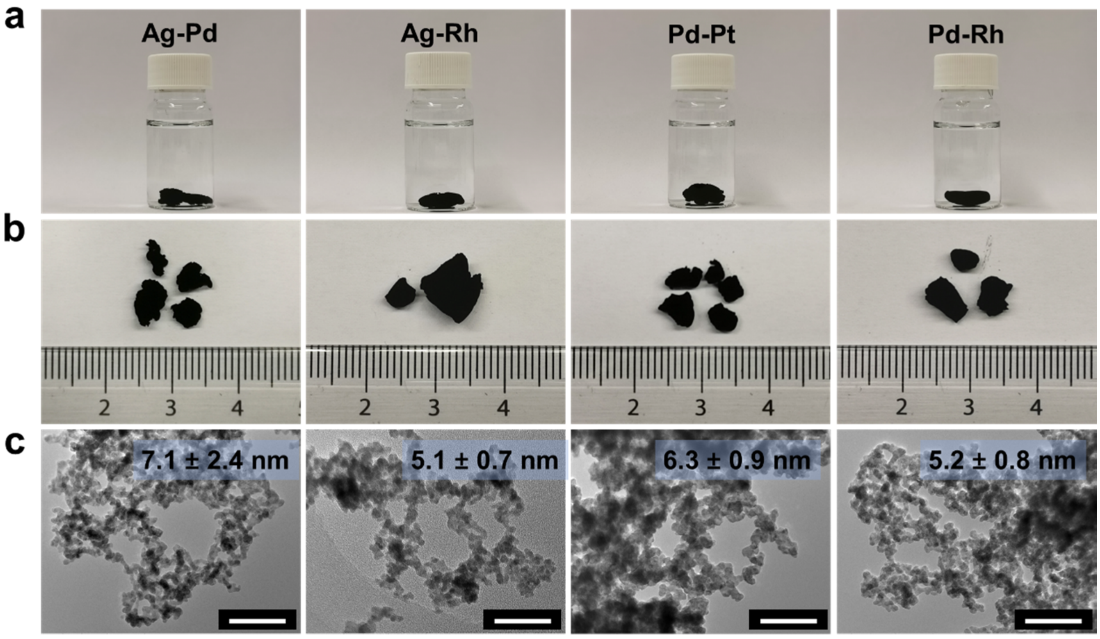

Supplementary Figure 28 | Non-Au-based bimetallic aerogels. (a-b) Photographs and (c) TEM images of various bimetallic hydrogels and aerogels, including Ag-Pd, Ag-Rh, Pd-Pt, and Pd-Rh. These gels were prepared from ~800 mL precursor solutions. Scale bars in figure c are 500 nm.

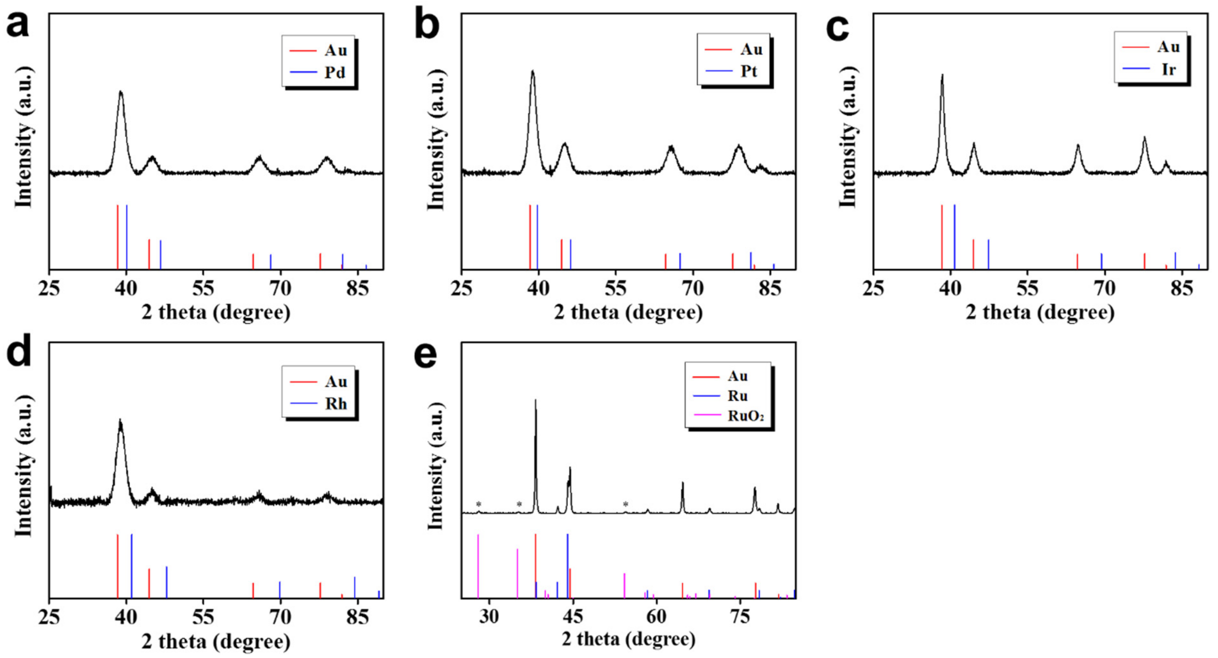

Supplementary Figure 29 | XRD patterns of bimetallic aerogels. (a) Au-Pd, (b) Au-Pt, (c) Au-Ir, (d) Au-Rh, and (e) Au-Ru. The "*" in (e) mark the diffraction peaks from RuO_2, which resulted from the spontaneous combustion of fresh Au-Ru aerogels in air.

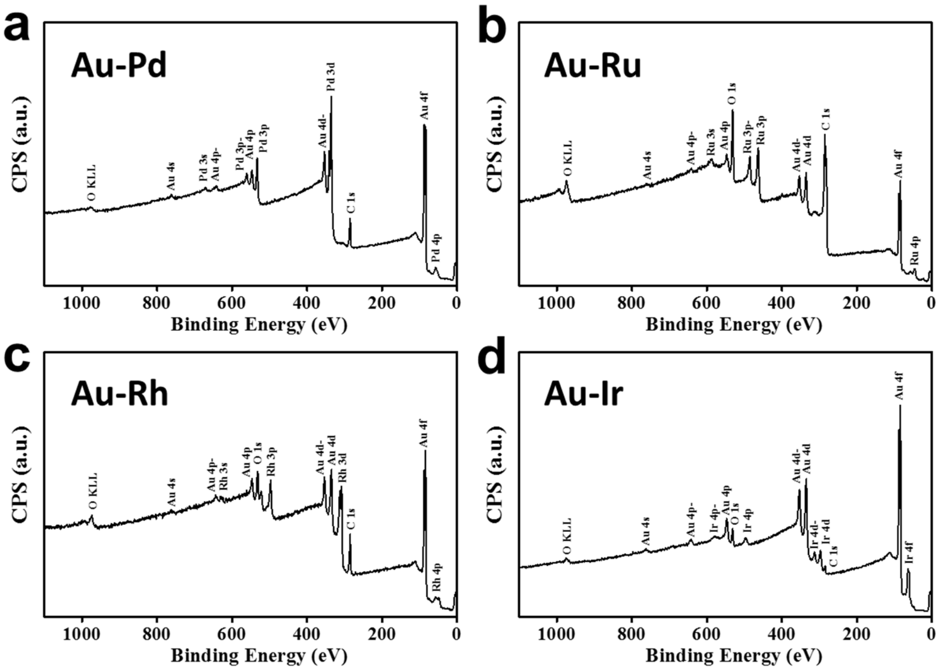

Supplementary Figure 30 | XPS spectra of bimetallic aerogels. (a) Au-Pd, (b) Au-Ru, (c) Au-Rh, and (d) Au-Ir.

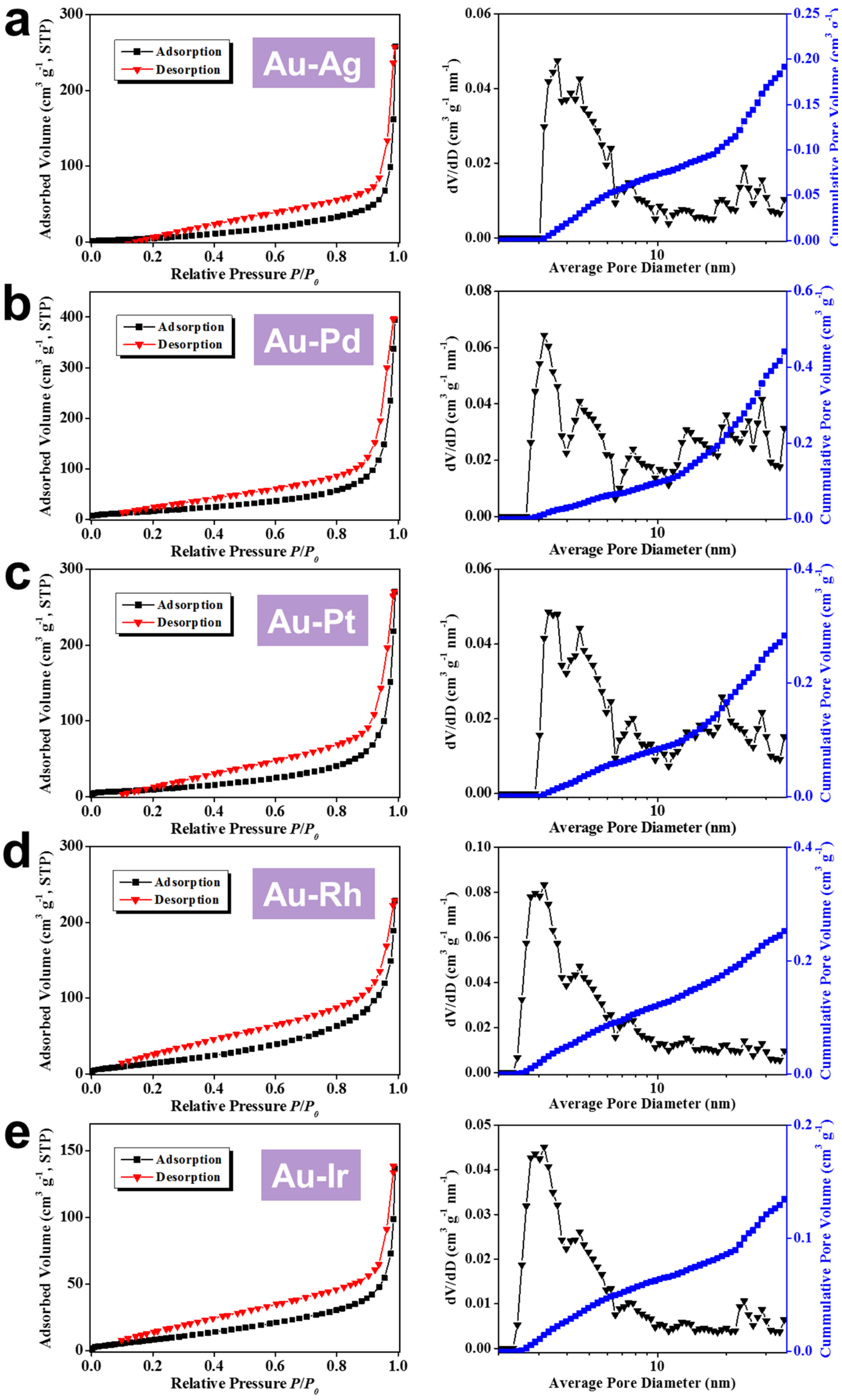

Supplementary Figure 31 | Nitrogen adsorption tests of bimetallic aerogels. Isotherms and pore size distributions of (a) Au-Ag, (b) Au-Pd, (c) Au-Pt, (d) Au-Rh, (e) Au-Ir.

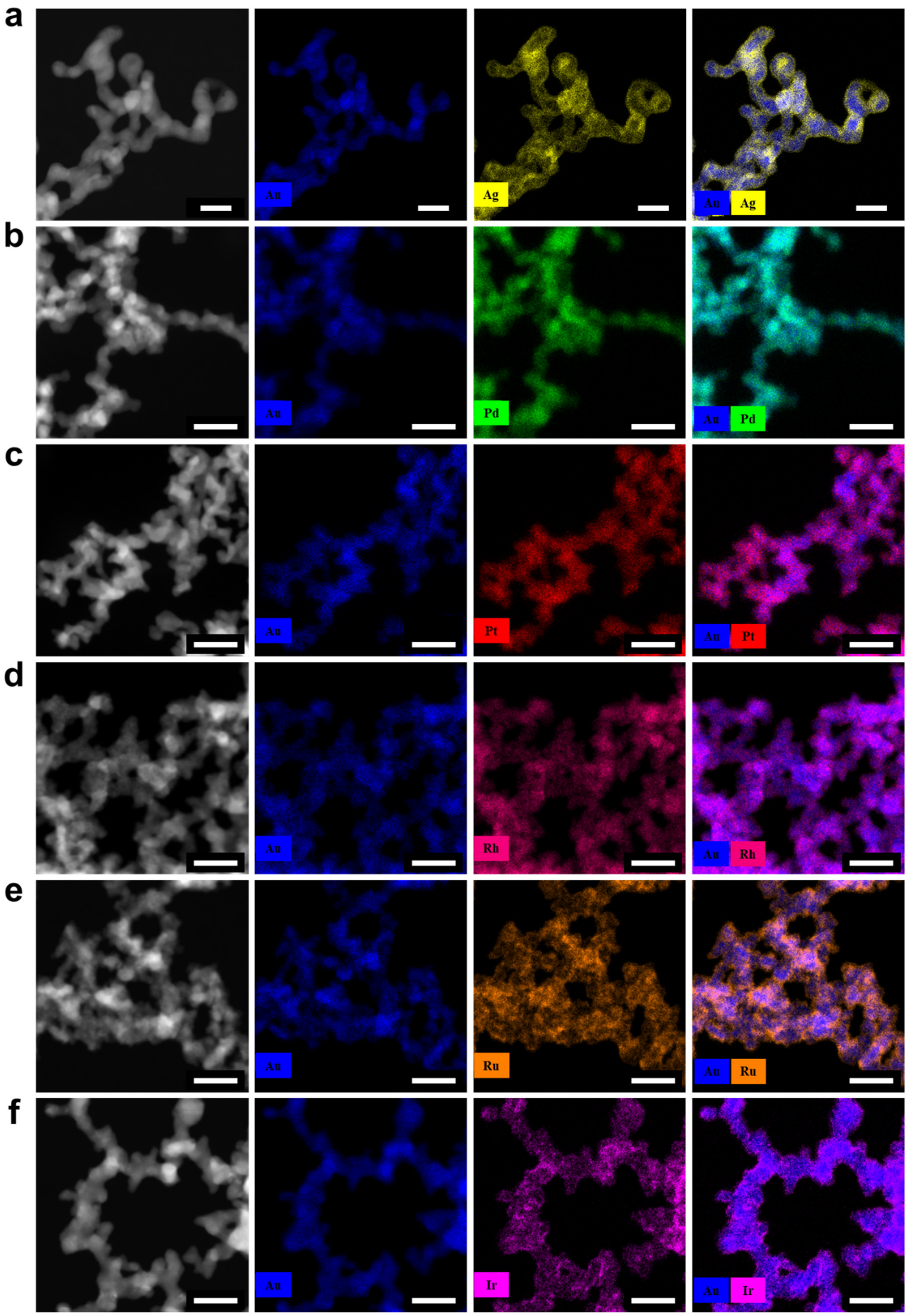

Supplementary Figure 32 | HAADF-STEM images and the corresponding EDX analysis of bimetallic aerogels. (a) Au-Ag, (b) Au-Pd, (c) Au-Pt, (d) Au-Rh, (e) Au-Ru, and (f) Au-Ir. Scale bars in all figures are 20 nm.

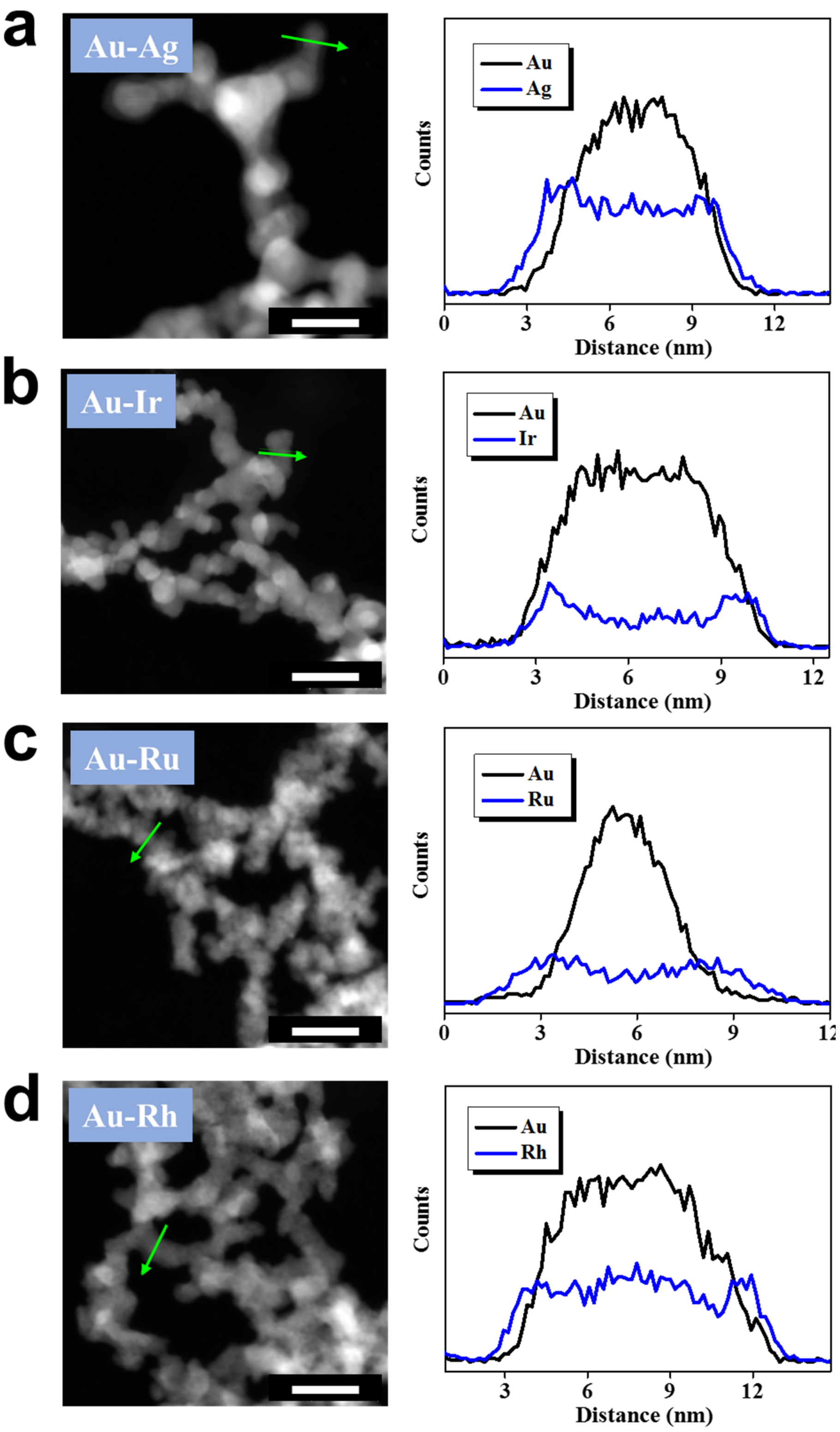

Supplementary Figure 33 | Characterization of core-shell-structured aerogels. High-angle annular dark-field scanning transmission electron microscopy imaging and EDX analysis (line scans) of (a) Au-Ag, (b) Au-Ir, (c) Au-Ru, and (d) Au-Rh aerogels. The shell thickness is 2-3 nm for the Au-Ru system, and 1-2 nm for the other systems. Scale bars in all figures are 20 nm.

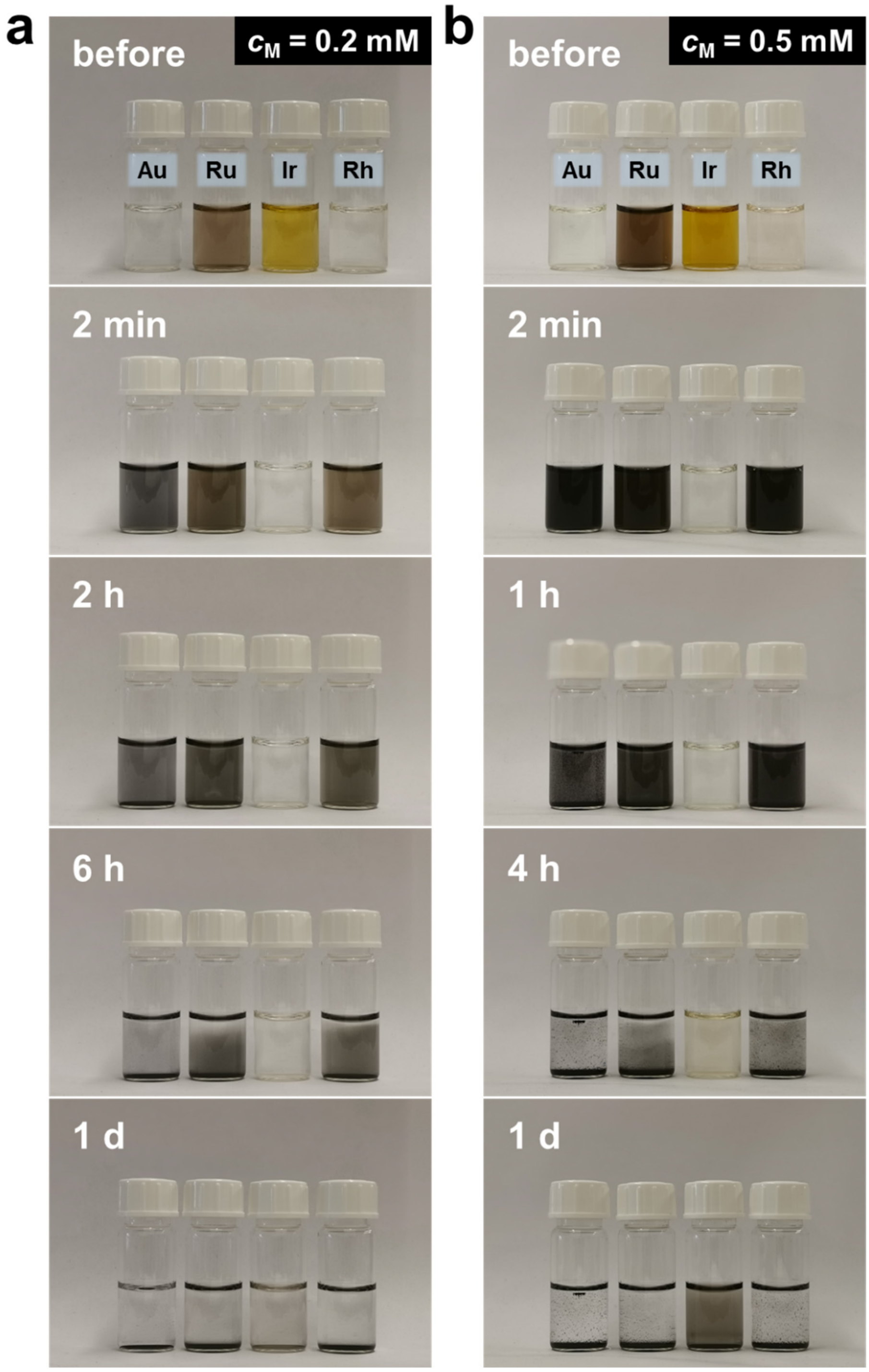

Supplementary Figure 34 | Comparison of the destabilization speed for different metal systems. Time-lapse photographs of the destabilization initiated by (a) $NaBH_4$ (c_M = 0.2 mM, R/M = 100) and (b) $NaBH_4$ (c_M = 0.5 mM, R/M = 100).

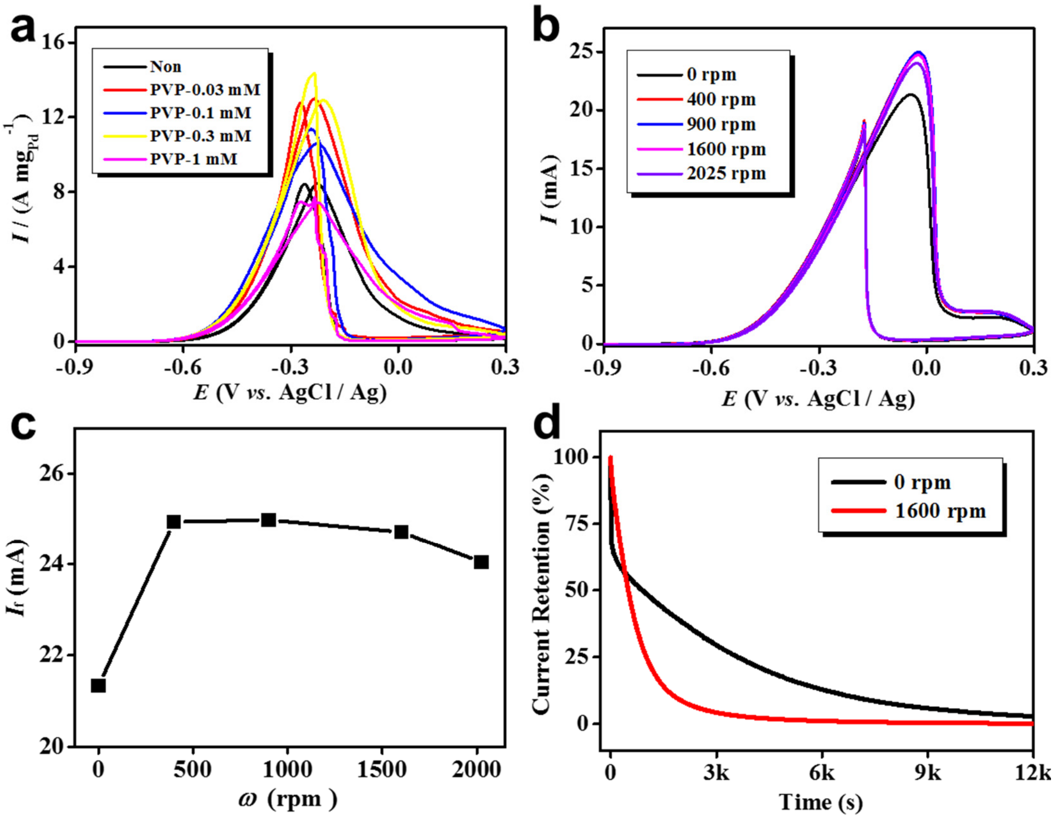

Supplementary Figure 35 | Characterization of the electrocatalytic EOR properties. (a) CV curves of the Au-Pd aerogels, where the catalyst inks were prepared in the presence of 0, 0.03, 0.1, 0.3, and 1 mM PVP. (b) CV curves (scan rate 50 mV s^{-1}), (c) summarized forward current, and (d) chronoamperometry tests of the Au-Pd aerogel catalyst performed on a rotation disk electrode against different rotation speeds. All tests were conducted in 1 M KOH + 1 M ethanol solution.

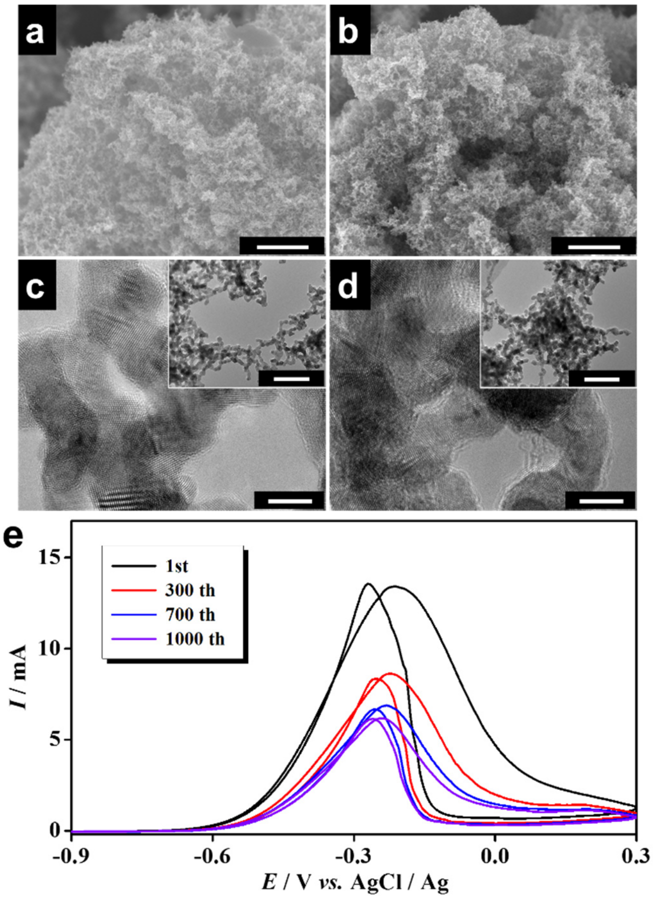

Supplementary Figure 36 | Cycling properties of the Au-Pd aerogels. (a-d) Evaluation of possible morphology changes of the Au-Pd aerogel (a,c) before and (b,d) after 1000 cycles, and (e) the corresponding cycling tests (scan rate: 100 mV s^{-1}). Scale bars in figures a, b are 500 nm, in c, d are 5 nm, and in the insets of figures c, d are 50 nm.

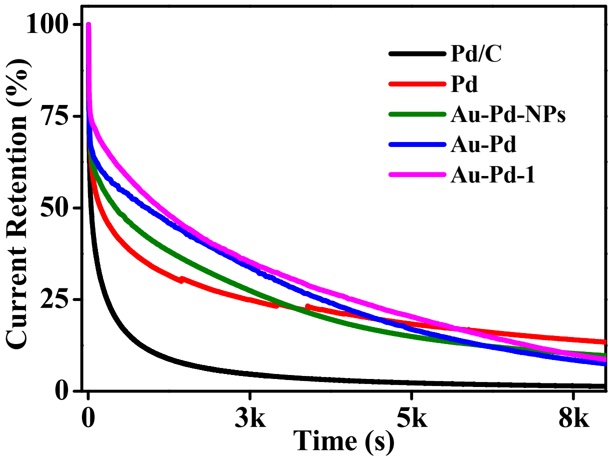

Supplementary Figure 37 | Chronoamperometry tests. Tests are performed in 1 M KOH + 1 M ethanol solution.

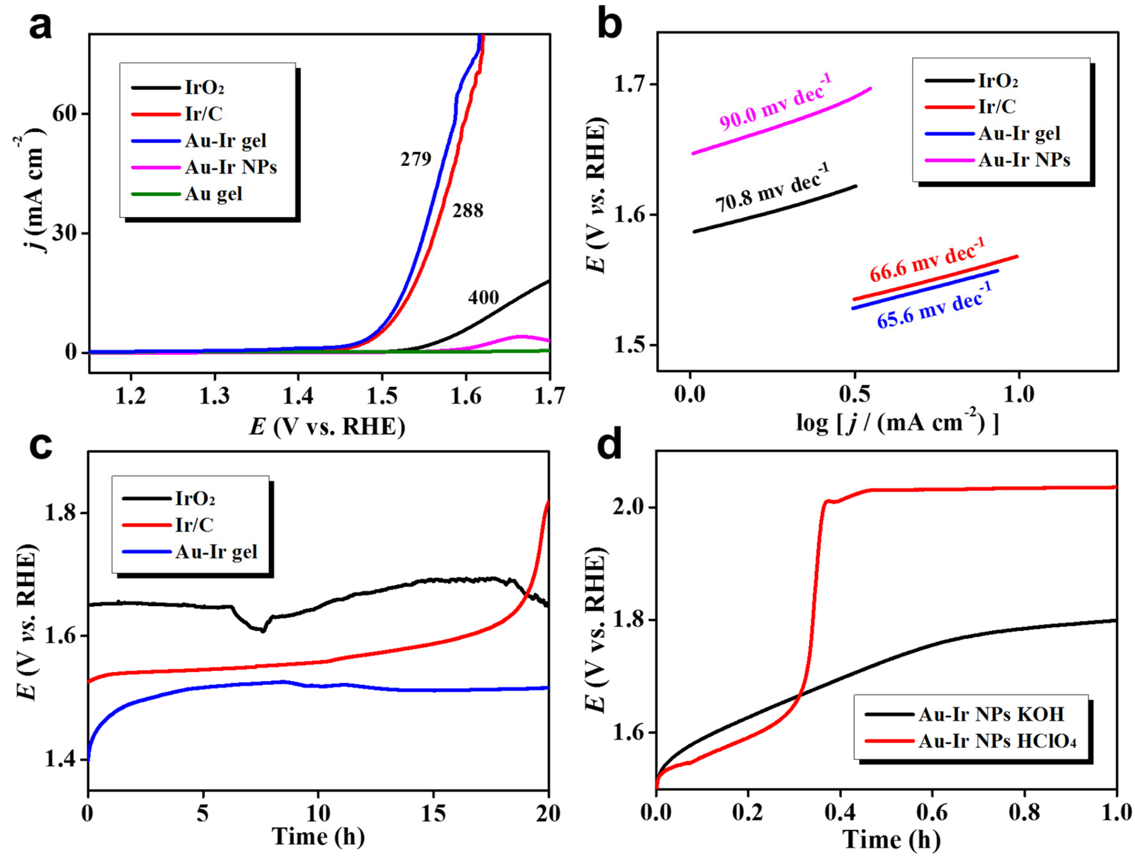

Supplementary Figure 38 | Characterization of the electrocatalytic OER properties in acidic medium. (a) LSV curves, (b) Tafel plots, and (c) chronopotentiometric tests (at 2 mA cm^{-2}) of commercial Ir/C, IrO_2, Au aerogel, Au-Ir aerogel, and Au-Ir NPs in 0.1 M $HClO_4$ aqueous solution. (d) Chronopotentiometric tests of Au-Ir NPs in 1 M KOH and 0.1 M $HClO_4$ aqueous solution.

Supplementary Tables

Supplementary Table 1 | Summary of parameters of as-prepared aerogels in this work. The specific surface area S_{BET} ($m^2 g^{-1}$) was calculated in the partial pressure (p/p_0) range of 0.1–0.3. The total pore volume V_{tot} was derived at $p/p_0 = 0.99$. The average ligament size d was obtained from a statistic analysis of TEM images. The density was estimated by averaging the values from at least 3 aerogels with relatively regular shape.

Entry	Metals	Ligands	S_{BET} ($m^2 g^{-1}$)	S_{BET} ($m^2 mol^{-1}$)	V_{tot} ($cm^3 g^{-1}$)	d (nm)	*Density* *(mg cm^{-3})*
1	Au[a]	/	3.5	682.4	0.063	27.9 ± 5.8	576.5
2	Au[a]	NaCA	4.8	945.2	0.066	21.5 ± 4.2	502.3
3	Au[a]	β-alanine	5.1	1006.3	0.065	11.6 ± 2.4	469.4
4	Au[a]	NaDC	21.0	4127.5	0.086	6.8 ± 1.5	277.3
5	Au[a]	PVP	59.8	11780.6	0.385	4.8 ± 0.9	60.5
6	Au[b]	PVP	44.0	8668.0	0.188	8.2 ± 1.7	85.1
7	Au[c]	PVP	35.4	6973.8	0.278	8.4 ± 1.5	82.6
8	Ag	/	2.1	223.9	0.058	48.2 ± 12.4	157.2
9	Pd	/	69.1	7354.4	0.357	5.6 ± 1.2	51.8
10	Pt[c]	/	13.2	2574.3	0.060	87.0 ± 31.0[d]	250.6
11	Os[b]	/	2.9	543.4	0.080	226.7 ± 94.0[d]	/
12	Ru[e]	/	3.8	389.1	0.184	3.9 ± 1.0	/
13	Rh[e]	/	3.4	353.0	0.079	4.7 ± 0.8	/
14	Au-Ag[f]	/	42.8	7487.0	0.408	7.6 ± 1.2	80.1
15	Au-Pd[f]	/	70.5	10701.5	0.610	4.3 ± 0.5	62.9
16	Au-Pt[f]	/	44.2	8665.4	0.425	/	76.0
17	Au-Ru[ef]	/	1.2	177.4	0.035	5.4 ± 0.9	64.8
18	Au-Rh[f]	/	72.9	10928.2	0.339	6.0 ± 0.7	46.7
19	Au-Ir[f]	/	42.6	8341.9	0.206	5.8 ± 1.0	169.8

[a] The concentration of the metal salt precursor is 0.2 mM. For other formulas which are not specified, the concentration is 0.5 mM.

[b] The concentration of the metal salt precursor is 1.0 mM.

[c] The concentration of the metal salt precursor is 5.0 mM.

[d] The value was obtained from the thickness of the backbone of the gel network.

[e] The gel displayed spontaneous combustion when exposed to air. The ligament size were calculated from unburned gels (the TEM samples were prepared from wet gels), while other parameters were obtained from burned gels.

[f] The molar ratio of the metals in the original metal salt solutions is 1 / 1.

Supplementary Table 2 | Comparison of various gold aerogels/foams reported in the literature.

**Materials**	**Methods**	**Gelation Time**	**Size (nm)**	**Surface area (m^2 g^{-1})**	**Ref**
NGFs [a]	Chemical dealloying	/	20-40	3.7	5
NGFs	Electrochemical dealloying	/	<6	/	6
NGFs	Electrochemical dealloying, annealing	/	10-1000	/	7
Au foams	Combustion	/	10-100	10.9	8
Au foams	Conc[b] & Direct freeze drying	/	200-500	Very small	9
Au aerogels	Conc[a] & H_2O_2	~1 week	100-500	Very small	10
Au aerogels	$NaBH_4$	2~12 h	4.8-38.3	3.5-59.8	**This work**
Au aerogels	Dopamine	6~72 h	5-6	50.1	11
Au aerogels	Salts	4-48 h	6.9-113.7	2.5-29.7	12
Au aerogels	Precursor 5-100 mM, $NaBH_4$, NaH_2PO_2, or dimethylamine borane	A few minutes	63.7 ± 36.0	3.1	13
Au aerogels	Precursor 12-24 mM, Hydrazine, 333 K	Several hours	21 ± 11	/	14

[a] NGFs, nanoporous gold foams.

[b] Concentration of nanoparticles solution by centrifuge filters.

Supplementary Table 3 | Element analysis of different bimetallic aerogels.

	Au-Pd	Au-Ir	Au-Rh
**XPS**	1/1.02	1/0.26	1/0.80
**ICP-OES**	1/1.05	1/0.27	1/0.89

Supplementary References

1. Frisch, M. J., Trucks, G. W., Schlegel, H. B., Scuseria, G. E., Robb, M. A., Cheeseman, J. R., Scalmani, G., Barone, V., Petersson, G. A., Nakatsuji, H., Li, X., Caricato, M., Marenich, A. V., Bloino, J., Janesko, B. G., Gomperts, R., Mennucci, B., Hratchian, H. P., Ortiz, J. V., Izmaylov, A. F., Sonnenberg, J. L., Williams, Ding, F., Lipparini, F., Egidi, F., Goings, J., Peng, B., Petrone, A., Henderson, T., Ranasinghe, D., Zakrzewski, V. G., Gao, J., Rega, N., Zheng, G., Liang, W., Hada, M., Ehara, M., Toyota, K., Fukuda, R., Hasegawa, J., Ishida, M., Nakajima, T., Honda, Y., Kitao, O., Nakai, H., Vreven, T., Throssell, K., Montgomery Jr., J. A., Peralta, J. E., Ogliaro, F., Bearpark, M. J., Heyd, J. J., Brothers, E. N., Kudin, K. N., Staroverov, V. N., Keith, T. A., Kobayashi, R., Normand, J., Raghavachari, K., Rendell, A. P., Burant, J. C., Iyengar, S. S., Tomasi, J., Cossi, M., Millam, J. M., Klene, M., Adamo, C., Cammi, R., Ochterski, J. W., Martin, R. L., Morokuma, K., Farkas, O., Foresman, J. B., Fox, D. J. *Gaussian 16 Rev. C.01*, Wallingford, CT, 2016.
2. Yang, Y., Weaver, M. N., Merz Jr, K. M., Assessment of the "6-31+ G**+ Lanl2dz" Mixed Basis Set Coupled with Density Functional Theory Methods and the Effective Core Potential: Prediction of Heats of Formation and Ionization Potentials for First-Row-Transition-Metal Complexes. *J. Phys. Chem. A* **113**, 9843-9851 (2009).
3. Tomasi, J., Mennucci, B., Cammi, R., Quantum Mechanical Continuum Solvation Models. *Chem. Rev.* **105**, 2999-3094 (2005).
4. Zhu, J., Chen, Z., Xie, M., Lyu, Z., Chi, M., Mavrikakis, M., Jin, W., Xia, Y., Iridium-Based Cubic Nanocages with 1.1-Nm-Thick Walls: A Highly Efficient and Durable Electrocatalyst for Water Oxidation in an Acidic Medium. *Angew. Chem. Int. Ed.* **58**, 7244-7248 (2019).
5. Zielasek, V., Jürgens, B., Schulz, C., Biener, J., Biener, M. M., Hamza, A. V., Bäumer, M., Gold Catalysts: Nanoporous Gold Foams. *Angew. Chem. Int. Ed.* **45**, 8241-8244 (2006).
6. Xu, C., Su, J., Xu, X., Liu, P., Zhao, H., Tian, F., Ding, Y., Low Temperature CO Oxidation over Unsupported Nanoporous Gold. *J. Am. Chem. Soc.* **129**, 42-43 (2007).
7. Li, R., Sieradzki, K., Ductile-Brittle Transition in Random Porous Au. *Phys. Rev. Lett.* **68**, 1168 (1992).
8. Tappan, B. C., Steiner, S. A., Luther, E. P., Nanoporous Metal Foams. *Angew. Chem. Int. Ed.* **49**, 4544-4565 (2010).
9. Freytag, A., Sánchez-Paradinas, S., Naskar, S., Wendt, N., Colombo, M., Pugliese, G., Poppe, J., Demirci, C., Kretschmer, I., Bahnemann, D. W., Behrens, P., Bigall, N. C., Versatile Aerogel Fabrication by Freezing and Subsequent Freeze-Drying of Colloidal Nanoparticle Solutions. *Angew. Chem. Int. Ed.* **55**, 1200-1203 (2016).
10. Bigall, N. C., Herrmann, A. K., Vogel, M., Rose, M., Simon, P., Carrillo-Cabrera, W., Dorfs, D., Kaskel, S., Gaponik, N., Eychmüller, A., Hydrogels and Aerogels from Noble Metal Nanoparticles. *Angew. Chem. Int. Ed.* **48**, 9731-9734 (2009).

11. Wen, D., Liu, W., Haubold, D., Zhu, C., Oschatz, M., Holzschuh, M., Wolf, A., Simon, F., Kaskel, S., Eychmüller, A., Gold Aerogels: Three-Dimensional Assembly of Nanoparticles and Their Use as Electrocatalytic Interfaces. *ACS Nano* **10**, 2559-2567 (2016).

12. Du, R., Hu, Y., Hübner, R., Joswig, J.-O., Fan, X., Eychmüller, A., Specific Ion Effects Directed Noble Metal Aerogels: Versatile Manipulation for Electrocatalysis and Beyond. *Sci. Adv.* **5**, eaaw4590 (2019).

13. Burpo, F. J., Nagelli, E. A., Morris, L. A., McClure, J. P., Ryu, M. Y., Palmer, J. L., Direct Solution-Based Reduction Synthesis of Au, Pd, and Pt Aerogels. *J. Mater. Res.* **32**, 4153-4165 (2017).

14. Tang, S., Vongehr, S., Wang, Y., Cui, J., Wang, X., Meng, X., Versatile Synthesis of High Surface Area Multi-Metallic Nanosponges Allowing Control over Nanostructure and Alloying for Catalysis and SERS Detection. *J. Mater. Chem. A* **2**, 3648-3660 (2014).
