# Supplementary material for: Unveiling reductant chemistry in fabricating noble metal aerogels for superior oxygen evolution and ethanol oxidation
Source: Nat Commun. 2020 Mar 27;11:1590. doi: 10.1038/s41467-020-15391-w (PMC7101436; doi:10.1038/s41467-020-15391-w)
Supplement: Supplementary file 2 — Peer Review [file 41467_2020_15391_MOESM2_ESM.pdf]

Reviewers' comments:

Reviewer #1 (Remarks to the Author):

In this manuscript, the authors demonstrate the method for the preparation of noble metal aerogels (NMAs) using the developed excessive-reductant-directed gelation strategy, and also discover and deeply investigate the various roles of the reductants in the gelating of noble metals including as reducing agent, stabilizer, and initiator. In all, the research topic is interesting because understanding and investigation of key factors (e.g. reductants and ligands) in the fabrication process for on-target material design. The present work may not only enrich the diversity of NMAs, but also open up a new dimension for devising efficient electrocatalysts based on NMAs and beyond. In this manuscript, the conclusions sound rational and supported with sufficient data and characterization techniques. This reviewer recommends publication of this manuscript after revisions.
1. Was the pH value of the system controlled in preparation of NMAs using the proposed method? Does the pH value affect the gelating process?
2. What about the effects of ion strength on the gelating process?

Reviewer #2 (Remarks to the Author):

In this work, the authors reported the synthesis of noble metal aerogels (NMAs) including gold aerogels and other 8 noble metals as well as their composition by an extremely powerful method — the excessive-reductant-directed gelation strategy. However, the morphology and structure of NMAs are rather similar to those of nanowire networks and nanowire assembly. What are the difference between NMAs and nanowire network of noble metal? In fact, there were plenty of works about the synthesis of nanowire networks made up of noble metal many year ago, say, Pt nanowire network (Nano Lett.2007, 7, 123650; J. Am. Chem. Soc. 2013, 135, 9480). The electrocatalytic performance are also rather remarkable. As for me, the products prepared by this method look more like precipitates of nanowire networks, instead of aerogels. I will reject this work considwering the high impact of Nature Comm. In addition, the following issues have to be properly addressed.
(1) The conclusion that the electrocatalysts boost the catalytic performance by 50% is not correct. The mass activity reported in literature are close to (10.8 A mg−1, in ACS Applied Materials & Interfaces 2014, 6, 12, 9481-9487;) or higher (33.1 A mgPd−1, ACS Applied Materials & Interfaces, 2018, 10, 1, 602-613) than that reported in this work (12.99 A mgPd−1). Thus, their electrocatalytic performance should be compared with that reported in literature to obtain the correct conclusion.
(2) The structure of as-prepared bimetallic NMAs are needed to be further investigated, core-shell structure or alloyed structure?
(3) In Figure 4c, the currents in CV curves of samples decrease rapidly, indicating the bad stability although the performance fade of Au-Pd-1 can be almost fully recovered after CV cleaning. However, it is impossible to clean the catalysts in practical applications. Thus, the stability is not good.
(4) Cycling test can be conducted and then the morphology of the samples before and after cycling test are compared.
(5) As Au and Pd also belong to noble metal, what is the catalytic performance of NMAs including all noble metals?
(6) Based on the data in Table S1, it seems that only Au and Pd aerogels cab be called as aerogels. In addition, only Au-based bimetallic aerogels can be fabricated. Other types of bimetallic aerogels can be fabricated (say, Ag-Pd, Pd-Pt, etc)?
(7) In their previous work (Adv. Mater. 2018, 1804881), the effect of drying method on the NMAs. In addition, the best way is supercritical carbon oxide drying, which were used in their most works. However, in this work, normal freezing drying was used. Why?
(8) The quality of the Figures are too poor. The words and numbers at X, Y coordinates cannot be observed clearly.

Reviewer #3 (Remarks to the Author):

Authors report the synthesis of Au aerogels via excessive NaBH4 reduction of HAuCl4, followed by freeze drying. In addition, they have extended this procedure to produce a library of aerogels including Ag, Pt, Pd, Ru, Rh, Os, and Ir. Although the synthesis of Au, Ag, Pt and Pd aerogels are not highly new, the formation of Ru, Rh, Os, and Ir gels and their alloys should be new and would be interesting for the materials community. However, following comments must be addressed prior to potential publication in nature communications.

1. This manuscript and supporting information contain a large amount of data and it is sometimes very difficult to track what is going on with different systems and synthesis conditions presented. Therefore, authors should condense the results and show only the most important data that support their investigations.

2. Figure 1: quality of the figures are low. It is very difficult to read y-axes of Figure 1d and 1e. Same applies to Figure 2 e, f, g. and Figure 3e,f,g. Also some of the TEM images are also very difficult to visualize (Figure 1b and 2b).

3. What are the zeta potential values of Au NPs stabilized by inorganic lions produced at intermediate R/M ratios? How do they compared to significantly destabilized NPs (or gels) produced with low R/M and high R/M ratios?

4. It is mentioned that Au gels shows record high surface area. 59m2/g is not a record high value for noble metal aerogels.

5. Figure 4, catalytic activity of aerogels should be compared with corresponding nanoparticle precipitates to have an understanding of the influences of gelation on EOR and OER. How does the catalytic activity of nanoparticles compared to those of Au-Ir and Au-Pd aerogels?

6. Experimental parameters for bi-metallic gels are vaguely presented in the supporting information. Details procedures are needed at least for the newly developed Ru, Rh, Os, and Ir gel systems.

Response to reviewer 1's comments

(All changes made are highlighted in blue in the revised manuscript)

Thank you very much for your elaborate comments and advice which will truly help us to improve the quality of our present manuscript and future research. We have carefully revised the manuscript according to your comments as follows:

In this manuscript, the authors demonstrate the method for the preparation of noble metal aerogels (NMAs) using the developed excessive-reductant-directed gelation strategy, and also discover and deeply investigate the various roles of the reductants in the gelating of noble metals including as reducing agent, stabilizer, and initiator. In all, the research topic is interesting because understanding and investigation of key factors (e.g. reductants and ligands) in the fabrication process for on-target material design. The present work may not only enrich the diversity of NMAs, but also open up a new dimension for devising efficient electrocatalysts based on NMAs and beyond. In this manuscript, the conclusions sound rational and supported with sufficient data and characterization techniques. This reviewer recommends publication of this manuscript after revisions.

Reply:

Thank you for your positive and insightful comments. We have addressed your comments point-by-point as follows.

1. Was the pH value of the system controlled in preparation of NMAs using the proposed method? Does the pH value affect the gelating process?

Reply:

The pH was not controlled during the fabrication process, which is due to the following reasons:

(1) Usually to provide a stable pH, the concentration of the buffer solution (*e.g.* PBS solution) needs to be quite high (*e.g.* > 0.1 M), where a large amount of ions will be introduced which would destabilize the precursor solution. As you mentioned, pH does affect the gelation process, but it is due to the salting-out effects that we have already reported in *Sci. Adv.* **2019**, *5*, eaaw4590, where both alkaline (*e.g.* 0.1 M KOH) and

acidic environments (*e.g.* 0.1 M HCl) can destabilize the metal nanoparticle solutions to yield either powders or gels. Therefore, it is not appropriate and realistic to control the pH in this work.

(2) The control of the pH will further raise the cost, particularly for mass production (*e.g.* nearly 1 L buffer solution will be needed for producing only < 80 mg gold aerogel).

2. What about the effects of ion strength on the gelating process?

Reply:

A high ionic strength can destabilize the charged nanoparticle (NP) solutions by screening the surface charge of the NPs. Therefore, the gelation induced by raising the ionic strength has been already reported several years ago (*Chem. Mater.* **2013**, *25*, 3528-3534.), where bimetallic alloy aerogels (Au/Ag, Pd/Ag, Pt/Ag) were prepared by adding NaCl (0.008 to 0.25 mol L^{-1}). In that work, it has been proven that only at a sufficiently high ionic strength, the gelation can take place.

However, during gelation, the NPs do not only simply aggregate, but also fuse together to form branched-nanowire-like structures, which are affected by the chemical nature of the used salts. Our recent work (*Sci. Adv.* **2019**, *5*, eaaw4590) disclosed detailed mechanisms regarding the specific ion effects to explain the gelation process and pointed out a cation-directed manipulation mechanism for controlling the ligament size of the resulting noble metal gels.

In the present work, however, we are mainly focusing on the gelation mechanisms triggered by the reductants, because gelation by using reductants (*e.g.* $NaBH_4$) has been widely applied in the field of noble metal aerogels, which can greatly simplify the fabrication procedures. Here, we not only clearly reveal the multiple functions of reductants (reducing agent, ligand, and initiator) for the first time, but also rationalize the effects of anions, especially for BH_4^-, in the gelation.

Since previous work has already investigated the effect of ionic strength, we did not explore this further. To provide more details on this, we added the new citation "*Chem. Mater.* **2013**, *25*, 3528-3534" as follows:

"20. K. G. Ranmohotti, X. Gao, I. U. Arachchige, *Chem. Mater.* **2013**, *25*, 3528-3534."

Response to reviewer 2's comments

(All changes made are highlighted in blue in the revised manuscript)

Thank you very much for your elaborate comments and advice which will really help us to improve the quality of our present manuscript and future research. We have carefully revised the manuscript according to your comments as follows:

In this work, the authors reported the synthesis of noble metal aerogels (NMAs) including gold aerogels and other 8 noble metals as well as their composition by an extremely powerful method — the excessive-reductant-directed gelation strategy. However, the morphology and structure of NMAs are rather similar to those of nanowire networks and nanowire assembly. What are the difference between NMAs and nanowire network of noble metal? In fact, there were plenty of works about the synthesis of nanowire networks made up of noble metal many year ago, say, Pt nanowire network (Nano Lett.2007, 7, 3650; J. Am. Chem. Soc. 2013, 135, 9480). The electrocatalytic performance are also rather remarkable. As for me, the products prepared by this method look more like precipitates of nanowire networks, instead of aerogels. I will reject this work considering the high impact of Nature Comm. In addition, the following issues have to be properly addressed.

Reply:

Thank you for your insightful comments. We would like to briefly address your questions here.

The definition for an aerogel is not only related to its morphology, since there are many other factors (that will be elaborated later). The focus of this work is also not only the electrocatalytic performance, but in addition to the development of new methodologies (reductant-directed gelation), a deep understanding of the underlying mechanisms for the gel formation, unusual organic-ligand-enhanced electrocatalysis, and certain insights for the performance decay of EOR.

(1) From TEM observation, the morphology of NMAs and nanowire networks are very similar, while the former can retain a free-standing architecture and appear as a monolith at the macroscale (*e.g.* millimeter, centimeter, or even larger), while the latter are usually characterized as unsupported powders. From the fabrication procedures, to create an aerogel, it needs (i) a sol-gel process to convert a colloidal solution to a free-standing wet gel and (ii) a procedure to remove the solvent from the wet gel while retaining its solid

network by special drying methods (usually by critical-point or freeze-drying). However, there are no such requirements for producing nanowire networks. It is not quite difficult to produce a nanowire network showing a self-supported structure at the microscale (*e.g.* <1 μm), while it needs much more delicate design and addressing additional scientific challenges to scale up this self-supported microscale architecture to a macroscopic scale.

(2) From Figure 1a, the reaction proceeds like a precipitation process, but a monolithic gel is produced instead of unsupported powders. The reason for this precipitation-like gelation behavior, *i.e.* gravity-driven sedimentation followed by automatic concentrating, has been elaborated in our previous work "*Sci. Adv.* **2019**, *5*, eaaw4590". From Figure 1a, 3h-k, S25, 29-31, it is clear that large noble metal monoliths with sizes on the centimeter-scale can be produced, evidencing the successful fabrication of aerogels in this work.

(3) The field of noble metal aerogels, which was pioneered by our group (see *Angew. Chem. Int. Ed.* **2009**, *48*, 9731-9734.), has developed for ten years with the efforts from our and many other groups (*e.g. Angew. Chem. Int. Ed.* **2010**, *49*, 4544-4565., *Angew. Chem. Int. Ed.* **2012**, *51*, 5743-5747., *Acc. Chem. Res.* **2015**, *48*, 154-162., *Adv. Mater.* **2016**, *28*, 8779-8783., *Adv. Mater. **2018**, 30, 1706755.*, *Matter* **2019**, *1*, 39-56.). The materials produced in our present work are comparable to previous studies regarding the general synthesis procedures and the macro-/microstructures, and they also meet the common features of aerogels (e.g. low densities (compared to the corresponding bulk materials), high porosities, 3D macroscopic self-supported structure, *etc.*). Hence, our materials are noble metal aerogels, not nanowire networks.

(4) For the electrocatalytic performance, we will show below the pronounced performance of our materials compared to the literature cited by the reviewer. At this point, we would like to stress that, in addition to a simple demonstration of the electrocatalytic performance as usually reported in other works, we have also gone beyond as we address the mechanisms to explain the performance decay as well as the unusual organic-ligand-directed performance enhancement, which provide new insights for understanding and promoting electrocatalyst design based on NMAs and beyond.

Now, we will address your comments point-by-point as follows.

1. The conclusion that the electrocatalysts boost the catalytic performance by 50% is not correct. The mass activity reported in literature are close to (10.8 A mg–1, in ACS Applied Materials & Interfaces 2014, 6, 12, 9481-9487;) or higher (33.1 A mgPd–1, ACS Applied Materials & Interfaces, 2018, 10, 1, 602-613) than that reported in this

work (12.99 A mgPd–1). Thus, their electrocatalytic performance should be compared with that reported in literature to obtain the correct conclusion.

Reply:

Thank you for your suggestion.

First, the performance enhancement of 50% is not related to previous literature, but to the electrocatalytic performance of as-obtained NMAs when the catalyst ink was prepared with and without the introduction of organic ligands. To make this point clearer, we have made a few changes to our description:

"Moreover, an unconventional organic-ligand-enhancing effect was discovered to boost the electrocatalytic performance by 50% compared to the ligand-free samples."

The two papers mentioned in the referee report quite high current densities, though those are not for aerogels. We have cited them according to your suggestion:

"41. Hong, W., Wang, J., Wang, E., Facile Synthesis of Highly Active PdAu Nanowire Networks as Self-Supported Electrocatalyst for Ethanol Electrooxidation. *ACS Appl. Mater. Interfaces* **6**, 9481-9487 (2014).

42. Wang, J., Zhang, P., Xiahou, Y., Wang, D., Xia, H., Möhwald, H., Simple Synthesis of Au–Pd Alloy Nanowire Networks as Macroscopic, Flexible Electrocatalysts with Excellent Performance. *ACS Appl. Mater. Interfaces* **10**, 602-613 (2018)."

"The electrocatalytic ethanol oxidation, serving as a model reaction that takes place at the anode of direct ethanol fuel cells, has been extensively studied by using a wide range of Pd- and Pt-based NMAs[6,7] and nanowire networks.[41,42]"

2. The structure of as-prepared bimetallic NMAs are needed to be further investigated, core-shell structure or alloyed structure?

Reply:

Thank you for your suggestion.

For the bimetallic NMAs, we have the STEM-EDX data interspersed in the main text and the supplementary information. According to your suggestion, we have made a summary figure for the STEM-EDX investigations of the various NMAs in the supplemental information:

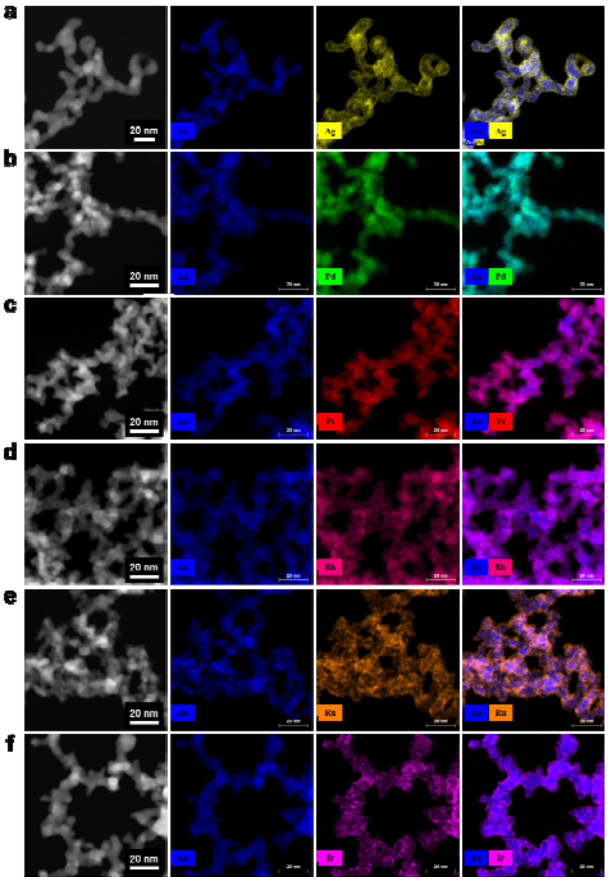

Supplementary Figure 32. HAADF-STEM images and the corresponding EDX analysis of various bimetallic aerogels. (a) Au-Ag, (b) Au-Pd, (c) Au-Pt, (d) Au-Rh, (e) Au-Ru, and (f) Au-Ir.

3. In Figure 4c, the currents in CV curves of samples decrease rapidly, indicating the bad stability although the performance fade of Au-Pd-1 can be almost fully recovered after CV cleaning. However, it is impossible to clean the catalysts in practical applications. Thus, the stability is not good.

Reply:

Thank you for your suggestion.

The rapid current decay is a common unsolved issue for the electro-oxidation of ethanol. Here, despite a rapid decay, the stability of the Au-Pd aerogel still greatly surpasses that of commercial Pt/C and other catalysts. After a chronoamperometry evaluation for 1000 s, the current retention for the Au-Pd-1 aerogel is 51.7%, which is much better than that for the Au-Pd aerogel (48.8%), Au-Pd nanoparticles (40.9%), Pd aerogel (33.9%),

commercial Pd/C (10.6%), and the cited works (15-20%) from question 1. We have added a figure for a comparison of the current retention and the corresponding description in the main text:

"It is worth mentioning that despite of a considerable performance fade for all investigated materials, the stability of Au-Pd-1 greatly surpasses that of commercial Pd/C (51.7% *vs.* 10.6% current retention after chronoamperometry evaluation for 1000 s, see **Supplementary Figure 37**)."

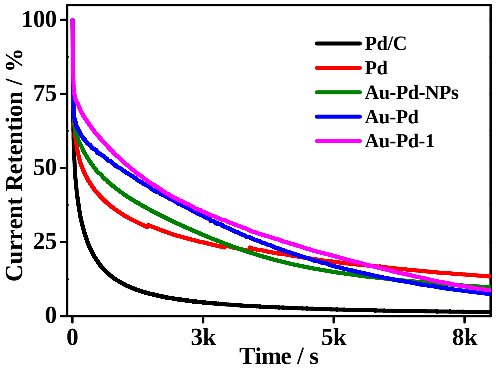

Supplementary Figure 37. Current retention of various catalysts during chronoamperometry evaluation, as performed in 1 M KOH + 1 M ethanol solution.

Here, the CV-recovery data is just to present the reversibility of the performance decay and to help us to analyze the possible decay mechanism. In the electrocatalytic part, we focus on two points, *i.e.* (1) the organic ligands (PVP) can surprisingly boost the performance, and (2) the mechanisms for the rapid performance decay may be the temporary blocking of the active sites by the intermediates. For practical use, it still needs much more efforts to address the stability issue, while it is not in the focus of the current study. In the main text, we have clarified this as follows:

"However, for practical applications, further efforts to improve the long-term stability of electrocatalysts for EOR are needed."

4. Cycling test can be conducted and then the morphology of the samples before and after cycling test are compared.

Reply:

Thank you for your comments.

We have compared the morphology of the Au-Pd aerogel before and after cycling tests by using a gold foil as the working electrode. However, from the observations of both SEM and TEM images, we did not notice any significant changes, and this is also consistent with our conclusion that the performance decay does not come from irreversible morphology changes. We have added corresponding figures and descriptions as follows:

"This was also verified by SEM and TEM analyses, where no obvious changes in the morphology or ligament sizes were observed (**Supplementary Figure 36)**."

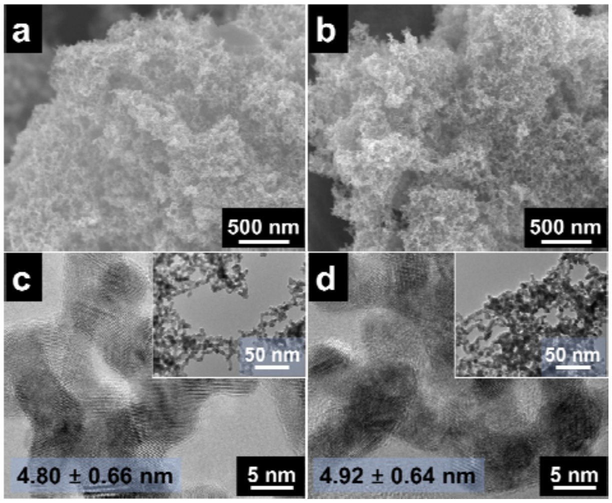

Supplementary Figure 36. Evaluation of possible morphology changes of the Au-Pd aerogel (a,c) before and (b,d) after a chronoamperometry test for 8 h.

5. As Au and Pd also belong to noble metal, what is the catalytic performance of NMAs including all noble metals?

Reply:

Thank you for your suggestion.

Because Au is usually regarded as inactive to the studied electrocatalytic reaction (it is used for providing electron transfer pathways as indicated in the manuscript), we normalized the mass activity by using only the active noble metals, *i.e.* Pd for EOR. In various other reports where multiple noble metals are present, also normalization is done with respect to the active metal only for studying the mass activity, such as in *ACS Appl. Mater. Interfaces* **2014**, *6*, 9481-9487., *Sci. Adv.* **2019**, *5*, eaaw4590, *Angew. Chem. Int. Ed.* **2018**, *57*, 2963-2966, *Nano Energy* **2018**, *53*, 206-212. *etc.*

We agree that from the cost aspect, it is also good to know the mass activity based on all noble metals, such as in the mentioned paper "*ACS Appl. Mater. Interfaces* **2018**, *10*, 602-613.". Therefore, we have calculated the mass activity normalized by the mass of all metals, where Au-Pd and Au-Pd-1 feature a mass activity of 2.77 and 3.79 A mg^{-1}, respectively, which are still higher than for the Pd aerogel (2.24 A mg^{-1}) and Pd/C (1.68 A mg^{-1}). The values are also higher than those for the Au-Pd alloy nanowires reported in *ACS Appl. Mater. Interfaces* **2018**, *10*, 602-613 (0.58-1.23 A mg^{-1}). This is added in the manuscript as follows:

"Even if the mass activity is normalized to all metals, the Au-Pd-1 still features a considerably higher performance than that of Pd/C (3.79 vs. 1.68 A mg^{-1})."

6. Based on the data in Table S1, it seems that only Au and Pd aerogels can be called as aerogels. In addition, only Au-based bimetallic aerogels can be fabricated. Other types of bimetallic aerogels can be fabricated (say, Ag-Pd, Pd-Pt, etc)?

Reply:

Thank you for your comments. It is, however, difficult for us to understand why – based on the data presented in Table S1 – only the Au and Pd aerogels shall be called aerogels, maybe based on the mass densities and specific surface areas?

Firstly, we would like to clarify, that the aerogel is not defined by density or specific surface area (there is various literature reporting aerogels with large densities or low surface areas, especially for NMAs because of the considerably larger density of noble metals compared with other materials), but usually by its structural features (e.g. a self-supported, porous, 3D architecture that is capable of extending to the macroscale) and the fabrication methods (sol-gel process followed by special drying). We have already discussed this question above, directly below your general comment. Therefore, all materials shown in Table S1 are aerogels.

Secondly, we just used Au-based bimetallic aerogels as examples to demonstrate the generality of our strategy, while various bimetallic aerogels without gold can be created by our method, too, such as Ag-Pd, Pd-Pt, Ag-Rh, and Pd-Rh. According to your suggestion, we have fabricated the as-mentioned gels and added the corresponding description and displays into the manuscript:

"Notably, besides gold-based bimetallic NMAs, various other NMAs such as Ag-Pd, Ag-Rh, Pd-Pt, and Pd-Rh aerogels can also be prepared with the same procedure. These

aerogels were characterized by various techniques as shown in the **Supplementary Figures 27-31**, cf. also **Supplementary Table S1**."

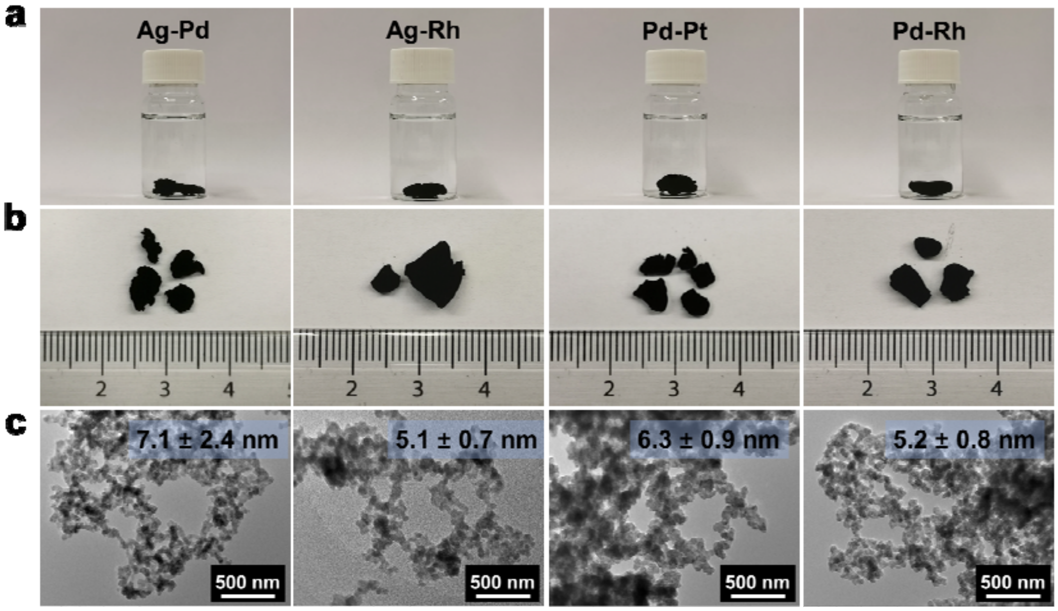

Supplementary Figure 28. (a-b) Photographs and (c) TEM images of various bimetallic hydrogels and aerogels, including Ag-Pd, Ag-Rh, Pd-Pt, and Pd-Rh.

7. In their previous work (Adv. Mater. 2018, 1804881), the effect of drying method on the NMAs. In addition, the best way is supercritical carbon oxide drying, which were used in their most works. However, in this work, normal freezing drying was used. Why?

Reply:

Thank you for your suggestion.

Supercritical CO_2 drying is regarded as the most efficient way to produce aerogels, especially in industry, and it can give rise to materials with better mechanical strength and less structure shrinkage compared to other drying methods. However, supercritical CO_2 drying is not a widespread technique, and it is much more complicated, more dangerous, and more expensive in comparison with freeze drying. To name a few requirements for it: (1) before supercritical drying, samples need to undergo additional solvent exchange processes with high-purity ethanol or acetone; (2) samples need to be carefully transferred to a special container before drying; (3) high-pressure is needed to

realize the supercritical state for CO_2; (4) the control of the temperature, pressure, rate for discharging solvent (a mixture of ethanol/acetone and CO_2) need to be delicately optimized, and it usually requires quite some experience especially for scaling up. In contrast, the freeze dryer is well commercialized and cheaper, and one can directly use the as-prepared hydrogels in any kind of container for freeze drying. According to our previous experience, although freeze drying yields aerogels with weaker mechanical strength compared to supercritical drying, the parameters related to electrocatalysis, such as the specific surface area, do not deviate significantly.

Therefore, we took the freeze drying process to create aerogels for this work (as e.g. also for our previous work, such as *Sci. Adv.* **2019**, *5*, eaaw4590.), expecting to simplify procedures, reduce costs, and also involve more potential researchers to the field of NMAs.

8. The quality of the Figures are too poor. The words and numbers at X, Y coordinates cannot be observed clearly.

Reply:

Thank you for your suggestion. Sorry for the inconvenience, we have increased the resolution for all figures.

Response to reviewer 3's comments

(All changes made are highlighted in blue in the revised manuscript)

Thank you very much for your elaborate comments and advices which will really help us to improve the quality of our present manuscript and future research. We have carefully revised the manuscript according to your comments as follows:

Authors report the synthesis of Au aerogels via excessive NaBH4 reduction of HAuCl4, followed by freeze drying. In addition, they have extended this procedure to produce a library of aerogels including Ag, Pt, Pd, Ru, Rh, Os, and Ir. Although the synthesis of Au, Ag, Pt and Pd aerogels are not highly new, the formation of Ru, Rh, Os, and Ir gels and their alloys should be new and would be interesting for the materials community. However, following comments must be addressed prior to potential publication in nature communications.

Reply:

Thank you for your insightful and positive comments. We have addressed your comments point-by-point as follows.

1. This manuscript and supporting information contain a large amount of data and it is sometimes very difficult to track what is going on with different systems and synthesis conditions presented. Therefore, authors should condense the results and show only the most important data that support their investigations.

Reply:

Thank you for your suggestion.

We have condensed the results, particularly the figures. Additionally, we have clarified the synthesis conditions for different systems, which will be further addressed in the sixth question raised by you. For example, we have combined some figures, see *e.g.* **Supplementary Figures 16-18**. Also we have re-arranged some of the descriptions in the main text.

2. Figure 1: quality of the figures are low. It is very difficult to read y-axes of Figure 1d and 1e. Same applies to Figure 2 e, f, g. and Figure 3e,f,g. Also some of the TEM images are also very difficult to visualize (Figure 1b and 2b).

Reply:

Thank you for your suggestion. Sorry for the inconvenience, we have increased the resolution for all figures.

3. What are the zeta potential values of Au NPs stabilized by inorganic ions produced at intermediate R/M ratios? How do they compared to significantly destabilized NPs (or gels) produced with low R/M and high R/M ratios?

Reply:

According to your suggestion, we have further investigated the zeta potentials of Au NPs with different R/M ratios, where zeta potentials of -14.2, -32.0, -35.4, and -38.9 mV were found for Au sols with R/M = 1, 4, 50, and 100, respectively. The more negative values with increasing $NaBH_4$ can be attributed to the increasing adsorption of BH_4^-. Due to their large absolute zeta potential, the NP solutions are expected to be stable for R/M = 50 and 100. However, as we elaborated in the manuscript (Figure 2h), the gelation induced by $NaBH_4$ is a rather complicated process. Although BH_4^- can prevent the aggregation of the NPs by electrostatic repulsion, the adsorbed BH_4- can spontaneously decompose to loosely bonded BO_2^-, which is easily desorbed from the NPs, thus inducing the fusion of NPs. Therefore, it is not possible to explain all processes from the zeta potential measurements alone. We have added the corresponding results and description as follows:

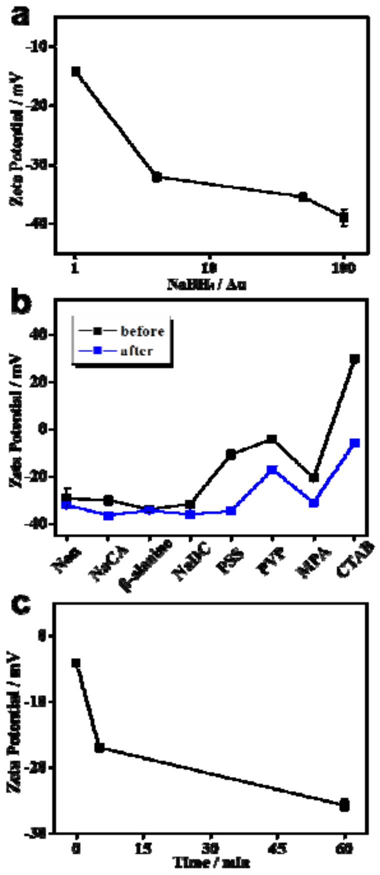

Supplementary Figure 10. Zeta potential of gold NP solutions (c_M = 0.2 mM). (a) The Zeta potential of freshly prepared gold solutions after reducing $HAuCl_4$ by adding various amounts of $NaBH_4$. (b) The Zeta potential of various ligand-stabilized gold NP solutions before (black line) and after (blue line) destabilization by $NaBH_4$ for *ca.* 5 min. (c) Time-lapse zeta potential evolution of PVP-coordinated gold NP solutions as destabilized by $NaBH_4$.

"Notably, due to its high affinity to gold (E_b = 10.3 kcal mol^{-1} for BH_4^-), the ligand exchange process with BH_4^- should be more effective than for common anions as analyzed above (1.51–8.40 kcal mol^{-1}). This can also be evidenced from zeta potential measurements, where a more negative zeta potential was obtained with increasing ratio of $NaBH_4$/Au (**Supplementary Figure 10**)."

4. It is mentioned that Au gels shows record high surface area. 59m2/g is not a record high value for noble metal aerogels.

Reply:

Thank you for your suggestion.

In fact, we have addressed the record-high surface area to the reported gold aerogels, not to all noble metal aerogels, see "*affording a gold aerogel with a record-high specific surface area of 59.8 m^2 g^{-1}, thus rendering the as-obtained material with the thinnest ligament size and highest SSA among all gold aerogels reported to date*".

Before the current work, the largest specific surface areas obtained for gold aerogels were 50.1 m^2 g^{-1}, while 18 wt.% β-CD remained in that aerogel (*ACS Nano* **2016**, *10*, 2559-2567.). Only few other works could produce monolithic gold aerogels with a surface area larger than 15 m^2 g^{-1} (see Table S2 in the supplemental information), because it is still a great challenge to prepare large-surface-area porous gold, though many high-surface-area aerogels made of other noble metals (e.g. Pd, Pt, Ag) have been reported. Moreover, because of the difference of the physical parameters (e.g. molecular weight and fundamental physical properties) and chemical properties (e.g. the gelation behavior) between gold and other noble metals, it might be only reasonable to compare the surface areas among gold aerogels instead of including other noble metal aerogels.

5. Figure 4, catalytic activity of aerogels should be compared with corresponding nanoparticle precipitates to have an understanding of the influences of gelation on EOR and OER. How does the catalytic activity of nanoparticles compared to those of Au-Ir and Au-Pd aerogels?

Reply:

Thank you for your suggestion.

We have measured the catalytic activity of the Au-Pd NPs for EOR, and the Au-Ir NPs for OER, respectively. In both cases, the gel displayed considerably better performance than the corresponding NPs. The results and description are shown below:

"Concentrated Au-Pd and Au-Ir bimetallic NP solutions were produced for comparing the electrocatalytic performance with that of the corresponding aerogels. Au-Pd and Au-Ir NP solutions were prepared by a similar procedure. Taking the preparation of the Au-Pd NP solution as an example, aqueous solutions of $HAuCl_4·3H_2O$ (32.5 mM, 3.075 mL)

and K_2PdCl_4 (32.5 mM, 3.075 mL) were added in water (374 mL) under stirring. Then, freshly prepared $NaBH_4$ aqueous solution (1.0 M, 20 mL) was rapidly injected, followed by stirring for 30–60 min. Afterwards, the solution was concentrated by ultracentrifugation, where the volume was reduced from ~400 mL to ~5 mL."

"EOR tests of commercial Pd/C were performed together with those on the Pd aerogel, the Au-Pd aerogel, and the Au-Pd NPs (**Figure 4a-b**)."

"Commercial Pd/C, the Pd aerogel, and Au-Pd NPs delivered inferior I_f (1.68, 2.24, and 3.56 A mg_{Pd}^{-1}) and I_f/I_b (0.87, 0.86, and 0.93) compared to the Au-Pd aerogel (I_f = 8.45 A mg_{Pd}^{-1}, I_f/I_b = 1.00)."

"As shown in **Figure 4d-e**, Au-Ir core-shell aerogels manifested a considerably small overpotential and Tafel slope (245 mV and 36.9 mV dec^{-1}), outperforming commercial Ir-based catalysts (e.g. Ir/C, IrO_2 ≥ 311 mV and ≥ 54.3 mV dec^{-1}), gold aerogels, and the Au-Ir NPs in alkaline environment. Additionally, a long-term chronopotentiometric test elucidated an excellent stability of the as-prepared Au-Ir aerogel with an overpotential of less than 250 mV after more than 12 hours of operation. In contrast, the performance of unsupported Au-Ir NPs rapidly decays within an hour (**Supplementary Figure 38**), which can be ascribed to the NP detaching resulting from continuous operation."

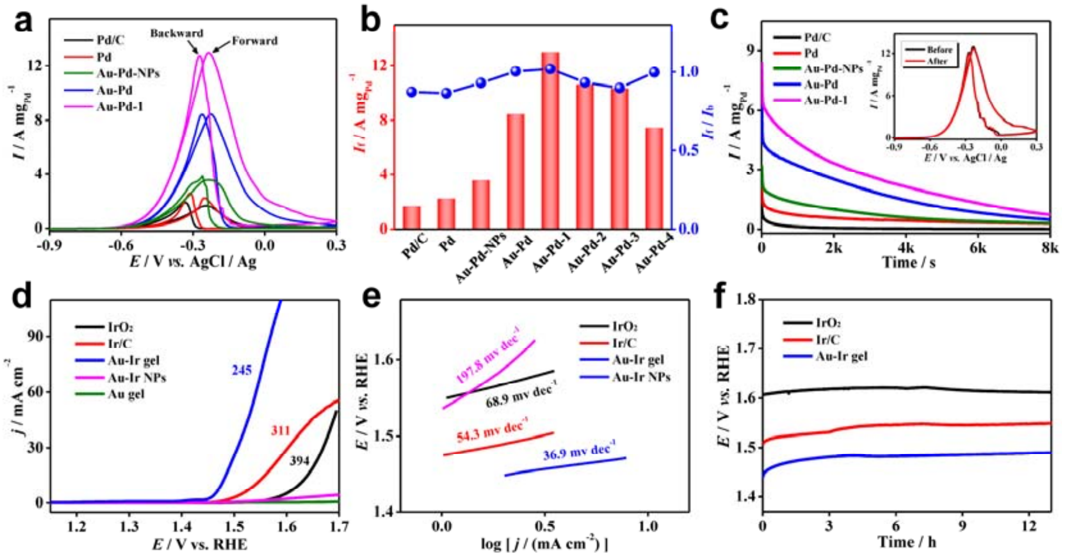

Figure. 4 Electrocatalytic performance of EOR and OER. (a) CV curves (scan rate 50 mV s^{-1}), (b) summarized I_f and I_f/I_b, and (c) chronoamperometry tests of commercial Pd/C, Au-Pd NPs, and various aerogel catalysts in 1.0 M KOH + 1.0 M ethanol solution. The inset of (c) shows the CV curves of Au-Pd-1 before and after a 3 h chronoamperometry evaluation. (d) LSV curves (scan rate 5 mV s^{-1}), (e) Tafel plots, and

(f) chronopotentiometric evaluation of commercial Ir/C, IrO_2, Au aerogel, Au-Ir NPs, and Au-Ir aerogel in 1.0 M KOH aqueous solution.

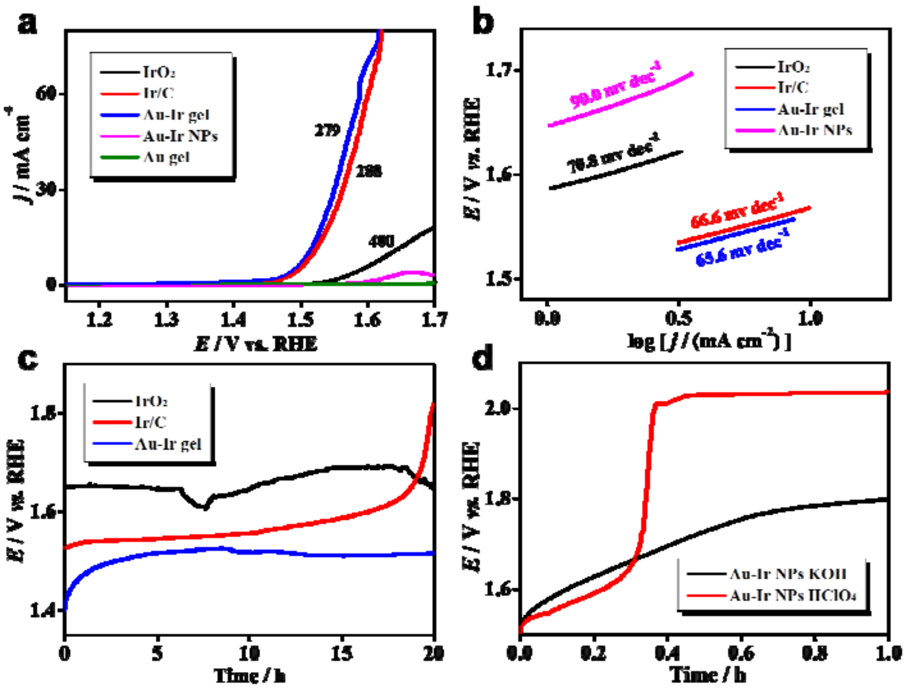

Supplementary Figure 38. Characterization of the electrocatalytic OER properties in acidic medium. (a) LSV curves, (b) Tafel plots, and (c) chronopotentiometric tests (at 2 mA cm^{-2}) of commercial Ir/C, IrO_2, Au aerogel, Au-Ir aerogel, and Au-Ir NPs in 0.1 M $HClO_4$ aqueous solution.

6. Experimental parameters for bi-metallic gels are vaguely presented in the supporting information. Details procedures are needed at least for the newly developed Ru, Rh, Os, and Ir gel systems.

Reply:

Thank you for your suggestion.

All metal gels were created by the same procedures, with the only difference residing in the different metal salt precursors. We have clarified this point in the supporting information as follows:

"All hydrogels were synthesized by either a one-step method or two-step method at ambient temperature (~293 K). For the synthesis of single-metallic gels, the gold system

is taken as an example, while all other single metal gels (*e.g.* Ag, Pd, Pt, Ru, Rh, Os, Ir) were produced by using the respective metal salt precursors following exactly the same one-step method as described below."

Reviewers' comments:

Reviewer #1 (Remarks to the Author):

This reviewer is satisfied with the revised manuscript and recommends publication of this manuscript as is.

Reviewer #2 (Remarks to the Author):

The comments from the reviewers have been replied accordingly. The following issues have to be properly addressed before the acceptance for the publication.

1) The structure of as-prepared bimetallic NMAs are needed to be further investigated, core-shell structure or alloyed structure.
This point is not clearly demonstrated. XPS and XRD can well demonstrate the formation of core-shell structure or alloyed structure.
2) Cycling test can be conducted and then the morphology of the samples before and after cycling test are compared.
Please show the results of cycling test. In addition, Nafion is used for fixing the aerogels during the testing? If so, the presence of Nafion doesn't affect the morphology of the samples?
3) Based on the data in Table S1, it seems that only Au and Pd aerogels can be called as aerogels. This issues have to be addressed again.
a) The surface area of most of aerogels are rather close that of nanoparticle aggregates, and lower than that of nanowire networks. Thus, they are rather similar to aggregates of the nanowire.
b) Moreover, the products shown in Supplementary Figure 28 look like fully aerogels. However, in Figure 1, the intermediate products in the bottle labelled with 4h are located at the bottom. How they floated in the solution from the bottom of the bottle? Can the amount of Au precursor in the bottle produce the corresponding aerogel? The amount of precursors used for synthesis of samples in Figure 1a and Supplementary Figure 28 are equal?

Reviewer #3 (Remarks to the Author):

Authors have addressed several comments raised by this reviewer and others. However, a few more explanations are needed:

(1) Supplementary Figure S2, based on STEM/EDS maps, some of the NMAs produced show primarily core-shell morphology whereas the others show complete alloying behavior. An explanation should be added to the main manuscript regarding these morphological differences and experimental conditions that lead to such subtle changes.

(2) Once again, authors' claim that 59 m2/g surface area obtained for Au gels is a RECORD high value while literature reports on similarly produced Au gels show values of ~50 m2/g. The method reported in this manuscript essentially produces nanoparticle precipitates bound together by electrostatic attractions, which can then be freeze-dried or supercritical-dried to produce "gels". Therefore, these gels typically have similar surface area to those of NP precipitates and claiming "record high" is obviously an over statement. What are the typical surface area of NP precipitates used in EOR and OER reactions (Figure 4, Au-Pd or Au-Ir)? How do they compared to monolithic "gels" reported? Otherwise, what is the surface area of Au NP precipitates that used to produce Au gels with a surface area of 59 m2/g?

(3) Figure 4a-b: Although Au-Pd-1 sample shows ~3-4 times increase in current density compared to

precursor Au-Pd NP precipitates, the If/Ib ratios are similar (0.8-1.0) for all samples tested (NPs and gels). This means significant by product formation (oxidized CH or CHO species), which could poison the catalyst. Can authors comment on this?

Response to reviewer 2's comments

(All changes made are highlighted in blue in the revised manuscript)

Thank you very much for your elaborate comments and advice which will really help us to improve the quality of our present manuscript and future research. We have carefully revised the manuscript according to your comments as follows:

1. The structure of as-prepared bimetallic NMAs are needed to be further investigated, core-shell structure or alloyed structure. This point is not clearly demonstrated. XPS and XRD can well demonstrate the formation of core-shell structure or alloyed structure.

Reply:

Thank you for your suggestion.

XRD data has been shown in **Figure S29**. For both randomly-distributed aerogels (e.g. Au-Pd and Au-Pt) and core-shell-structured aerogels (e.g. Au-Rh and Au-Ir), the two metals cannot be distinguished and only one set of peaks can be found. It might be due to that when the size of both the core and shell metals are very small and the crystallinity is not very high, it is difficult to identify separate metal phases by XRD. In our previous work reported in *Sci. Adv.***2019,** *5*, eaaw4590, we found similar results for multi-metallic aerogels with core-shell, homogeneously-distributed, and heterogeneously-distributed elemental distributions.

Additionally, three typical aerogels (randomly-distributed Au-Pd, core-shell Au-Ir, and core-shell Au-Rh gels) are selected for elemental analysis, which are summarized in the corresponding elemental analysis from XPS data and ICP-OES data in **Table 3**. As also similar to our previous work (*Sci. Adv.***2019,** *5*, eaaw4590), a higher amount ratio of the "shell-metal" is observed by XPS than by ICP-OES in the core-shell-structured Au-Ir and Au-Rh gels, which can be attributed to the surface-sensitivity of the XPS technique. While a slightly higher Pd component was also observed by using XPS compared with ICP-OES, which might be attributed to the perturbation of the elemental distribution of the Au-Pd samples at different detection regions (this can affect the result of XPS).

Therefore, because of the accuracy of these techniques as well as the nanostructured features of the noble metal aerogels, XRD and XPS data are not very appropriate to probe the core-shell structure on this occasion. Nevertheless, the STEM-EDX, which we have

used for detecting the elemental distribution in this work, is regarded as the most direct and common characterization method (such as reported in *Sci. Adv.***2019,** *5*, eaaw4590, *Angew. Chem. Int. Ed.* **2018**, *57*, 2963-2966., *J. Mater. Chem. A* **2018**, *6*, 7517-7521.). STEM-EDX has provided a very intuitive and clear hint of the alloying status of multimetallic metal aerogels as shown in **Figure S32**. Therefore, it should be sufficient to verify the core-shell and alloy structure you have mentioned.

According to your suggestions, we have enriched the descriptions of the XRD and XPS as follows:

"However, the elemental distribution cannot be simply identified from XRD patterns (**Supplementary Figure 29**), presumably due to the nanoscale size of two metals. However, from the elemental analysis, the slightly higher proportion of the "shell metal" as determined by X-ray photoelectron spectroscopy (XPS) than by ICP-OES can partially reflect the core-shell structure of the corresponding aerogels due to the surface sensitivity of XPS (**Supplementary Table 3**).[14]"

Supplementary Table 3. Elemental analysis of different alloy aerogels.

	**Au-Pd**	**Au-Ir**	**Au-Rh**
**XPS**	1/1.02	1/0.26	1/0.80
**ICP-OES**	1/1.05	1/0.27	1/0.89

2. Cycling test can be conducted and then the morphology of the samples before and after cycling test are compared. Please show the results of cycling test. In addition, Nafion is used for fixing the aerogels during the testing? If so, the presence of Nafion doesn't affect the morphology of the samples?

Reply:

Thank you for your suggestion.

By using EOR catalysis as an example, we have revealed that the morphology remains almost the same before and after cycling, According to your suggestion, we have added the cycling data in **Supplementary Figure 36e**. Yes, Nafion is included as indicated in the characterization part, so as to be consistent with the real operation conditions. From

our SEM and TEM observations (**Supplementary Figure 36a-d**), it did not affect the morphology of aerogels so much.

The updated **Supplementary Figure 36** is shown as follows.

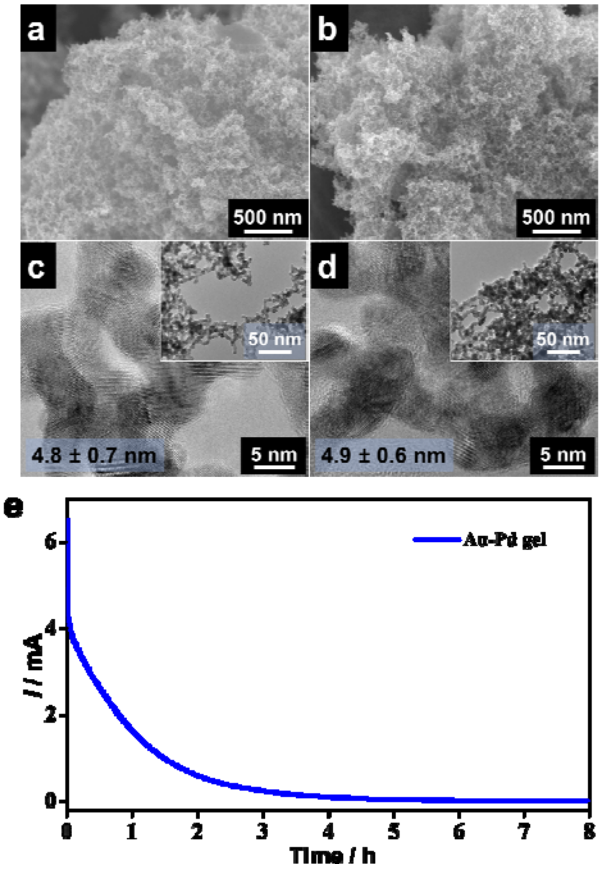

Supplementary Figure 36. (a-d) Evaluation of possible morphology changes of the Au-Pd aerogel (a,c) before and (b,d) after a chronoamperometry test for 8 h, and (e) the corresponding cycling test.

3. Based on the data in Table S1, it seems that only Au and Pd aerogels can be called as aerogels. This issues have to be addressed again.
a) The surface area of most of aerogels are rather close that of nanoparticle aggregates, and lower than that of nanowire networks. Thus, they are rather similar to aggregates of the nanowire.

b) Moreover, the products shown in Supplementary Figure 28 look like fully aerogels. However, in Figure 1, the intermediate products in the bottle labelled with 4h are located at the bottom. How they floated in the solution from the bottom of the bottle?

Can the amount of Au precursor in the bottle produce the corresponding aerogel? The amount of precursors used for synthesis of samples in Figure 1a and Supplementary Figure 28 are equal?

Reply:

Thank you for your suggestion.

a) (1) The difference of the aerogel and aggregate does not lie in their surface areas. For example, activated carbon powder features a BET surface area of thousands of m^2 g^{-1}, which are larger than nearly all reported aerogels, but it is not an aerogel. In our opinion and according to IUPAC, an aerogel should (i) be prepared via a sequential sol-gel process and a drying process (supercritical drying or freeze-drying), (ii) possess 3D networks (it can be made from 0D, 1D, or 2D building blocks) that can offer them a monolithic architecture extending to the macroscale (*e.g.* more than several millimeters). All materials prepared in this work display these features, so we call them aerogels.

   (2) The specific surface areas of the present aerogels are highly dependent on the ligament size. For aerogels featuring a 3D-networked structure and free-standing architecture but with a large ligament size, the BET surface area can be very small (*e.g.* <5 m^2 g^{-1}). We have already proven this ligament-size-dependent change of the BET surface area of gold aerogels in our previous work (*Sci. Adv.***2019,** *5*, eaaw4590).

   (3) For Ag, Pt, Os, and some Au systems, the freshly as-prepared gels feature large ligament sizes; while for Ru, Rh, and Au-Ru, although they have thin ligaments in the gel state, while the freshly prepared aerogels quickly get burned after exposure to air and the ligament size increase considerably (see **Supplementary Table 1**, **Supplementary Figure 3, 26, 28,** and the explanation in the main text "*Ru and Rh hydrogels, once freeze dried, were very likely to spontaneously burn out in air, leaving behind powder-like pieces which were characterized as partially oxidized species with considerably increased ligament sizes and very low SSA*"). Therefore, they exhibit low specific surface areas.

b) (1) Gels can be prepared either in a small scale (from ~5 mL solution) or in a large scale (from 400-800 mL solution). The amount of the precursor solutions in **Figure 1a** and **Supplementary Figure 28** are different, where the former is 5 mL and the latter is ~800 mL. Additionally, in **Figure 1a**, for a clear demonstration, aerogels were made from ~800 mL precursor solutions, because the sample prepared from 5 mL solution will be too small for demonstration. According to your comments, we have indicated the respective volume in the main text and in the SI to for clarification.

(2) During the gelation process, the decomposition of $NaBH_4$ will release a large amount of H_2 bubbles. The bubbles inside the gel network will offer the buoyancy and drive the gel floating in the solution. In the mechanism part, we have also explained the generation of H_2 during the gelation.

According to your suggestions, we have added more descriptions or detailed explanation as follows:

"**Figure. 1** Demonstration and analysis of the gelation process of gold NPs triggered by $NaBH_4$. (a) Photographs of the fabrication process of the gold aerogel, where the wet gel was directly obtained from the $HAuCl_4$ aqueous solution (5 mL) and further freeze-dried to yield the corresponding aerogel. Note for a clear demonstration, the aerogels shown in Figure 1a were prepared from ~800 mL $HAuCl_4$ solution."

"**Supplementary Figure 28.** (a-b) Photographs and (c) TEM images of various bimetallic hydrogels and aerogels, including Ag-Pd, Ag-Rh, Pd-Pt, and Pd-Rh. These gels were prepared from ~800 mL precursor solutions."

"The as-obtained hydrogel floated in the solution, which is attributed to the buoyancy offered by the generated H_2 bubbles from $NaBH_4$ inside the gel networks during the gelation process."

"Despite small surface areas for certain as-prepared materials, they are regarded as aerogels because they feature 3D interconnected networks that offer them monolithic architectures extending to the macroscale (*i.e.* larger than several millimeters)."

Response to reviewer 3's comments

(All changes made are highlighted in blue in the revised manuscript)

Thank you very much for your elaborate comments and advices which will really help us to improve the quality of our present manuscript and future research. We have carefully revised the manuscript according to your comments as follows:

1. Supplementary Figure S2, based on STEM/EDS maps, some of the NMAs produced show primarily core-shell morphology whereas the others show complete alloying behavior. An explanation should be added to the main manuscript regarding these morphological differences and experimental conditions that lead to such subtle changes.

Reply:

Thank you for your suggestion.

This is very interesting phenomenon, because all reactions are performed in the same condition, and the morphology difference is solely dependent on the composition. Currently we do not have a very comprehensive understanding of that, while a few possible reasons were proposed in the main text as follows:

"It is known that silver tends to segregate to the surface of gold–silver alloys due to its lower surface energy,[40] while the segregation behavior of the other three alloys has been less investigated so far. One possible reason may be the slower reaction kinetics of Ru, Rh, and Ir salts compared to that of $HAuCl_4$ (**Supplementary Figure 34**), which will result in the fast formation of gold cores on which the other metal will nucleate and grow.[14] However, further in-depth investigations are needed to unambiguously reveal the underlying mechanisms."

We hope that in future, we can have a separate work which will be focusing on this topic and gaining a deeper understanding by combined experiments and calculations.

2. Once again, authors' claim that 59 m2/g surface area obtained for Au gels is a RECORD high value while literature reports on similarly produced Au gels show values of ~50 m2/g. The method reported in this manuscript essentially produces nanoparticle precipitates bound together by electrostatic attractions, which can then be freeze-dried or supercritical-dried to produce "gels". Therefore, these gels typically have similar surface area to those of NP precipitates and claiming "record high" is obviously an over statement. What are the typical surface area of NP precipitates used in EOR and OER reactions (Figure 4, Au-Pd or Au-Ir)? How do they compared to monolithic "gels" reported? Otherwise, what is the surface area of Au NP precipitates that used to produce Au gels with a surface area of 59 m2/g?

Reply:

Thank you for your suggestion.

(1) For the mentioned paper is previously published by our group (*ACS Nano* 2016, **10**, 2559-2567), where Au gels were prepared by gelating β-cyclodextrin-stabilized gold NPs with dopamine and the corresponding aerogel shows a BET surface area of up to 50.1 m^2 g^{-1} (18 wt.% β-CD remained in that aerogel). The mechanism of the gelation is not clear so far. For our current work, the gel was prepared by using an excessive amount of $NaBH_4$ from either gold salts or from the ligand-stabilized gold NPs, where the gelation mechanism is well discussed in the paper which is mainly due to the salting-out effect of $NaBH_4$. For both works, a branched-nanowire-like networked structure is present, where the gold is bonded continuously by metal bonds. Therefore, the gels are not "*nanoparticle precipitates bound together by electrostatic attractions*". Hence, "*these gels typically have similar surface area to those of NP precipitates*" is not correct. To this end, we only need to compare the specific surface areas of gels, not NP precipitates.

(2) Among all reported gold aerogels and gold foams reported so far, the value reported in our work (59.8 m^2 g^{-1}) is the highest (also see **Supplementary Table 2**), so we say this value is "record-high", and we say "*affording a gold aerogel with a record-high specific surface area of 59.8 m^2 g^{-1}, thus rendering the as-obtained material with the thinnest ligament size and highest SSA among all gold aerogels reported to date*". The "second highest" is 50.1 m^2 g^{-1} reported in "*ACS Nano* 2016, **10**, 2559-2567", but 18 wt.% β-CD remained in that aerogel, so it is not the pure gold aerogel. Others are all below 30 m^2 g^{-1}, most of which are below 10 m^2 g^{-1} (the ligament size of those materials (usually >10 nm) are also much larger than the reported high-surface-area gold gel (~4.8 nm) in this work). Therefore, under this condition, we think the "record-high" is reasonable.

(3) NPs can exhibit a large specific surface area only if they are well separated. However, for pure metal NPs without presence of any ligand or support, they will aggregate driven by the high surface energy and thus leading to a largely reduced surface area. In contrast, the 3D-networked structure of the aerogel can "fix" the structure and suppress the collapse. Therefore, theoretically separated NPs should have a larger specific surface area, but clean large-surface-area NPs are hard to be obtained. Hence, it is meaningful to prepare the self-supported nano-structured aerogels which usually feature a large specific surface area.

(4) We have taken the PVP-capped Au and Au-Ir systems as examples for measuring the specific surface areas of NP precipitates. NP precipitates were prepared by concentrating by ultracentrifugation followed by freeze drying. We have conducted nitrogen adsorption test. From the isotherms shown below, the very small absorbed amount of gas in the adsorption branch suggests a very low surface area, and the BET surface areas of both Au NPs and Au-Ir NPs are too low to be detected. These isotherms are considerably different from **Supplementary Figure 19e** and **Supplementary Figure 31e** (59.8 m^2 g^{-1} and 42.6 m^2 g^{-1} for Au and Au-Ir aerogels, respectively). This further verifies our analysis above.

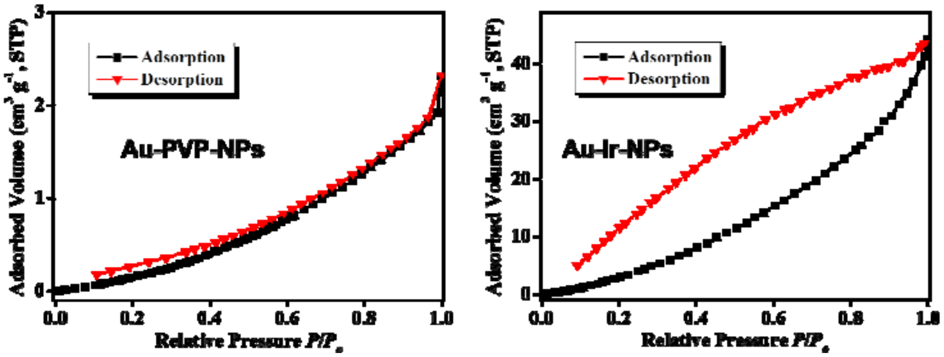

To better compare our results with literature, we have added more details as follows:

"As shown in **Figure 3f-g, Supplementary Table 2**, this value is higher than that of gold/β-cyclodextrin composite aerogels (50.1 m^2 g^{-1})[19] and NH_4SCN-prepared gold aerogels (29.7 m^2 g^{-1}),[14] and remarkably surpasses all other gold aerogels/foams reported to date (<10 m^2 g^{-1} on average).[5,22,37-40]"

3. Figure 4a-b: Although Au-Pd-1 sample shows ~3-4 times increase in current density compared to precursor Au-Pd NP precipitates, the If/Ib ratios are similar (0.8-1.0) for all samples tested (NPs and gels). This means significant by product formation (oxidized CH or CHO species), which could poison the catalyst. Can authors comment on this?

Reply:

Thank you for your suggestion.

According to our previous experience and the current results, the I_f/I_b ratio is highly dependent on the composition (*e.g.*, the introduction of Pt can usually increase the I_f/I_b ratio), while it is not sensitive to the morphology (gels or NP precipitates). In our opinion, the morphology of materials can affect the surface area and thus the number of active sites, but it will not affect the reaction pathways. On the contrary, the change of composition or the facets may affect the interactions between catalysts and reactants/intermediates, thus altering the catalytic mechanisms. Because I_f/I_b ratio can be correlated to the generated intermediates which are highly dependent on the catalytic mechanisms, hence it will not be considerably affected by changing the morphology of the materials. We have added this discussion in the main text as follows:

"The substantially higher I_f compared to other catalysts confirms the high-performance of the Au-Pd aerogel catalyst, presumably coming from the high conductivity of gold, the high catalytic activity of palladium, and the structural attributes (*e.g.* large specific surface areas) provided by the aerogel. The I_f/I_b of the aerogel- and NP-based Au-Pd catalysts are similar, which can be attributed to similar reaction pathways resulting from the similar chemical compositions. The relatively low I_f/I_b suggests that the intermediates generated during operation can deactivate the catalysts seriously, which might be addressed by modulating the compositions as shown elsewhere.[14]"

REVIEWERS' COMMENTS:

Reviewer #2 (Remarks to the Author):

The authors have replied most of the comments raised by the reviewers. However, the following issues are not properly addressed yet. The MS can be accepted for publication after the minor revision.

1) The content ratios of Au and other metals in XRD and XPS results cannot tell the formation of core-shell or alloyed structure. The corresponding peak shifts can tell. For instance, in the XPS spectra of core-shell structured Au-Pd aerogels, due to the difference in their potential, Au peak would negatively shift due to the gaining electrons from Pd and Pd peak would positively shift due to the loss of its electron. However, in the XPS spectrum of alloy structured Au-Pd aerogels, Au peak and Pd peak both would negatively shift due to the further interaction of s-orbital electron of Au with Pd d-orbital electron.
2) The presence of Nation would impact the clarity of the SEM and TEM images as the conductivity of the Nation is not good. However, it seems that the presence of Nations doesn't have any effect on them. Please explain.
3) Cycling test is not the chronoamperometry test (i-t curve). In the cycling test, the electrocatalytic performance of the catalysts is evaluated in the full potential range, instead of one specific potential. Accordingly, please give the SEM and TEM images after the cycling test, instead of the chronoamperometry test.

Reviewer #3 (Remarks to the Author):

Authors have addressed the comments raised by this reviewer. This manuscript can be published.

Response to reviewer 2's comments

(All changes made are highlighted in blue in the revised manuscript)

Thank you very much for your elaborate comments and advice which really help to improve the quality of our present manuscript. We have carefully revised the manuscript according to your comments as follows:

1. The content ratios of Au and other metals in XRD and XPS results cannot tell the formation of core-shell or alloyed structure. The corresponding peak shifts can tell. For instance, in the XPS spectra of core-shell structured Au-Pd aerogels, due to the difference in their potential, Au peak would negatively shift due to the gaining electrons from Pd and Pd peak would positively shift due to the loss of its electron. However, in the XPS spectrum of alloy structured Au-Pd aerogels, Au peak and Pd peak both would negatively shift due to the further interaction of s-orbital electron of Au with Pd d-orbital electron.

Reply:

Thank you for your suggestion.

According to your suggestion, we have compared the peak position of the measured and theoretical values of Au 4f 7/2 and Pd 3d 5/2, respectively. We found that the Au peak shifts negatively from 84.0 eV to 83.9 eV, while the Pd peak shifts positively from 335.1 to 335.4 eV, which may suggest an electron transfer from Pd to Au due to their different electronegativity (2.54 for Au vs. 2.20 for Pd). We have added this in the manuscript as follows:

"Additionally, presumably due to the electronegativity difference of Au (2.54) and Pd (2.20), the peak of Au 4f 7/2 shifts negatively from 84.0 eV to 83.9 eV, while the peak of Pd 3d 5/2 shifts positively from 335.1 to 335.4 eV, suggesting an electron transfer from Pd to Au as a consequence of alloying. In contrast, no considerable peak shift was observed for Au-Ir and Au-Rh aerogels, indicating weak interactions between the two metals."

2. The presence of Nation would impact the clarity of the SEM and TEM images as the conductivity of the Nation is not good. However, it seems that the presence of Nations doesn't have any effect on them. Please explain.

Reply:

Thank you for your suggestion.

We think it might be attributed to the low concentration of the Nafion, as well as the distribution inhomogeneity of Nafion in the samples.

3. Cycling test is not the chronoamperometry test (i-t curve). In the cycling test, the electrocatalytic performance of the catalysts is evaluated in the full potential range, instead of one specific potential. Accordingly, please give the SEM and TEM images after the cycling test, instead of the chronoamperometry test.

Reply:

Thank you for your suggestion.

We have updated the data of cycling and the corresponding SEM/TEM images in the **Supplementary Figure 36**.

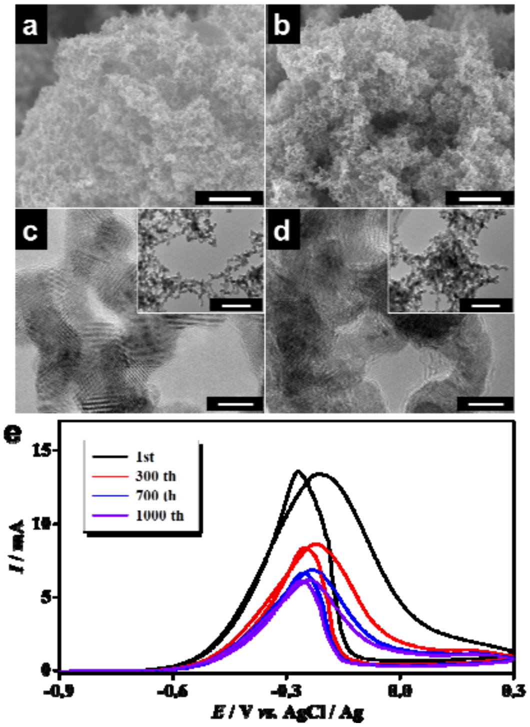

Supplementary Figure 36 | Cycling properties of the Au-Pd aerogels. (a-d) Evaluation of possible morphology changes of the Au-Pd aerogel (a,c) before and (b,d) after 1000

cycles, and (e) the corresponding cycling tests (scan rate: 100 mV s^{-1}). Scale bars in figures a, b are 500 nm, in c, d are 5 nm, and in insets of figures c, d are 50 nm.
